# Molecular Structure Refinement of a ß-Heptapeptide Based on Residual Dipolar Couplings: The Challenge of Extracting Structural Information from Measured RDCs

**DOI:** 10.1021/acs.jpcb.4c06955

**Published:** 2025-03-13

**Authors:** Maria Pechlaner, Wilfred F. van Gunsteren, Lorna J. Smith, Niels Hansen

**Affiliations:** † Institute of Molecular Physical Science, Swiss Federal Institute of Technology, ETH, Zurich CH-8093, Switzerland; ‡ Department of Chemistry, Inorganic Chemistry Laboratory, University of Oxford, South Parks Road, Oxford OX1 3QR, U.K.; § Institute of Thermodynamics and Thermal Process Engineering, University of Stuttgart, Stuttgart D-70569, Germany

## Abstract

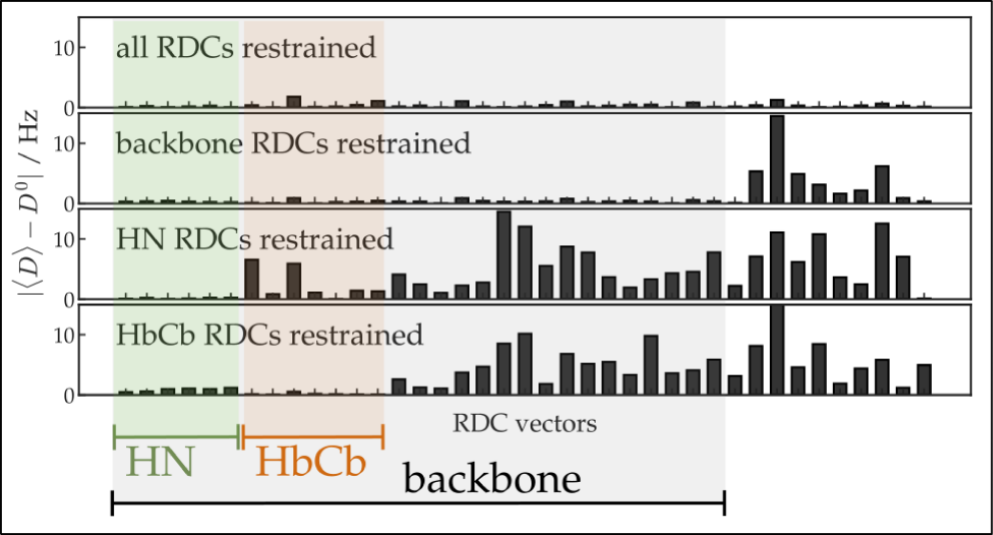

The experimental determination of residual dipolar couplings (RDCs) rests on sampling the rotational motion of a molecule in an environment that induces a slightly nonuniform, unfortunately immeasurable, orientation distribution of the molecule in solution. Averaging over this slightly nonuniform, anisotropic distribution reduces the size of the dipolar couplings (DCs) from the kHz range to the Hz range for the resulting RDCs by a factor of 10^3^ to 10^4^. These features hamper the use of measured RDCs to contribute to the structure determination or refinement of (bio)molecules. The commonly used alignment-tensor (*AT*) methodology assumes that the immeasurable, unknown orientation distribution of the molecule can be expressed in terms of five spherical harmonic functions of order 2. Staying close to experiment, RDCs can, alternatively, be calculated from a molecular simulation by sampling the rotational motion of the molecule (*MRS* method) or, instead, of a vector (*mfv*) representing the magnetic field (*HRS* method). The *AT* and *HRS* methods were applied to a β-heptapeptide solvated in methanol, for which 131 NOE atom–atom distance upper bounds and 21 ^*3*^*J*-couplings derived from NMR experiments are available and, in addition, 39 RDC values obtained for the molecule solvated in methanol with polyvinyl acetate added. In methanol at room temperature and pressure, the molecule adopts a relatively stable helical fold. It appears that MD simulation of the molecule in methanol using the GROMOS biomolecular force field already satisfies virtually all experimental data. Application of RDC restraining shows the limitations caused by the assumptions on which the *AT* and *HRS* methods rest and suggests that experimentally measured RDCs are less useful for molecular structure determination or refinement than other observable quantities that can be measured by NMR techniques. The results illustrate that in structure determination or refinement of a (bio)molecule based on experimentally measured data, it is mandatory (i) to refrain from the vacuum boundary condition and (ii) from torsional-angle restraints that do not account for the multiplicity of the inverse function of the Karplus relation expressing ^3^*J*-couplings in terms of molecular torsional angles, (iii) to allow for Boltzmann-weighted time- or molecule-averaging and, not the least, (iv) to use a force field that has an adequate basis in thermodynamic data of biomolecules.

## 1 Introduction

Over the past 50 years, structure determination of proteins and peptides, in crystalline form based on X-ray or electron diffraction patterns and in solution based on nuclear magnetic resonance (NMR) spectroscopic measurements^1−4^ of a variety of observable quantities, has contributed considerably to the understanding of their function. However, the information density resulting from the different types of measuring techniques, i.e., the ratio of the number of independent, measured values of observable quantities for a molecule and the number of its independent molecular degrees of freedom, is rather diverse. For X-ray diffraction of crystals, it is much higher than for NMR spectroscopic measurements in solution. But, crystallization of peptides or proteins may not be possible. In that case, NMR measurements may provide an alternative source of structural information. Of all techniques available to obtain information on peptides and proteins in solution, NMR shows the highest information density.^5^ However, the number of experimentally derived values of observable quantities for a biomolecule in solution is limited. Thus, one should use all types of observables for which measured values at about the same (thermodynamic) conditions of temperature, pressure, ionic strength, etc., are available, for example, nuclear Overhauser enhancements (NOEs) in the form of NOE atom–atom distance upper bounds, ^3^*J*-couplings, relaxation data in the form of *S*^2^ order parameters, and residual dipolar couplings (RDCs).

All techniques to derive structural information from the measurement of an observable quantity *Q* make use of a relation of *Q* to structure , a function *Q(**)*.^5^ Here, ≡ (,,···,) denotes the 3*N* Cartesian coordinates of the *N* atoms or particles of the molecular system. If an observable quantity *Q* is dependent on the molecular configuration , one may try to derive an expression or function *Q(**)* that approximates the relation between *Q* and . This expression may then be used to obtain molecular structures that are compatible with measured (averaged) values of *Q*, i.e.,
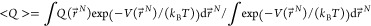
1

<*Q*> is a Boltzmann-weighted average of  over the 3*N*-dimensional configuration space of the molecular system. This means that <*Q*> constitutes an average over a statistical-mechanical ensemble of molecular configurations. The weights are proportional to exp(−*V*()*/*(*k*_B_*T*)), where *V*() indicates the energy of a molecular configuration or structure , *k*_B_ is Boltzmann’s constant, and *T* is the temperature.

Since virtually all experimental techniques measure an average <*Q*>_space,time_ of *Q* over the molecules (space) in the test tube or crystal and over a time window determined by the type of experiment, the derivation of structural information from a set of measured <*Q*>-values should account for the averaging involved in the measurement. This can be done by applying multimolecule averaging^6^ or time-averaging^7^ structure refinement instead of the commonly used single-structure refinement technique. Application of time-averaging structure refinement to proteins based on X-ray data,^8,9^ NMR NOE,^10,11^ or ^3^*J*-coupling^12^ data showed the protein structural variation to be much larger than observed using single-structure refinement techniques.^5^

For observable quantities *Q*, such as X-ray reflection intensities *I*_*hkl*_, NOEs, or ^3^*J*-couplings, it is possible to formulate a function  relating a *Q*-value to a particular structure . For other observable quantities, such as *S*^2^ order parameters or RDCs, the function relating *Q* to  involves some average over the Boltzmann ensemble of structures in solution, *Q*(<*f*()>), where *f* denotes the function of  that is being averaged.^5^ This means that structure refinement based on such quantities must involve the averaging <*f*()> prior to the averaging <*Q*(<*f*()>)> of the quantity *Q*. An example is *S*^2^ order parameters that can be calculated using an ensemble averaging expression.^13,14^

A dipolar coupling or in short *D*_*k*_ between two nuclear spins *k*_1_ and *k*_2_ in a homogeneous magnetic field  depends on^15−17^ (i) the length ≡ || of the internuclear vector  ≡ –, and (ii) the angle θ_*k*_ of this vector with the magnetic field , e.g., cos(θ_*k*_) = , where  is represented by a two-atomic molecule or two-particle vector rigidly connecting atoms or particles *h*_1_ and *h*_2_, ≡ –, and  is the scalar product of the two vectors  and , and
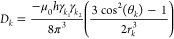
2

Here, the short-hand notation *r*_*κ*_≡  is used, *μ*_*0*_ is the magnetic permeability of vacuum (*ε*_*0*_*c*^2^)^−1^ = 4π × 10^–7^ J s^2^/(C^2^ m) or 1.9425913 × 10^–8^ kJ mol^–1^ ps^2^/(e^2^ nm), *h* is Planck’s constant, 6.626176 × 10^–34^ J s or 0.3990313 kJ mol^–1^ ps, and γ_*i*_ is the gyromagnetic ratio^18,19^ of nucleus *i*. The constant and time-dependent factors in eq 2 can be separated as follows:
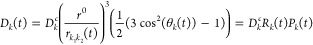
3with
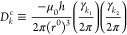
4
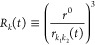
5
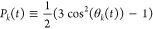
6

The second-order Legendre function *P*_*k*_(θ_*k*_(*t*)) has two zero’s, at the so-called magic angles (|cos(θ)| = 3^–1/2^) 55° and 125°, two maxima of 1 at 0° and 180°, and a minimum of −1/2 at 90°. It is symmetric around θ = 90°. Since the distance between nuclei *k*_1_ and *k*_2_ is generally of the order of 0.1 nm, one may choose *r*^0^ = 0.1 nm, which yields −μ_0_*h*/(2π(*r*^0^)^3^) = −1.3252 × 10^–10^ J^2^ s^3^ C^–2^ m^–4^ or −1.2336 × 10^–6^ (kJ mol^–1^)^2^ ps^3^ e^–2^ nm^–4^. Approximate values of the gyromagnetic ratio for some nuclei can be found in refs (18) and (19). For an N–H pair of nuclei, we find, for example, D_k_^c^(^14^N–^1^H)= −17.37 kHz and *D_k_*^*c*^(^14^N–^1^H)= +24.36 kHz. We note, however, that a dipolar coupling is also influenced by external factors, such as neighboring nuclei.^20^Equation 2 is thus an approximation.

In a measurement of RDCs, the value of *D*_*k*_ in eq 2 is averaged over molecules and an experimentally determined time period. A dipolar coupling *D*_*k*_ generally involves atoms in a molecule that are covalently bound to each other and so move at a relatively high frequency compared to that of the rotational motion of the molecule because the sampling of the molecular rotational degrees of freedom occurs on a much longer time scale. Thus, one may use the assumption that the fluctuation of the distance  is not coupled to the rotational motion of the RDC vector  with respect to the magnetic-field direction . So the averaging of *D*_*k*_ may be separated into an average over (*r*_*k*1*k*2_)^−3^ and one over the second-order Legendre function of θ_*k*_,
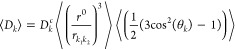
7

The average <(*r*^0^/)^3^> will be close to one in case  is the fluctuating bond length and *r*^0^ is the value close to its average. For an iso-tropically tumbling molecule, the average over the function *P*_*k*_*(*θ_*k*_*)* will be zero,
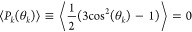
8leading to <*D*_*k*_> = 0.

However, by introducing an anisotropy in the rotational tumbling of the molecule, one would have <*D*_*k*_> ≠ 0, and the values of <*D*_*k*_> would yield values of the average <*P*_*k*_(θ_*k*_)> and some information about values of the angles θ_*k*_.

Experimentally, such an anisotropy can be induced in different ways:^17^ (i) using the paramagnetic susceptibility of a molecule, (ii) using electrostatic interactions with a molecule, or (iii) by immersing the molecule in a medium that contains some order that will influence the angular distribution *P*(θ) of the angle θ of some axis in the molecule with the direction of the magnetic field. In this way, small values of <*D*_*k*_>, called *residual* dipolar couplings (RDCs), of the order of Hz, that is 10^3^–10^4^ smaller than the values of the dipolar couplings *D*_*k*_ themselves, which are of the order of kHz, can be obtained. Unfortunately, the size and shape of the experimentally induced anisotropy in rotational distribution *P*(θ) cannot be determined. This anisotropy will be different for different media, leading to rather different <*D*_*k*_>-values for a molecule dissolved in different media,^21^ which does not necessarily imply though that the structure of the molecule and its internal dynamics would be different.

The common way to handle this problem is to exclude the rotational degrees of freedom of the molecule from the sampling in a calculation of RDCs and instead assuming a particular orientation distribution, that is, in terms of five spherical harmonic functions of order 2, of the molecule with respect to the direction of the magnetic field. This involves the assumption that the overall rotation of the molecule is decoupled from its internal motions. In addition, it is often assumed that the orientation or alignment distribution of the molecule has the same effect on different <*D*_*k*_>-values (RDCs), i.e., that the molecule is rigid.

A recently proposed alternative approach^22,23^ to use measured RDC values for structure determination or refinement of molecules is based on extensively sampling the rotational degrees of freedom of the molecule by molecular dynamics (MD) or stochastic dynamics (SD) simulation in combination with sampling the molecule-internal and solvent degrees of freedom. By using rotational sampling, as occurs in the experiment leading to observable RDCs, the algorithms stay close to the experiment and avoid the use of an alignment tensor, and, thus, the assumptions (i) that the orientation distribution has a particular shape, (ii) that the overall rotation of the molecule is decoupled from its internal motions, and (iii) that the molecule is rigid. It thus allows for molecular flexibility but requires MD or SD simulations of the molecule that are sufficiently long to sample the rotational distribution well, i.e., long enough to reduce the <*D*_*k*_> values, which should for infinite sampling become zero (if there is no orientation bias), to beyond a value that is about 10^–3^ to 10^–4^ of the values of . Use of the method of ref (22), which is based on sampling of the rotational degrees of freedom of the molecule by rotation of the whole molecule (molecule rotational sampling: *MRS*) without invoking assumptions on the shape of the orientation distribution, requires MD or SD simulations of microseconds or longer.^22^ Use of the method of ref (23), which is based on sampling the rotational motion of a magnetic-field (*H*) vector in the form of two atoms or particles connected by a rigid bond by SD simulation (magnetic-field rotational sampling: *HRS*), requires less long simulations,^23^ and it also does not assume a particular shape of the orientation distribution. Inclusion of a penalty function that drives the calculated RDCs toward the measured ones in the potential energy function of such a simulation then generates an orientation distribution of the molecule (*MRS*) or of the magnetic-field vector (*HRS*) and a Boltzmann-weighted conformational ensemble of the molecule more or less compatible with the given set of (measured) RDC values.^22,23^

The rotational motion of the two-particle magnetic-field vector in the *HRS* method samples its rotational distribution much faster than an SD simulation of the protein or peptide in vacuo in the *MRS* method, let alone an MD simulation of a protein or peptide in explicitly simulated solvent. An SD simulation of the magnetic-field vector easily covers nano- or microseconds, and the stochastic forces induce better sampling of its rotational degrees of freedom than MD simulation in vacuo.

The basic idea of the *HRS* algorithm is a hybrid simulation scheme, in which simulation of the molecular system (*msy*), i.e., SD simulation of a molecule in vacuo or MD simulation of a molecule in explicit solvent, with RDC restraining alternates with SD simulation of a magnetic-field vector (*mfv*) in vacuo with RDC restraining. From the *mfv* SD simulations, rotationally sampled RDC values for each SD or MD configuration of the molecule (in vacuo or solution, respectively) in the *msy* simulation are obtained, which are then used to calculate time-averaged RDC values in the *msy* SD or MD simulations of the molecule. The RDC restraining in the *mfv* SD simulations, of *N*_*mfv*_ time steps each, generates an anisotropic rotational distribution of the magnetic-field vector (equivalent to the anisotropic rotational distribution of the solute in the method of ref (22)), leading to nonzero RDC values for each molecular configuration. The latter RDC values can then, time-averaged over the molecular configurations, be used to calculate the RDC-restraining forces in the *msy* SD or MD simulation. Thus, the rotational averaging of the molecule is accounted for by time-averaging over magnetic-field orientations in the *mfv* SD simulation, while the configurational averaging of the molecule, important when the molecule is flexible, is accounted for in the *msy* SD simulation in vacuo or the *msy* MD simulation of the molecule in an explicit solvent.

Here, the *AT* method of calculating RDC values by minimizing the difference between calculated and (measured) target RDC values and the *HRS* method of restraining toward (measured) target RDC values in an MD or SD simulation are investigated by application to a β-heptapeptide solvated in methanol.^24^ Six issues are addressed.

1. Is the dominant helical conformation of the peptide in methanol solution, as confirmed by NOE atom–atom distance upper bounds derived from experimental ROESY spectra and ^3^*J*-coupling data,^25^ similar to the dominant conformation as inferred from RDC values obtained from a measurement^24^ of the peptide in methanol with stretched polyvinyl acetate added? In other words, may experimental data obtained under different environmental conditions be combined in a single structure-refinement restraining procedure?
2. Are measured RDC values offering significant structural information for structure refinement of a molecule in solution? This is in view of the required minimization (*AT* method) of the difference between calculated and (measured) target RDC values, or of the RDC restraining of the magnetic-field vector (*mfv*) toward the (measured) target RDC values (*HRS* method) when calculating RDC values.
3. How severe are the limitations of the commonly used alignment-tensor (*AT*) method in single-structure refinement based on RDCs? In other words, how do the *AT*-inherent assumptions of (1) an orientation distribution of the molecule in terms of spherical harmonic functions of order 2, (2) rigidity, and (3) absence of coupling between rotational and internal motions of the molecule affect the resulting molecular structure?
4. Does RDC restraining of the peptide in vacuo lead to deformation of the molecule, i.e., a different conformational ensemble, compared to RDC restraining of the peptide in methanol solution? In other words, is structure refinement based on RDC restraining in vacuo appropriate?
5. Does the commonly used single-structure refinement procedure offer a genuine picture of the conformational ensemble of a molecule in solution? In other words, may molecular motion be ignored when determining molecular structure?
6. How important is the quality of the molecular model or force field used in structure refinement based on NMR data? In other words, is the resulting structure primarily determined by the NMR data or by the molecular model or force field applied?

## 2 RDCs as Restraints in Molecular Simulation or Modeling

### 2.1 Choice of Restraining Function in the *HRS* Method

A simple RDC-restraining function, continuous with a continuous derivative, is a quadratic one with a flat bottom of size 2*ΔD*^*fb*^ that allows for a penalty-free range of deviations ± *ΔD*^*fb*^ of the average <*D*_*k*_>-value from its target (measured) value . In an SD or MD simulation, the time average, ≡ <*D*_*k*_>*_t_*, is used. For large deviations of  from , larger than *ΔD^fb^* + *ΔD*^*h*^, the restraining function is chosen to be linear in order to avoid large restraining forces and energies. In analogy to the flat-bottom restraining function for NOEs, ^3^*J*-couplings and *S*^2^ order parameters,^5^ the corresponding function for RDCs would be for  > ,
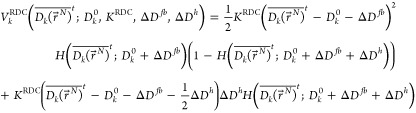
9aand for  < ,
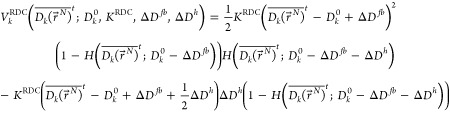
9bwith the Heaviside step function  defined by
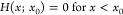
10a
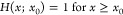
10b

The corresponding force on , that is, the negative of the derivative of  with respect to  is then for  > ,
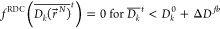
11a
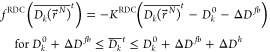
11b
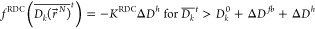
11cand for  < ,
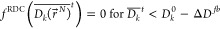
11d
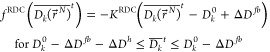
11e
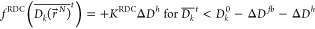
11f

To obtain the force on particle *i*, these expressions are multiplied by , the derivative of  with respect to .

For a set of *N*_RDC_*D*_*k*_-values, we have
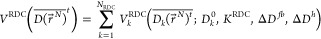
12

Here, *K*^RDC^ is the restraining force constant.

The GROMOS force fields^26−30^ treat aliphatic CH_n_ moieties as united atoms. If C–H RDCs are to be calculated, the implementation allows for the use of a virtual H atom.^27,31,32^ Expressions for the RDC-restraining forces of the *HRS* method can be found in ref (23).

### 2.2 Accounting for Time Averaging in the HRS Method

An RDC is intrinsically an average (over the rotation of the molecule) quantity, <*D*_*k*_>, that cannot be linked to a single configuration. When restraining an averaged quantity in an MD or SD simulation, the averaging cannot be over the whole simulation period *t*, because the contribution of the configuration at time *t* to the average would tend to zero for *t* approaching infinity, which implies that the restraining force, which can be applied only to the current configuration at time *t*, would also tend to zero for *t* approaching infinity. For this reason, instead of using the time average ≡ < *D*_*k*_ > *_t_*, an exponential memory-relaxation function is used in the time-average, , that is used in the restraining function,^7,10^
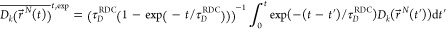
13where  is the memory relaxation time, which determines the averaging time period. As discussed before, the averaging over the two time-dependent factors in eq 3 may be well approximated, due to the different relevant time scales, by a separation of the averaging, see eq 7, so
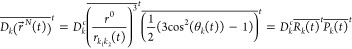
14

If an RDC is due to two atoms connected by a covalent bond and this bond is kept fixed during a simulation, as is done in the current study, the averaging of *R*_*κ*_(*t*) can be omitted, i.e.,  can be used. The time-averaged quantity  in eq 14 can be used either as a standard time average  or as an exponentially damped one,
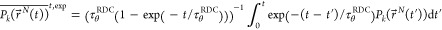
15with  its memory relaxation time.

In a simulation, the atomic configurations are separated by a time step or interval *Δt*, so in discretized form we have for the *n*^th^ time step for the exponentially damped time-averaged quantity in eq 15
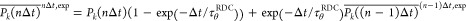
16and using this equation
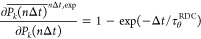
17

Generally, the memory relaxation time  is chosen to be much longer than the MD or SD integration time step *Δt*,
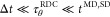
18where *t*^MD,SD^ is the length of the MD or SD simulation. This means that the derivative eq 17 can be approximated by .

### 2.3 Calculation of RDCs Using the Rotational Sampling (*HRS*) Method

The calculation of RDC values is straightforward using  in eq 7 and averaging over SD or MD trajectory structures. In the RDC-restraining simulations, the RDC-restraining forces on the molecule are calculated at every time step using an exponential damping factor in the average, using  for . These two types of RDC values, calculated from  on the one hand and from on the other, will differ. In order to analyze all trajectories in the same way,  or rather  is used when reporting RDC values calculated from the trajectories.

When using the rotational sampling *MRS* or *HRS* methods in the presence of a single RDC restraint or when applying a large RDC-restraining force, the orientation distribution of the molecule may show two peaks at the two magic angles.^22,23^ Instead of reducing the rotationally averaged dipolar coupling by sampling the complete 180° range of angles between the magnetic field and some axis in the molecule, such a reduction may more easily be achieved by sampling angles close to the magic-angle values. When applying more than one RDC restraint, of which the bond vectors appear to have different orientations in the molecule, this artifact may disappear.^22,23^ However, in case the various target RDC values of a set of more than a few RDC restraints are inconsistent with a molecular structure, the rotational-sampling algorithms may also lead to an enhanced sampling of orientation angles around the magic-angle values, i.e., to restricted sampling of the rotational degrees of freedom of the molecule. Thus, the orientation distribution of the molecule resulting from RDC restraining should not be dominated by angle values around the magic angles.

### 2.4 Calculation of RDCs Using the Alignment-Tensor (*AT*) Approach

The formulas to calculate RDCs and the RDC-restraining forces when applying the alignment-tensor approach have been given in refs (33) and (34). There, it is assumed that the bonds of the molecule are kept rigid, as is the case in the present study. In ref (34), the alignment-tensor approach was extended with the possibility to allow time-averaging of five quantities determining the alignment tensor, using a memory relaxation time , and to allow time-averaging of RDCs using a memory relaxation time . In the present study, only the standard, commonly used alignment-tensor approach is applied, i.e., without any time-averaging (= = 0 ps).

## 3 Hybrid Algorithm for MD of a Molecule in Explicit Solvent and SD In Vacuo for the Magnetic-Field Vector

In the hybrid *mfv*/*msy* algorithm,^23^ an *mfv* SD simulation in vacuo of *N*_*mfv*_ time steps *Δt* is performed for the two particles of the magnetic-field vector  before each time step *Δt* of the *msy* SD or MD simulation of *N*_*msy*_ time steps of the molecule in vacuo (SD, *N* = *N^p^*) or of the system of *N^p^* solute atoms and *N^s^* solvent atoms (MD) in a periodic box (*N* = *N^p^* + *N^s^*). The positions and velocities of the atoms at time *t*_*n*_ in the *msy* SD or MD simulation of the molecular system are denoted as  or  in case it is clear that molecular coordinates in the SD or MD simulation of the molecular system are meant, and  or , respectively. The memory relaxation time for the angle θ when time averaging in the *msy* SD or MD simulation of the molecular system is , and *n* is the *msy* SD or MD step counter. The positions and velocities of the two particles of the magnetic-field vector at time *t*_*m*_ in the *mfv* SD simulation are denoted as  or  and  or , respectively. The memory relaxation time for the angle θ when time averaging in the *mfv* SD simulation of the magnetic-field vector is , and *m* is the *mfv* SD step counter.

Since there are two types of simulation steps, *mfv* SD steps and *msy* SD or MD steps, the notation of the function *P*_*k*_*(t)*, eq 6, is extended:
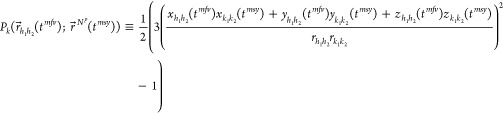
19

The algorithm requires in each *mfv* SD simulation to calculate the averages  and , and also the two averages,  and , of the derivative of  with respect to the particle positions  (*i* = 1, 2) of the magnetic-field vector and of the derivative of  with respect to the atom positions  (*i* = 1, 2, ..., N*^p^*) of the solute molecule, respectively.

The quantities  and  are required to calculate the RDC-restraining force on the magnetic-field vector and the quantities  and  are needed to calculate the RDC-restraining force on the solute molecule, see below.

In the current implementation, the values of the force constant *K*^RDC,*mfv*^ of the RDC-restraining force on the two particles of the magnetic-field vector and of the force constant *K*^RDC,*msy*^ of the RDC-restraining force on the *N*^*p*^ atoms of the molecule can be chosen independently. When *K*^RDC,*msy*^ = 0, the atoms of the molecule are not subjected to the RDC-restraining force, while if in addition *K*^RDC,*mfv*^ > 0, the orientation of the magnetic-field vector will be determined by the RDC-restraining force on its two particles. This then allows the calculation of RDC values for molecular configurations of a non-RDC-restrained trajectory.

In the algorithm,^23^ the quantity  is taken as the average  obtained from an *mfv* SD simulation of *N*_*mfv*_ time steps *Δt* of the magnetic-field vector  using a fixed configuration  of the solute molecule, so
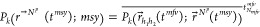
20

The hybrid *mfv*/*msy* SD/MD RDC-restraining algorithm^23^ based on the leapfrog integration scheme^35,36^ may include coupling to a temperature bath (*T*) and a pressure bath (*P*), the application of constraints (*C*), and spatial periodicity (*B*).

The essence of the hybrid *mfv/msy* SD/MD algorithm is that the sampling of the rotational motion of the solute molecule and the corresponding averaging of the *D*_*k*_ values is done by an *mfv* SD simulation of *N*_*mfv*_ time steps *Δt* of the rotational motion of the (two-atomic) magnetic-field vector per time step of the *msy* SD or MD simulation of the solute molecule in vacuo (SD) or in explicit solvent (MD). The sampling of the internal motion of the solute molecule in vacuo or in solution and the corresponding averaging of the *D*_*k*_ values is carried through in the *msy* SD or MD simulation. For large values of *N*_*mfv*_, the rotational sampling of the magnetic-field vector is well converged before the rotationally averaged quantities and are used in the RDC-restraining *msy* SD or MD simulation, in which the molecular configurations are sampled. In this case, the rotational sampling and configurational sampling are carried out more or less separately. For small values of *N*_*mfv*_, the rotational and configurational sampling are performed more or less simultaneously, i.e., are coupled, the extent depending on the value of *N*_*mfv*_.

## 4 Implementation, Molecular Model, Simulation Setup, and Analysis

The simulations were performed using the GROMOS simulation software package.^37−42^ When treating realistic molecular systems, the use of Standard International (SI) units is recommended. Apart from restrictions when storing or printing data in nonexponential format, the GROMOS programs are independent of the chosen units. The units are defined by the ones used for the physical constants and atomic and molecular quantities to be specified in the PHYSICALCONSTANTS block^43^ in the GROMOS data files.^27,44^ It is recommended to use basic units, nanometer (nm) for length, atomic mass unit (u) for mass, picosecond (ps) for time, Kelvin (K) for temperature, and electronic charge (e) for charge. These basic units then determine the units of other quantities, e.g., kJ/mol for energy, kJ/(mol nm) for force, kJ/(mol nm^3^) for pressure, and THz for frequency. If, for example, the non-GROMOS-recommended unit Hz instead of THz is to be used as an input unit for dipolar couplings and kJmol^–1^ Hz^–2^ for the RDC-restraining force constant, the scaling factor 10^12^ should be specified in the RDCRESTRAINTS block.^43^

### 4.1 Molecular Model and Force Fields Used for the Peptide in Simulations In Vacuo or Solvated in Methanol

**Figure 1:**
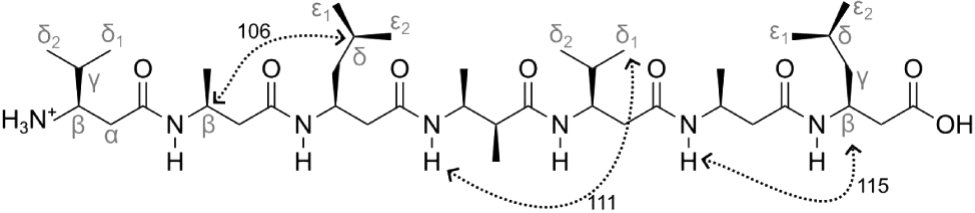
Chemical formula and covalent structure of the ß-heptapeptide H_2_^+^-ß^3^-HVal-ß^3^-HAla-ß^3^-HLeu-(S,S)-ß^3^-HAla(αMe)-ß^3^-HVal-ß^3^-HAla-ß^3^-HLeu-OH.^48^ Dotted lines indicate the atom pairs involved in NOEs 106, 111, and 115, which show persistent NOE distance upper bound violations (see Table 1 and Section 5.10).

The ß-heptapeptide H_2_^+^-ß^3^-HVal-ß^3^-HAla-ß^3^-HLeu-(S,S)-ß^3^-HAla(αMe)-ß^3^-HVal-ß^3^-HAla-ß^3^-HLeu-OH simulated in vacuo or solvated in methanol was used as the test system, see Figure 1. This peptide, consisting of L-ß-amino acids, has been studied extensively during the past decades using CD and NMR spectroscopy and molecular dynamics simulation techniques.^24,25,45−50^ At a temperature of 298 K, it adopts predominantly a (left-handed) *M*-3_14_-helical fold, while at 340 K, a folding/unfolding equilibrium is observed.^46,47^ In the early simulation studies, the GROMOS 43A1 force field^27^ and methanol model were used, but the more recent GROMOS 54A7 force field^29,30^ in combination with its slightly different methanol model^51^ samples more 3_14_-helical conformations, which is due to the slightly enhanced capacity of the backbone NH and CO groups to form hydrogen bonds with each other, and the agreement with the experimentally derived NOE distance upper bounds was slightly improved, while the experimentally derived ^3^*J*-couplings are reproduced equally well.^48^ However, the backbone φ and ψ torsional-angle parameters of the 53A6 force field^28^ should be used when applying the 54A7 force field to β-peptides.^50^

When simulating a peptide or protein in vacuo, the GROMOS biomolecular force field 54B7^27,30^ is to be used. The A-version of a GROMOS force field is the basic force field designed for molecules in explicit water or other solvents. The B-version is derived from the A-version in order to be used for simulating molecules in vacuo, where the dielectric screening effect of the environment is neglected. The atomic charges and van der Waals parameters are changed such that atom charge groups with a nonzero total charge are neutralized while maintaining the hydrogen-bonding capacity of the individual atoms.

When simulating the molecule in vacuo, no nonbonded interaction cutoff was used, whereas when simulating the molecule in a box with methanol molecules applying periodic boundary conditions, the nonbonded interactions were calculated using a triple-range method^52^ with cutoff radii of 0.8/1.4 nm. Short-range (within 0.8 nm) van der Waals and electrostatic interactions were evaluated every time step based on a charge-group pair list.^27,32^ Medium-range van der Waals and electrostatic interactions, between pairs at a distance larger than 0.8 nm and shorter than 1.4 nm, were evaluated every fifth time step (of *Δt* = 2 fs), at which time point the pair list was updated and kept constant between updates. Outside the larger cutoff radius (1.4 nm), a reaction-field approximation^53,54^ with a relative dielectric permittivity of ε_RF_ = 19.8 (methanol at room temperature and pressure) was used.^48,51^ In the *mfv* SD simulations of the magnetic-field vector in vacuo or the *msy* SD simulations of the molecule in vacuo, no periodic boundary conditions were applied, whereas in the *msy* or non-RDC-restrained MD simulations of the molecule solvated in methanol, cubic periodic boundary conditions were used.

The application of bond-length constraints is standard practice in biomolecular simulations and allows for a lengthening of the time step by a factor of 3. All bond lengths were kept rigid with a relative geometric precision of 10^–4^ using the SHAKE algorithm.^55^ This allows for a 2 fs SD or MD time step^56,57^ in the leapfrog algorithm used to integrate the Langevin or Newton equations of motion.^32,35,36^ The bond-length constraints correspond exactly to the bond-stretching force-field terms, and thus, the constraint lengths are taken from these force-field terms. The methanol molecules were kept rigid using the SHAKE algorithm with a relative geometric precision of 10^–4^ for the three distance constraints (O–H, O–CH_3_, H–CH_3_).

### 4.2 Model Used for the Magnetic-Field Vector SD Simulations In Vacuo

A most simple model for the two-particle magnetic-field vector is a molecule of two united atoms, ethane, with a rigid bond. The masses of the two atoms are 15.035 u (a CH_3_ united atom in GROMOS) and the length of the bond is 0.153 nm. The length of the vector is kept constant by using the SHAKE algorithm with a relative geometric precision of 10^–4^. The only potential energy term used is the RDC-restraining one (eqs 9a and 12), so the motion of the vector is determined by the Langevin equation and the RDC-restraining forces. A friction coefficient γ_*i*_^SD^ = 2.4 ps^–1^ is used because this value optimizes the rotational sampling of the magnetic-field vector.^22^

The initial position of the magnetic-field vector is arbitrary, e.g., along the *z*-axis.

The *mfv* SD simulation of the magnetic-field vector  was carried out at 298 K. No removal of center of mass motion was applied.

### 4.3 SD Simulations of the ß-Heptapeptide In Vacuo

When solving the Langevin equations of motion for the ß-heptapeptide in vacuo, the stochastic force *f*_*i*_^*st*^*(t)* and the atomic friction coefficient γ_*i*_ will only be sizable for solute atoms at the surface. Therefore, they are taken dependent on the number of neighboring atoms within the solute,^58^
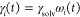
36with
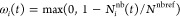
37where *N*_*i*_^nb^*(t)* denotes the number of non-hydrogen neighboring atoms of the solute atom *i* within 0.3 nm radius and *N*^nbref^ was defined as an upper limit of 6 neighboring solute atoms at which solvent forces on solute atom *i* are assumed to vanish. For methanol as a solvent (at room temperature and pressure), γ_solv_ = 60 ps^–1^, and ω_*i*_*(t)* was updated every 1 ps during the *msy* SD simulation.^58^ No cutoff for the nonbonded interaction was used, and *ε*_RF_ = 0. The left-handed *M*-3_14_-helical folded conformation of the peptide was used as the initial structure for the *msy* SD simulations in vacuo. These were performed with a reference temperature of 298 K, maintained by the Langevin equations, which act as a thermostat, and by weak coupling to a heat bath (τ_Τ_ = 0.1 ps),^59^ the latter in order to control the temperature of atoms that have a friction coefficient equal to zero, whose temperature is thus not controlled by the Langevin thermostat. Translational motion of the center of mass of the molecule was removed every 2 ps (1000 time steps).

### 4.4 MD Simulations of the ß-Heptapeptide Solvated in Explicit Methanol

The left-handed *M*-3_14_-helical folded conformation of the peptide was used as the initial structure for the MD simulations. The peptide was solvated in a cubic periodic box with methanol molecules as the solvent. The minimum distance from any peptide atom to the box walls was set to 1.4 nm, resulting in 1096 methanol molecules. The MD simulations were carried out at a temperature of 298 K.^24^ The temperature was kept constant using the weak-coupling algorithm,^59^ separately applied to the solute and the solvent, with the temperature coupling times τ_T_ set to 0.1 ps, and the pressure was maintained at 1 atm using weak coupling^59^ with a pressure coupling time τ_P_ of 0.5 ps and an isothermal compressibility of 4.575 × 10^–4^ (kJmol^–1^ nm^–3^)^−1^.

### 4.5 RDC Restraints

For the ß-heptapeptide, six different sets of experimentally determined RDC values are available,^24^ see Table 1.

**Table 1:** Lists of 42^45^ and 119^24^ NOE Atom–Atom Upper Distance (nm) Bounds Derived from Experiment and the Corresponding *r*^*–6*^ Averaged Distances (in nm) from Unrestrained and RDC-Restrained (*ΔD*^*fb*^ = 2.0 Hz) *t*^*msy*^ = *t*^*AT*^ = 100 ns MD Simulations of the ß-Heptapeptide^a^

					r^–6^ averaged distance (nm)						
NOE Sequence number	NOE H Atom pair		NOE Distance upper bound (nm)		Simulation						Calculation
			Ref (45)	Ref (24)	*MDsol*	*HRSrMDsol*	*ATrMDsol*: *K*^*RDC,AT*^ (kJ mol^–1^ Hz^–2^)				*ATrEMvac*
							0	0.1	1	10	
1	H–N(1)	H–C_ß_(1)	0.28	-	0.26	0.26	0.26	0.26	0.26	0.26	0.25
2	H–C_ß_(1)	H_Re_–C_α_(1)	0.29	0.28	0.28	0.28	0.28	0.28	0.28	0.27	0.29
3	H–C_ß_(1)	H_Si_–C_α_(1)	0.30	0.24	0.24	0.24	0.24	0.25	0.25	0.25	0.23
4	H–N(2)	H_Re_–C_α_(1)	0.24	0.26	0.24	0.24	0.24	0.24	0.26	0.27	0.22
5	H–N(2)	H_Si_–C_α_(1)	0.29	0.33	0.25	0.25	0.25	0.25	0.23	0.23	0.31
6	H–N(2)	H–C_ß_(2)	0.33	-	0.28	0.28	0.28	0.28	0.28	0.28	0.29
7	H–N(2)	H–C_ß_(4)	0.35	0.33	0.36	0.36	0.36	0.36	*0.49*	*0.61*	0.34
8	H–N(2)	H–C_ß_(5)	0.33	0.31	0.31	0.30	0.31	0.31	0.40	*0.79*	0.32
9	H–C_ß_(2)	Me–C_γ_(2)	0.32	0.38	0.24	0.24	0.24	0.24	0.24	0.24	0.24
10	H–C_ß_(2)	H_Si_–C_α_(2)	0.23	0.23	0.24	0.24	0.24	0.24	0.25	0.25	0.24
11	H–N(3)	H_Re_–C_α_(2)	0.22	0.24	0.21	0.22	0.21	0.22	0.25	0.26	0.22
12	H–N(3)	H_Si_–C_α_(2)	0.31	-	0.30	0.28	0.30	0.29	0.24	0.24	0.32
13	H–N(3)	H–C_ß_(3)	0.31	-	0.29	0.29	0.29	0.29	0.27	0.26	0.28
14	H–N(3)	H_Re_–C_α_(3)	0.26	0.29	0.24	0.24	0.24	0.25	0.25	0.26	0.25
15	H–N(3)	H–N(4)	0.38	-	0.38	0.37	0.38	0.37	0.34	0.35	0.24
16	H–N(3)	H–C_ß_(5)	0.34	0.34	0.36	0.35	0.36	0.35	*0.48*	*0.66*	0.31
17	H–N(3)	H–C_ß_(6)	0.32	0.31	0.30	0.30	0.30	0.30	0.37	*0.53*	0.29
18	H–C_ß_(3)	H–C_δ_(3)	0.30	-	0.27	0.27	0.27	0.27	0.27	0.27	0.20
19	H–N(4)	H_Re_–C_α_(3)	0.23	0.24	0.22	0.21	0.22	0.21	0.24	0.26	0.22
20	H–N(4)	H_Si_–C_α_(3)	0.28	0.44	0.28	0.29	0.28	0.29	0.25	0.24	0.34
21	H–N(4)	H–C_ß_(4)	0.29	-	0.29	0.29	0.29	0.29	0.28	0.28	0.28
22	H–N(4)	Me–C_γ_(4)	0.40	0.48	0.29	0.29	0.29	0.29	0.30	0.30	0.33
23	H–N(4)	H–C_ß_(6)	0.32	0.33	0.36	0.36	0.36	0.37	*0.45*	*0.43*	0.32
24	H–N(4)	H–C_ß_(7)	0.37	0.31	0.30	0.29	0.30	0.29	*0.51*	*0.73*	0.32
25	H–C_ß_(4)	H_Re_–C_α_(1)	0.26	0.23	0.25	0.25	0.25	0.25	0.36	*0.52*	0.27
26	H–N(5)	H–N(4)	0.37	-	0.38	0.38	0.38	0.38	0.38	0.39	0.34
27	H–N(5)	H_Re_–C_α_(4)	0.22	0.23	0.22	0.22	0.22	0.22	0.21	0.22	0.22
28	H–N(5)	Me–C_δ_(4)	0.45	0.52	0.35	0.35	0.35	0.35	0.36	0.36	0.36
29	H–N(5)	H–C_ß_(5)	0.35	-	0.28	0.28	0.28	0.28	0.28	0.27	0.29
30	H–N(5)	H_Re_–C_α_(5)	0.25	0.28	0.25	0.25	0.25	0.24	0.25	0.25	0.26
31	H–N(5)	H–N(6)	0.35	-	0.40	0.39	0.40	0.40	0.45	0.44	*0.46*
32	H–C_ß_(5)	H_Re_–C_α_(2)	0.23	0.23	0.26	0.26	0.26	0.26	*0.35*	*0.60*	0.20
33	H–C_ß_(5)	H–C_γ_(5)	0.26	0.25	0.25	0.25	0.25	0.25	0.25	0.25	0.28
34	H–C_ß_(5)	H_Si_–C_α_(5)	0.25	0.23	0.24	0.24	0.24	0.24	0.24	0.25	0.24
35	H–N(6)	H–C_ß_(6)	0.29	-	0.28	0.28	0.28	0.28	0.27	0.28	0.29
36	H–N(6)	H_Re_–C_α_(6)	0.25	0.27	0.24	0.24	0.24	0.24	0.26	0.26	0.25
37	H–N(6)	H_Re_–C_α_(5)	0.22	0.25	0.23	0.22	0.23	0.23	0.28	0.28	0.25
38	H–C_ß_(6)	H_Re_–C_α_(3)	0.25	0.23	0.25	0.25	0.25	0.25	*0.39*	*0.41*	0.21
39	H–C_ß_(6)	H_Si_*–*C_α_(6)	0.26	0.23	0.24	0.24	0.24	0.24	0.25	0.25	0.25
40	H–N(7)	H_Re_–C_α_(6)	0.24	0.26	0.24	0.24	0.24	0.24	0.27	0.25	0.22
41	H–N(7)	H–C_ß_(7)	0.30	-	0.28	0.28	0.28	0.28	0.27	0.28	0.29
42	H–N(7)	H_Re_–C_α_(7)	0.27	0.27	0.25	0.25	0.25	0.25	0.25	0.25	0.23
43	H–C_γ_(1)	H_Si_–C_α_(1)	-	0.27	0.26	0.26	0.26	0.25	0.26	0.25	0.26
44	H–C_γ_(1)	H_Re_–C_α_(1)	-	0.31	0.30	0.30	0.30	0.30	0.30	0.31	0.28
45	H–C_ß_(1)	H–C_γ_(1)	-	0.24	0.24	0.24	0.24	0.24	0.24	0.24	0.24
46	H_Si_–C_α_(2)	Me–C_γ_(2)	-	0.41	0.29	0.29	0.29	0.29	0.28	0.28	0.29
47	H_Re_–C_α_(2)	Me–C_γ_(2)	-	0.41	0.29	0.29	0.29	0.29	0.31	0.31	0.30
48	H_Re_*–*C_α_(2)	H–N(2)	-	0.28	0.25	0.25	0.25	0.25	0.25	0.26	0.24
49	H–C_ß_(2)	H_Re_–C_α_(2)	-	0.31	0.29	0.28	0.29	0.29	0.27	0.26	0.29
50	Me–C_γ_(2)	H–N(2)	-	0.51	0.30	0.30	0.30	0.30	0.29	0.29	0.32
51	H_Si_–C_α_(3)	H–C_ß_(3)	-	0.23	0.24	0.24	0.24	0.24	0.24	0.24	0.24
52	H_Si_–C_α_(3)	H–C_δ_(3)	-	0.29	0.23	0.22	0.23	0.23	0.24	0.24	0.35
53	H_Si_–C_α_(3)	H_Si_–C_γ_(3)	-	0.27	0.33	0.34	0.33	0.34	0.31	0.31	0.35
54	H_Si_–C_α_(3)	Me–C_ε2_(3)	-	0.54	0.42	0.42	0.42	0.42	0.43	0.44	0.52
55	Me–C_ε1_(3)	H–C_ß_(3)	-	0.41	0.31	0.30	0.31	0.31	0.32	0.32	0.37
56	H–C_ß_(3)	Me–C_ε2_(3)	-	0.47	0.35	0.36	0.35	0.36	0.33	0.33	0.40
57	H–C_ß_(3)	H_Re_–C_γ_(3)	-	0.31	0.27	0.27	0.27	0.27	0.26	0.26	0.28
58	H–C_ß_(3)	H_Si_–C_γ_(3)	-	0.27	0.25	0.25	0.25	0.25	0.26	0.26	0.28
59	H–C_δ_(3)	H–N(3)	-	0.38	0.37	0.38	0.37	0.38	0.32	0.30	0.43
60	Me–C_ε1_(3)	H_Si_–C_γ_(3)	-	0.42	0.28	0.28	0.28	0.28	0.28	0.28	0.35
61	H_Re_–C_γ_(3)	H–C_α_(3)	-	0.35	0.26	0.27	0.26	0.26	0.25	0.26	0.24
62	H_Re_–C_γ_(3)	H_Re_–C_α_(3)	-	0.26	0.24	0.24	0.24	0.24	0.26	0.26	0.24
63	H_Re_–C_γ_(3)	H–C_δ_(3)	-	0.24	0.25	0.25	0.25	0.25	0.26	0.26	0.28
64	H_Si_–C_γ_(3)	H–C_δ_(3)	-	0.26	0.27	0.27	0.27	0.27	0.26	0.26	0.27
65	H_Re_–C_γ_(3)	Me–C_ε1_(3)	-	0.47	0.34	0.34	0.34	0.34	0.32	0.32	0.26
66	H_Re_–C_γ_(3)	H–N(3)	-	0.34	0.28	0.28	0.28	0.28	0.29	0.29	0.35
67	H_Si_–C_γ_(3)	H_Re_–C_α_(3)	-	0.30	0.30	0.30	0.30	0.29	0.29	0.30	0.31
68	H_Si_–C_γ_(3)	H–N(3)	-	0.43	0.27	0.28	0.27	0.28	0.26	0.26	0.29
69	H_Re_–C_α_(4)	Me–C_γ_(4)	-	0.40	0.28	0.28	0.28	0.28	0.28	0.28	0.28
70	H–N(4)	H_Re_–C_α_(4)	-	0.29	0.25	0.25	0.25	0.25	0.30	0.32	0.27
71	H–C_ß_(4)	H_Re_–C_α_(4)	-	0.28	0.28	0.28	0.28	0.28	0.25	0.24	0.29
72	H–C_ß_(4)	Me–C_δ_(4)	-	0.40	0.29	0.29	0.29	0.29	0.28	0.27	0.28
73	H–C_ß_(4)	Me–C_γ_(4)	-	0.38	0.24	0.24	0.24	0.24	0.24	0.24	0.24
74	H_Si_–C_α_(5)	Me–C_δ2_(5)	-	0.41	0.33	0.33	0.33	0.32	0.32	0.32	0.34
75	H–C_γ_(5)	H_Si_–C_α_(5)	-	0.28	0.26	0.26	0.26	0.27	0.26	0.26	0.25
76	H_Re_–C_α_(5)	Me–C_δ2_(5)	-	0.45	0.34	0.34	0.34	0.35	0.35	0.36	0.41
77	H_Re_–C_α_(5)	H–C_γ_(5)	-	0.28	0.27	0.27	0.27	0.28	0.28	0.29	0.23
78	H–C_ß_(5)	H_Re_–C_α_(5)	-	0.31	0.29	0.29	0.29	0.29	0.28	0.28	0.29
79	H–C_ß_(5)	Me–C_δ2_(5)	-	0.39	0.30	0.30	0.30	0.30	0.30	0.30	0.26
80	Me–C_δ2_(5)	H–N(5)	-	0.48	0.33	0.33	0.33	0.34	0.32	0.32	0.45
81	Me–C_δ1_(5)	H–C_γ_(5)	-	0.36	0.24	0.24	0.24	0.24	0.24	0.24	0.24
82	H–C_γ_(5)	H–N(5)	-	0.32	0.28	0.28	0.28	0.28	0.28	0.27	0.31
83	H_Si_–C_α_(6)	Me–C_γ_(6)	-	0.41	0.28	0.28	0.28	0.28	0.28	0.29	0.28
84	H_Re_–C_α_(6)	H–C_ß_(6)	-	0.31	0.28	0.28	0.28	0.28	0.26	0.25	0.29
85	Me–C_γ_(6)	H_Re_–C_α_(6)	-	0.34	0.30	0.30	0.30	0.30	0.31	0.31	0.30
86	H–C_ß_(6)	Me–C_γ_(6)	-	0.38	0.24	0.24	0.24	0.24	0.24	0.24	0.24
87	Me–C_γ_(6)	H–N(6)	-	0.48	0.29	0.29	0.29	0.29	0.29	0.29	0.30
88	H_Si_–C_α_(7)	H–C_ß_(7)	-	0.25	0.25	0.25	0.25	0.25	0.25	0.25	0.25
89	H_Si_–C_α_(7)	H–C_δ_(7)	-	0.27	0.23	0.23	0.23	0.23	0.24	0.24	0.33
90	H_Si_–C_γ_(7)	H_Si_–C_α_(7)	-	0.26	0.30	0.30	0.30	0.30	0.29	0.30	0.34
91	H_Si_–C_α_(7)	H–N(7)	-	0.35	0.32	0.32	0.32	0.32	0.32	0.32	0.35
92	H_Re_–C_γ_(7)	H_Re_–C_α_(7)	-	0.28	0.27	0.27	0.27	0.27	0.27	0.27	0.27
93	H_Re_–C_α_(7)	H_Si_–C_γ_(7)	-	0.28	0.31	0.31	0.31	0.31	0.31	0.31	0.33
94	H–C_ß_(7)	Me–C_ε2_(7)	-	0.39	0.33	0.33	0.33	0.33	0.32	0.33	0.34
95	H_Re_–C_γ_(7)	H–C_ß_(7)	-	0.29	0.26	0.26	0.26	0.26	0.26	0.26	0.28
96	H–C_ß_(7)	H_Si_–C_γ_(7)	-	0.26	0.26	0.26	0.26	0.26	0.26	0.26	0.27
97	H–C_δ_(7)	H–N(7)	-	0.36	0.33	0.33	0.33	0.33	0.32	0.31	0.45
98	Me–C_ε2_(7)	H–N(7)	-	0.64	0.48	0.47	0.48	0.48	0.47	0.46	0.47
99	Me–C_ε1_(7)	H–C_δ_(7)	-	0.35	0.24	0.24	0.24	0.24	0.24	0.24	0.24
100	Me–C_ε2_(7)	H_Si_–C_γ_(7)	-	0.42	0.32	0.32	0.32	0.32	0.32	0.32	0.27
101	Me–C_ε1_(7)	H_Si_–C_γ_(7)	-	0.41	0.28	0.28	0.28	0.28	0.28	0.28	0.32
102	Me–C_ε1_(7)	H_Re_–C_γ_(7)	-	0.39	0.33	0.33	0.33	0.33	0.32	0.32	0.27
103	H_Re_–C_γ_(7)	H*–*N(7)	-	0.32	0.28	0.28	0.28	0.28	0.29	0.29	0.34
104	H_Si_–C_γ_(7)	H–N(7)	-	0.39	0.25	0.25	0.25	0.25	0.25	0.25	0.28
105	H–C_ß_(1)	H–N(2)	-	0.48	0.42	0.41	0.42	0.41	0.37	0.36	0.44
106	H*–*C_ß_(2)	H–C_δ_(3)	-	0.44	*0.65*	*0.62*	*0.65*	*0.59*	0.42	0.43	0.54
107	H–C_ß_(2)	H–N(3)	-	0.41	0.43	0.42	0.43	0.41	0.30	0.27	0.42
108	H–N(2)	H–C_ß_(3)	-	0.45	0.48	0.48	0.48	0.48	0.54	0.55	0.44
109	H–C_ß_(4)	H–N(5)	-	0.42	0.44	0.44	0.44	0.44	0.43	0.34	0.45
110	H–N(4)	H–C_ß_(5)	-	0.45	0.47	0.47	0.47	0.47	0.44	0.44	0.40
111	H–N(4)	Me–C_δ1_(5)	-	0.57	*0.70*	*0.70*	*0.70*	*0.69*	0.66	0.64	0.58
112	H_Si_–C_α_(5)	H–N(6)	-	0.34	0.27	0.28	0.27	0.27	0.23	0.23	0.27
113	H–C_ß_(5)	H–N(6)	-	0.43	0.42	0.44	0.42	0.41	0.27	0.26	0.44
114	H–N(5)	H–C_ß_(6)	-	0.44	0.50	0.50	0.50	0.51	*0.56*	*0.56*	0.52
115	H–N(6)	H–C_ß_(7)	-	0.42	*0.54*	*0.54*	*0.54*	*0.54*	*0.54*	0.52	0.50
116	H–C_ß_(6)	H–N(7)	-	0.42	0.39	0.41	0.39	0.39	0.31	0.28	0.42
117	H–C_γ_(1)	H–C_ß_(4)	-	0.35	0.39	0.40	0.39	0.39	*0.56*	*0.67*	0.42
118	H–N(2)	Me–C_δ_(4)	-	0.60	0.48	0.48	0.48	0.48	0.58	0.69	0.42
119	Me–C_γ_(2)	H–C_ß_(5)	-	0.44	0.37	0.37	0.37	0.37	0.48	*0.65*	0.38
120	Me–C_γ_(2)	Me–C_δ2_(5)	-	0.63	0.45	0.46	0.45	0.44	0.57	*0.79*	0.37
121	H–N(2)	Me–C_δ2_(5)	-	0.58	0.48	0.48	0.48	0.48	0.60	*0.87*	0.49
122	H–N(2)	H–C_γ_(5)	-	0.53	0.48	0.48	0.48	0.46	0.56	*0.86*	0.58
123	H–N(3)	Me–C_δ2_(5)	-	0.60	0.65	0.64	0.65	0.63	*0.77*	*0.90*	0.56
124	H–N(3)	Me–C_γ_(6)	-	0.62	0.47	0.47	0.47	0.47	0.49	0.58	0.47
125	H_Re_–C_γ_(3)	H–C_ß_(6)	-	0.29	0.28	0.27	0.28	0.28	0.38	*0.44*	0.28
126	H_Si_–C_γ_(3)	H–C_ß_(6)	-	0.32	0.32	0.32	0.32	0.31	0.40	*0.56*	0.30
127	H–N(3)	H_Si_–C_α_(6)	-	0.45	0.52	0.53	0.52	0.51	*0.64*	*0.70*	0.53
128	H_Re_–C_α_(4)	H–C_ß_(7)	-	0.24	0.32	0.31	0.32	0.33	*0.62*	*0.83*	0.31
129	Me–C_γ_(4)	H–C_ß_(7)	-	0.45	0.37	0.37	0.37	0.37	*0.56*	*0.66*	0.38
130	Me–C_γ_(4)	Me–C_ε1_(7)	-	0.65	0.41	0.40	0.41	0.41	0.54	0.61	0.52
131	H–N(5)	H–C_ß_(7)	-	0.36	0.44	0.43	0.44	0.45	*0.71*	*0.79*	0.37
*Nviol42*					0	0	0	0	6	9	0
*RMSD42*					0.01	0.01	0.01	0.01	0.06	0.14	0.02
*Nviol119*					3	3	3	3	14	22	0
RMSD119					0.03	0.03	0.03	0.03	0.07	0.13	0.03

a *MDsol*: MD simulation of the peptide solvated in methanol without any restraining of the molecule (*K*^RDC,*msy*^ = 0 kJ mol^–1^ Hz^–2^ using the *HRS* method). *HRSrMDsol*: RDC-restraining MD simulations with *K*^RDC,*msy*^ = 0.05 kJ mol^–1^ Hz^–2^ and . The mfv parameters of the HRS method have the values *γ^mfv^* = 2.4 ps^–1^, *K*^RDC,*mfv*^ = 100 kJ mol^–1^ Hz^–2^,and *N_mfv_* = 100. *ATrMDsol*: RDC-restraining MD simulations of the peptide solvated in methanol using the alignment-tensor (*AT*) formalism with and for different values of *K*^RDC,*AT*^. *ATrEMvac*: Energy-minimised (simulated annealing), RDC-, torsional-angle- and NOE-distance-restrained structure of the peptide in vacuo from Ref (24). *NOE distance upper bound*s: 42 from Table 1 of Ref (45) and 119 (excluding five NOE bounds involving an “HT” atom) from Table S2 of Ref (24). *RMSD42*: root-mean-square deviations of the *r*^–6^ averaged distances beyond the 42 NOE upper bounds of Ref (45). *Nviol42:* number of distance-bound violations larger than 0.1 nm. *RMSD119*: root-mean-square deviations of the *r*^*–*6^ averaged distances beyond the 119 NOE upper bounds of Ref (24). *Nviol119*: number of distance-bound violations larger than 0.1 nm. The residue sequence numbers of the atoms are within parentheses. Values 0.1 nm larger than the largest experimentally derived NOE upper bound value are in italics. “Me”: three H-atoms of a methyl group. The three hydrogens of the methyl group attached to the C_α_(4)-atom, denoted as Me-C_α_(4) in Ref (45), and as Hδ*(4) in Ref (24), are denoted as Me-C_δ_(4).

1. A set of six ^15^N–^1^H^N^ RDCs, Table 1 of ref (24), with values ranging from +3.4 Hz to +5.6 Hz. The value of *D*_*k*_^*c*^(^15^N–^1^H)= +24.36 kHz (with *r*^0^ = 0.1 nm^30^).

2. A set of seven ^13^C^ß^–^1^H^ß^ RDCs, Table 1 of ref (24), with values ranging from −17.1 Hz to −6.9 Hz. The value of *D*_*k*_^c^(^13^C–^1^H)= −46.66 kHz (with *r*^0^ = 0.109 nm^30^).

3. A set of five ^13^C^α^–^1^H^αRe^ RDCs, Table 1 of ref (24), with values ranging from −12.5 Hz to −6.2 Hz. The value of *D_k_*^*c*^(^13^C–^1^H)= −46.66 kHz (with *r*^0^ = 0.109 nm^30^).

4. A set of five ^13^C^α^–^1^H^αSi^ RDCs, Table 1 of ref (24), with values ranging from +1.5 Hz to +10.1 Hz. The value of *D*_*k*_^*c*^(^13^C–^1^H)= −46.66 kHz (with *r*^0^ = 0.109 nm^30^).

5. A set of six ^1^H^αRe^–^1^H^αSi^ RDCs, Table 1 of ref (24), with values ranging from −2.0 Hz to +2.4 Hz. The value of *D*_*k*_^*c*^(^1^H–^1^H)= −42.60 kHz (with *r*^0^ = 0.178 nm). The H^αRe^–H^αSi^ distance is not constant due to bond-angle fluctuations, but these are small and the average H^αRe^–H^αSi^ distance is *r*^0^ = 0.178 nm.

6. A set of ten ^13^C^γ^–^1^H^γ^, ^13^C^γ^–^1^H^γRe^, ^13^C^γ^–^1^H^γSi^, ^13^C^δ^–^1^H^δ^, and ^1^H^γRe^–^1^H^γSi^ RDCs in side chains, Table 2 of ref (24), with values ranging from −4.1 Hz to +6.0 Hz. For , the values mentioned above were used.

These six sets were combined into one set of *N*_RDC_ = 39 target -values for RDC-restraining.

The RDC-restraining was performed using the algorithm of rotational sampling of the magnetic field (*HRS*), described above, and applying the standard alignment-tensor approach (*AT*). Since the bonds in the molecule were kept fixed, and mainly one-bond RDCs are considered (apart from the ^1^H^αRe^–^1^H^αSi^ RDCs), no averaging of the RDC distances  was applied. Only the RDC angles (*θ*_*k*_ in eq 14) were subject to restraining. The experimental accuracy of the measured RDCs was estimated as 0.5–1.8 Hz.^24^ The flat-bottom parameter of the restraining function was set to *ΔD*^*fb*^ = 2.0 Hz, a slightly larger value to allow for unknown uncertainty. This implies a width of 4 Hz of the flat bottom of the restraining potential-energy function (eq 9a). The parameter *ΔD*^*h*^ restricting the range of the harmonic part of the restraining function beyond which this function becomes linear was set equal to 1 Hz. The force constants *K*^RDC,*mfv*^ and *K*^RDC,*msy*^, the number *N*_*mfv*_ of SD steps of the magnetic-field vector simulations, the memory relaxation times  and , and the number *N*_*RDC*_ of RDCs used in the restraining were varied.

The *HRS* algorithm can be applied in two different ways: (i) Matching <*D*_*k*_> with  by sampling and RDC-restraining directions of the magnetic-field vector (*K*^RDC,*mfv*^ > 0), but without RDC restraining in the *msy* part of the simulation (*K*^RDC,*msy*^ = 0). In this case, RDC restraining does not induce conformational changes in the molecule. So the *HRS* algorithm allows the determination of RDC values for trajectory structures in a simulation, in which the RDC restraints do not affect the molecular configurations, while an orientation distribution of the magnetic-field vector (equivalent to an orientation distribution of the molecule) is generated that minimizes the deviations of <*D*_*k*_>-values with respect to a given set of  target RDC values. If a further reduction of |<*D*_*k*_>−| is needed, this may be achieved by the RDC-restraining in the *msy* part of the simulation of the molecular system at each SD or MD time step (*K*^RDC,*msy*^ > 0) with a restraining force on the molecule proportional to . (ii) Matching <*D*_*k*_> with  by alternating between the sampling of directions of the magnetic-field vector (*K*^RDC,*mfv*^ > 0) for *N*_*mfv*_ time steps and the sampling of the motions of the molecule in the presence of RDC straining for *N*_*msy*_ time steps (*K*^*RDC,msy*^ > 0). In this case, RDC-restraining-induced conformational changes in the molecule may occur.

### 4.6 Structure Determination and Simulations Performed

Different types of SD or MD simulations of the ß-heptapeptide applying different sets of RDC restraints were performed, resulting in trajectories or ensembles of solute structures.

1. MD simulation of the solute solvated in methanol in a periodic box (*MDsol*) using the GROMOS 54A7 force field without RDC-restraining of the molecule (*K*^RDC,*msy*^ = 0 kJ mol^–1^ Hz^–2^) in order to calculate RDC values (*K*^RDC,*mfv*^ > 0 kJ mol^–1^ Hz^–2^, when using the *HRS* method), NOE atom–atom distances, and ^3^*J*-couplings without any restraining of the molecule. The average temperatures of solute and solvent are 299 K.
2. RDC-restraining MD simulations of the solute solvated in methanol in a periodic box (*HRSrMDsol*) using the GROMOS 54A7 force field and the rotational sampling of the magnetic-field RDC-restraining *HRS* method with *K*^RDC,*msy*^ > 0 kJ mol^–1^ Hz^–2^. The average temperatures of solute and solvent are 299 K.
3. RDC-restraining MD simulations of the solute solvated in methanol in a periodic box (*ATrMDsol*) using the GROMOS 54A7 force field and the standard alignment-tensor RDC-restraining *AT* method ( =  = 0).^34^ The average temperatures of solute and solvent are 299 K, except for *K*^RDC,*AT*^ = 10 kJ mol^–1^ Hz^–2^ with an average solvent temperature of 322 K.
4. SD simulation of the solute in vacuo (*SDvac*) using the GROMOS 54B7 force field without RDC restraining of the molecule (*K*^RDC,*msy*^ = 0 kJ mol^–1^ Hz^–2^) in order to calculate RDC values (*K*^RDC,*mfv*^ > 0 kJ mol^–1^ Hz^–2^, when using the *HRS* method), NOE atom–atom distances, and ^3^*J*-couplings without any restraining of the molecule. The average temperature is 299 K.
5. RDC-restraining SD simulations of the solute in vacuo (*HRSrSDvac*) using the GROMOS 54B7 force field and the rotational sampling of the magnetic-field RDC-restraining *HRS* method (with *K*^RDC,*msy*^ > 0 kJ mol^–1^ Hz^–2^). The average temperature is 299 K.
6. RDC-restraining SD simulations of the solute in vacuo (*ATrSDvac*) using the GROMOS 54B7 force field and the standard alignment-tensor RDC-restraining *AT* method ().^34^ The average temperature is 299 K, except for *K*^RDC,*AT*^ = 10 kJ mol^–1^ Hz^–2^ with an average temperature of 318 K.

The MD and SD simulations were *t*^*msy*^ = *t*^*AT*^ = 100 ns long.

### 4.7 Analysis of Atomic Trajectories and RDC Values

**Figure 2:**
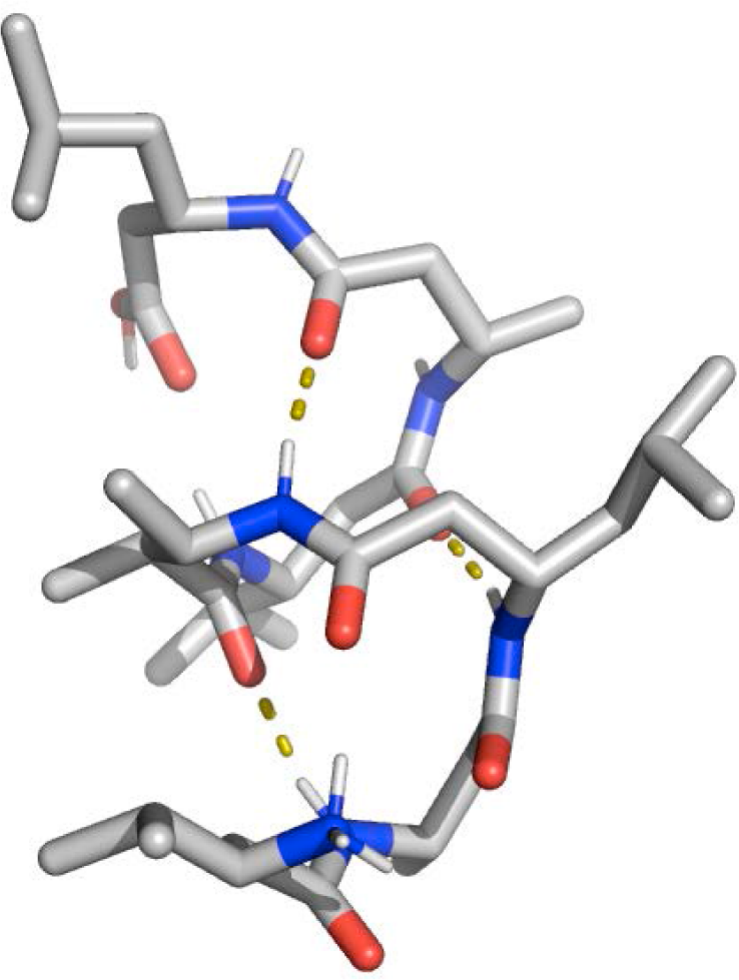
Initial structure of the left-handed *M*-3_14_-helical fold of the ß-heptapeptide H_2_^+^-ß^3^-HVal-ß^3^-HAla-ß^3^-HLeu-(S,S)-ß^3^-HAla(αMe)-ß^3^-HVal-ß^3^-HAla-ß^3^-HLeu-OH.^48^ Dashed lines indicate the three backbone hydrogen bonds of the left-handed *M*-3_14_-helical fold (see Table 3).

Atomic coordinates were stored at 2 ps intervals and energies at 0.2 ps intervals for analysis.^41^ The distribution of the *θ*_*ab,H*_-angle between an intramolecular vector  connecting two atoms *a* and *b* in the molecule and the vector representing the direction of the magnetic field  was calculated from the trajectories in order to analyze the anisotropy of the rotational distributions of the ß-heptapeptide in the (RDC-restraining) simulations. No magic-angle peaks should appear in the distribution of the angle  or in the distribution of the angle  between the RDC vector for and the magnetic-field direction . They would indicate improper rotational sampling. For the ß-heptapeptide, four different vectors  were chosen: The vector connecting atoms C_α_(1) and C_α_(7), which lies more or less in the direction of the *M*-3_14_-helix, and the three more or less orthogonal vectors C_α_(3)–C(3), C_α_(4)–C(4), and C_α_(5)–C(5) in the middle of the helix, see Figure 2.

In the present case of RDC restraining, using a flat-bottom of  ± 2 Hz in the restraining potential energy term, deviations |<*D*_*k*_>−| ≤ 2 Hz are considered insignificant.

The GROMOS force fields treat aliphatic carbons as united CH, CH_2_, and CH_3_ atoms. So when calculating NOE distances, interhydrogen distances involving the aliphatic hydrogen atoms were calculated using virtual atomic positions for CH and pro-chiral CH_2_^27,31,32^ and pseudoatomic positions for CH_3_^60^ for those hydrogen atoms,^32^ if applicable. Two sets of NOE distance upper bounds for the ß-heptapeptide are available. A set of 42 NOE upper bounds (*NOE42*)^25,45^ (Table 1 of ref (45) and a set of 119 NOE upper bounds (*NOE119*)^24^ (Table S2 of Supporting Information of ref (24)), see Table 1. A pseudoatom NOE distance bound correction of 0.1 nm for CH_3_ groups was applied to the upper bounds of ref (45) and to the upper bounds of ref (24). The two sets have 30 NOE bounds in common, of which only four have an identical upper bound value; see Table 1. The interhydrogen distances were calculated as <*r*^–6^>^–1/6^, i.e., using *r*^–6^ averaging over the trajectory structures,^45,48^ where *r* indicates the actual hydrogen–hydrogen distance.

In view of the uncertainty inherent to the calculation of the NOE bounds and *r*^–6^ averaged distances, the NOE distance upper bound violations of less than 0.1 nm are considered insignificant.

The set of 21 ^3^*J*_*HN-HC*_ couplings and ^3^*J*_*HC-HC*_ couplings for the ß-heptapeptide^24,25,45^ can be found in Table 2, together with the values obtained from the various simulations. For the calculation of the ^3^*J*_*HN-HC*_ couplings, the Karplus relation^61,62^ was used with the parameter values *a* = 6.4 Hz, *b* = −1.4 Hz, and *c* = 1.9 Hz^63^ for the ^3^*J*_*HN-HC*_ couplings. The ^3^*J*_*HC-HC*_ couplings were calculated using the parameter values *a* = 9.5 Hz, *b* = −1.6 Hz, and *c* = 1.8 Hz.^64^

**Table 2:** List of 21 ^3^*J*-Coupling Constants (Hz) Derived from Experiment and Averaged from Unrestrained and RDC-Restrained (*ΔD*^*fb*^ = 2.0 Hz) *t*^*msy*^ = *t*^*AT*^ = 100 ns MD Simulations of the ß-Heptapeptide^a^

^3^*J*-coupling sequence number	^3^*J*-coupling dihedral angle	Exp. value (Hz)		^3^*J*-coupling (Hz)						
*Simulation*		Ref (45)	Ref (24)	*MDsol*	*HRSrMDsol*	*ATrMDsol*				*ATrEMvac*
*K*RDC,*AT*	kJ mol^– 1^Hz^–2^					0	0.1	1	10	
1	H_ß_(1)–C_ß_(1)–C_α_(1)–H_αSi_(1)	2.8		2.6	2.7	2.6	2.7	3.3	3.9	*5.2*
2	H_ß_(2)–C_ß_(2)–C_α_(2)–H_αSi_(2)	4.5	4.4	3.7	3.8	3.7	3.8	5.4	6.1	2.8
3	H_ß_(3)–C_ß_(3)–C_α_(3)–H_αSi_(3)	4.5	4.2	3.9	4.0	3.9	4.0	3.9	4.4	3.7
4	H_ß_(5)–C_ß_(5)–C_α_(5)–H_αSi_(5)	3.9	3.7	3.4	3.4	3.3	3.4	3.4	3.9	3.4
5	H_ß_(6)–C_ß_(6)–C_α_(6)–H_αSi_(6)	3.8	4.1	3.3	3.2	3.3	3.1	5.4	*6.7*	2.5
6	H_ß_(7)–C_ß_(7)–C_α_(7)–H_αSi_(7)	4.5		4.1	4.1	4.1	4.2	4.6	4.6	*1.8*
7	H_ß_(1)–C_ß_(1)–C_γ_(1)–H_γ_(1)	4.7	5.1	5.0	4.4	5.0	5.0	5.0	4.5	*12.8*
8	H_ß_(5)–C_ß_(5)–C_γ_(5)–H_γ_(5)	7.0	7.0	6.4	6.2	6.4	5.6	6.4	6.2	*2.5*
9	H_N_(2)–N(2)–C_ß_(2)–H_ß_(2)	9.2	9.1	9.2	9.2	9.2	9.2	8.8	8.5	8.3
10	H_N_(3)–N(3)–C_ß_(3)–H_ß_(3)	9.6	9.2	9.1	9.1	9.1	9.1	8.8	8.0	*6.5*
11	H_N_(4)–N(4)–C_ß_(4)–H_ß_(4)	9.3	9.0	9.3	9.3	9.3	9.3	8.8	8.5	7.0
12	H_N_(5)–N(5)–C_ß_(5)–H_ß_(5)	9.6	9.4	9.3	9.3	9.3	9.3	9.2	8.8	9.7
13	H_N_(6)–N(6)–C_ß_(6)–H_ß_(6)	8.7	8.6	9.2	9.2	9.2	9.2	8.5	8.0	9.7
14	H_N_(7)–N(7)–C_ß_(7)–H_ß_(7)	9.5	9.3	9.2	9.2	9.2	9.2	8.9	8.7	8.0
15	H_ß_(1)–C_ß_(1)–C_α_(1)–H_αRe_(1)	11.5	11.7	11.9	11.8	11.9	11.8	10.7	10.2	12.3
16	H_ß_(2)–C_ß_(2)–C_α_(2)–H_αRe_(2)	12.0	12.1	12.6	12.3	12.6	12.5	*9.1*	*7.7*	12.8
17	H_ß_(3)–C_ß_(3)–C_α_(3)–H_αRe_(3)	12.3		12.5	12.6	12.5	12.6	10.8	*9.5*	12.9
18	H_ß_(4)–C_ß_(4)–C_α_(4)–H_αRe_(4)	10.8	10.9	12.4	12.4	12.4	12.2	*5.4*	*3.6*	11.7
19	H_ß_(5)–C_ß_(5)–C_α_(5)–H_αRe_(5)	12.3	12.2	12.5	12.6	12.5	12.5	11.6	10.7	12.9
20	H_ß_(6)–C_ß_(6)–C_α_(6)–H_αRe_(6)	11.6	11.4	11.4	11.6	11.4	11.2	*8.7*	*6.5*	12.7
21	H_ß_(7)–C_ß_(7)–C_α_(7)–H_αRe_(7)	10.0		9.4	9.6	9.4	9.4	9.8	9.7	11.6
*Ndev*				0	0	0	0	3	5	5
*RMSD*				0.5	0.5	0.5	0.6	1.6	*2.5*	*2.5*

a *MDsol*: MD simulation of the peptide solvated in methanol without any restraining of the molecule (*K*^RDC,*msy*^ = 0 kJ mol^–1^ Hz^–2^ using the *HRS* method). *HRSrMDsol*: RDC-restraining MD simulations with *K*^RDC,*msy*^ = 0.05 kJ mol^–1^ Hz^–2^ and . The *mfv* parameters of the *HRS* method have the values *γ^mfv^* = 2.4 ps^–1^, *K*^RDC,*mfv*^ = 100 kJ mol^–1^ Hz^–2^,and *N_mf__v_* = 100. *ATrMDsol*: RDC-restraining MD simulations of the peptide solvated in methanol using the alignment-tensor (*AT*) formalism with  and for different values of *K*^RDC,*AT*^. *ATrEMvac*: Energy-minimised (simulated annealing), RDC-, torsional-angle- and NOE-distance-restrained structure of the peptide in vacuo from ref (24). *Ndev*: number of deviations between simulation and experiment larger than 2 Hz. *RMSD*: root-mean-square difference between experiment (ref (45)) and simulation for the 21 ^3^*J*-coupling constants (Hz). The residue sequence numbers of the atoms are within parentheses. Experimentally derived ^3^*J*-couplings from Table 2 of ref (45) and from Tables 2 and 3 of ref (24). MD values differing more than 2 Hz from the experimental value are in italics.

In view of the various factors contributing to an uncertainty of about 2 Hz inherent to the Karplus relation linking structure and ^*3*^*J*-couplings,^65^ a deviation of less than 2 Hz between ^3^*J*-coupling values calculated from the simulations and ^3^*J*-coupling values derived from the experiment is considered insignificant.

Hydrogen bonds were identified according to a geometric criterion: a hydrogen bond was assumed to exist if the hydrogen-acceptor distance was less than 0.25 nm, and the donor-hydrogen-acceptor angle was larger than 135°.

### 4.8 Criteria to Test the Performance of the Rotational-Sampling Algorithm

The following criteria to test the performance of the algorithm were used:^23^

1. Individual |<*D*_*k*_>−| values or *RMSD*(<*D*>*–D*^0^) values should be lower than 2 Hz.
2. No magic-angle peaks should appear in the distribution of the angle  between the intramolecular vector connecting atoms *a* and *b* (vectors C_α_(1)–C_α_(7), C_α_(3)–C(3), C_α_(4)–C(4), and C_α_(5)–C(5)) in the molecule and the magnetic-field direction , or in the distribution of the angle  between the RDC-vector for and the magnetic-field direction .
3. If the nonzero target  values belong to a particular orientation distribution of the molecule, either computer generated in a molecular simulation (not postulated) or measured without errors, the distribution of an angle  or an angle  (i.e., ) should not be completely flat, because the <*D*_*k*_>_*t*_ corresponding to a flat  distribution would be zero.
4. RDC-restraining forces *f*^*mfv*^ and *f*^*msy*^ should be neither too small (no effect of restraining) nor too big compared to the forces due to the physical force field. No SHAKE failures, which indicate unphysically large (restraining) forces, should occur.^23^
5. No large intramolecular atom-positional or torsional-angle fluctuations of the molecule compared to the fluctuations allowed by the force field used should occur. Large fluctuations may be induced by the RDC-restraining algorithm and thus be an artifact.^23^

## 5 Results

### 5.1 Exploring the Parameter Ranges of the *HRS* Algorithm

The root-mean-square differences (*RMSD*) between the 39 RDC values  calculated from the MD simulations of the β-heptapeptide solvated in methanol (*MDsol*) and the 39 target -values derived from the experiment are shown in Table S1. The table shows results for more than 80 out of the many combinations of the five parameters, *K*^RDC,*mfv*^, , *N*_*mfv*_, , and *K*^RDC.*msy*^ of the *HRS* algorithm. The case *K*^RDC.*msy*^ = 0 represents the absence of any RDC or other restraining of the molecule; only the magnetic-field vector is RDC restrained. In this case, the *RMSD* values range from 0.5 to 11.2 Hz, while when applying RDC restraining also to the molecule (*K*^RDC.*msy*^ > 0), the *RMSD* values range from 0.4 to 10.6 Hz.

Using the low *mfv* force constant *K*^RDC.*mfv*^ = 1 kJ mol^–1^ Hz^–2^, all *RMSD* values are larger than 2 Hz. The *mfv* RDC-restraining forces on the magnetic-field vector are too weak, see Table S2, to generate an orientation distribution of the magnetic-field vector that produces RDC values close (<2 Hz) to the target ones, and the *msy* RDC-restraining forces on the molecule are not strong enough to drive the difference between calculated and target RDC values below 2 Hz. Using a larger *mfv* force constant *K*^RDC.*mfv*^ = 10 kJ mol^–1^ Hz^–2^, this effect is still observed using a larger -value of 100 ns and *N*_*mfv*_ = 10. The *RMSD* values in Table S1 and the RDC-restraining forces in Table S2 reflect the fact that the *mfv* and *msy* RDC-restraining forces are proportional to *K*^RDC.*mfv*^/ and *K*^RDC.*msy*^/, respectively.

For *K*^RDC,*mfv*^ = 100 or 1000 kJ mol^–1^ Hz^–2^, the lowest root-mean-square differences RMSD(<*D*_*k*_>*–**)* are found, 0.4 to 1.5 Hz. However, the orientation distributions generated using these *mfv* force-constant values, shown in Figures S1 and S2 for 10 RDCs, display magic-angle peaks, i.e., sampling artifacts for *K*^RDC.*mfv*^/ > 1 kJ mol^–1^ Hz^–2^ ns^–1^. The occurrence of oversampling around the magic angles can be quantified by calculating the ratio of sampling angles, between magnetic field vector and different vectors in the molecule, around the magic angles (55°,125°) and angles around 90°; see Table S3. Ratios larger than 0.9 hint at oversampling around the magic angles. As was observed when applying the *HRS* algorithm to a cyclo-octane molecule,^23^ magic-angle peaks emerge when the RDC-restraining forces *f*^*mfv*^ on the magnetic-field vector become too large, much larger than about 10 kJ mol^–1^ nm^–1^, see Table S2. In view of these observations, the *mfv* parameter value combinations involving *K*^RDC,*mfv*^ = 1 and 1000 kJ mol^–1^ Hz^–2^, the combination of *K*^RDC,*mfv*^ = 100 kJ mol^–1^ Hz^–2^ with , and the combination *K*^RDC,*mfv*^ = 10 kJ mol^–1^ Hz^–2^ with  and *N*_*mfv*_ = 10 will not be considered further. The *msy* parameter values *K*^RDC,*msy*^ = 0.05 kJ mol^–1^ Hz^–2^ with  will be investigated in further detail. In the *mfv* simulation part of the algorithm, the values of *N*_*mfv*_ and  should be sufficiently large to reduce the < *D*_*k*_> values from the kHz to the Hz range by sampling the orientation distribution of the magnetic-field vector sufficiently. In the *msy* simulation part of the algorithm, the value of  controls the (additional) averaging of the *<D*_*k*_> values over the intramolecular motion. This means that the value of  may be chosen (much) shorter than the value of . It also means that large *msy* RDC-restraining forces, see Table S4, do not necessarily lead to magic-angle peaks and so would be allowed.

Table S5 shows that by applying the *mfv* parameter values *K*^RDC,*mfv*^ = 100 kJ mol^–1^ Hz^–2^,  and *N*_*mfv*_ = 100, all RDC values calculated using the *HRS* algorithm are within 1.8 Hz from the experimentally derived target RDC values, with an *RMSD*( <*D*_*k*_>-) for the 39 RDCs of only 0.6 Hz.

These *mfv* parameter combinations, without RDC restraining of the molecule (*K*^RDC,*msy*^ = 0) or with (*K*^RDC,*msy*^ = 0.05 kJ mol^–1^ Hz^–2^), also show the lowest violations of NOE atom–atom distance upper bounds (Table S6) and deviations from measured ^3^*J*-coupling values (Table S7). In addition, they show no loss of the helical fold in terms of backbone hydrogen bonds (Tables S8 and S9). Some other parameter combinations do lead to a loss of backbone hydrogen bonding, while retaining an almost equally good agreement with RDC target values (*K*^RDC,*mfv*^ = 100 kJ mol^–1^ Hz^–2^, , *N*_*mfv*_ = 10, *K*^RDC,*msy*^ = 0.05 kJ mol^–1^ Hz^–2^ and ).

### 5.2 Calculated NMR Observables from Unrestrained MD Simulations in Methanol Solution (*MDsol*)

The simulation without any restraining of the molecule shows rather minor violations of the NOE atom–atom distance upper bounds, see Table 1. For the set of 42 NOE distance upper bounds of ref (45), there are only 8 violations with an average value of 0.01 nm and a largest violation of 0.05 nm for NOE No 31, no violations larger than 0.1 nm. For the set of 119 NOE distance upper bounds of ref (24), there are 28 violations with an average value of 0.03 nm and a largest violation of 0.21 nm for NOE No 106, only three violations larger than 0.1 nm: No 106 (0.21 nm), H–C_β_(2)–H–C_δ_(3), No 111 (0.13 nm), H–N(4)–Me–C_δ1_(5) and No 115 (0.12 nm), H–N(6)–H–C_β_(7). Why these three bounds are not satisfied remains to be investigated (Section 5.10). This could be due to a particular feature of the (GROMOS) force field used or the bound derived from experiment (ref (24)) may be too tight.

The root-mean-square difference between 21 measured and simulated ^3^*J*-couplings is only 0.5 Hz, see Table 2. The simulation of the unrestrained molecule (*MDsol*) matches all experimentally derived ^3^*J*-coupling values.

Solvated in pure methanol, the folding equilibrium of the β-heptapeptide at 300 K is dominated by a left-handed *M*-3_14_-helical folded conformation, characterized by three hydrogen bonds in the backbone (see Figure 2). Without any restraining (*MDsol*), the percentage of helical hydrogen bonding is 86% (Table S9). Table 3 shows the percentages for the three individual hydrogen bonds: 87%, 88%, and 81%.

**Table 3:** Intramolecular M-3_14_-Helical Hydrogen Bonds (%) from Unrestrained and RDC-Restrained (*ΔD*^*fb*^ = 2.0 Hz) *t*^*msy*^ = *t*^*AT*^ = 100 ns MD Simulations of the ß-Heptapeptide^a^

Hydrogen-bond sequence number	Donor and acceptor atoms		% Hydrogen-bonding						
*Simulation*			*MDsol*	*HRSrMDsol*	*ATrMDsol*				*ATrEMvac*
*K*RDC,AT	kJ mol^–1^ Hz^–2^				0	0.1	1	10	
1	NH1(1)	O(3)	8	7	8	7	1	0	0
2	NH2(1)	O(3)	8	7	8	7	1	0	0
3	NH(2)	O(4)	87	89	87	90	13	0	100
4	NH(3)	O(5)	88	98	88	95	13	1	100
5	NH(4)	O(6)	81	90	81	80	3	0	0

a *MDsol: MD* simulation of the peptide solvated in methanol without any restraining of the molecule (*K*^RDC,*msy*^ = 0 kJ mol^–1^ Hz^–2^ using the HRS method). *HRSrMDsol*: RDC-restraining MD simulations with *K*^RDC,*msy*^ = 0.05 kJ mol^–1^ Hz^–2^ and . The *mfv* parameters of the HRS method have the values *γ^mfv^* = 2.4 ps^–1^, *K*^RDC,*mfv*^ = 100 kJ mol^–1^ Hz^–2^,  and *Nmfv* = 100. *ATrMDsol*: RDC-restraining MD simulations of the peptide solvated in methanol using the alignment-tensor (*AT*) formalism with  and for different values of *K*^RDC,*AT*^. *ATrEMvac*: Energy-minimised (simulated annealing), RDC-, torsional-angle- and NOE-distance-restrained structure of the peptide in vacuo from ref (24). The residue sequence numbers of the atoms are within parentheses.

### 5.3 Recovering RDCs Using RDC Restraining of the Molecule (*HRSrMDsol*) in Methanol Solution

The rotational sampling of the magnetic-field vector produces a nonuniform rotational distribution of the magnetic-field vector, which is equivalent to a nonuniform rotational distribution of the molecule. Applying in addition RDC-restraining to the molecule, that is, the *msy* part of the algorithm, does not improve the agreement between the simulated and target RDC values (Tables 4 and S5). The best *RMSD*(<*D*_*k*_>-) value reached for *K*^RDC,*msy*^ > 0 is 0.6 Hz (*K*^RDC,*msy*^ = 0.05 kJ mol^–1^ Hz^–2^ using the *mfv* parameter values *K*^RDC,*mfv*^ = 100 kJ mol^–1^ Hz^–2^,  and *N*_*mfv*_ = 100). The relatively stable helical structure of the molecule in methanol does not seem to require significant structural changes to match the experimental RDC values.

**Table 4:** List of 39 RDC-Values (Hz) derived from Experiment, , and the Averages <*D*_*k*_> from Unrestrained and RDC-Restrained (*ΔD*^*fb*^ = 2.0 Hz) *t*^*msy*^ = *t*^*AT*^ = 100 ns MD Simulations of the ß-Heptapeptide^a^

RDC sequence number	RDC atoms		(Hz)	(Hz)						(Hz)
*Simulation*				*MDsol*	*HRSrMDsol*	*ATrMDsol*				*ATrEMvac*
*K*RDC,AT	kJ mol^–1^ Hz^–2^					0	0.1	1	10	
1	^1^H(2)	^15^N(2)	3.4	3.5	3.7	2.7	2.7	*0.7*	*0.0*	2.3
2	^1^H(3)	^15^N(3)	5.6	5.3	5.3	*3.2*	*3.3*	*0.6*	*–0.1*	4.0
3	^1^H(4)	^15^N(4)	4.4	4.5	4.8	3.4	3.4	*0.5*	*–0.1*	3.0
4	^1^H(5)	^15^N(5)	4.5	4.3	4.5	2.5	2.6	*1.0*	*0.0*	3.0
5	^1^H(6)	^15^N(6)	5.2	4.9	5.0	*2.9*	*2.9*	*0.1*	*–0.2*	3.8
6	^1^H(7)	^15^N(7)	4.7	4.6	4.1	*2.3*	*2.1*	*0.2*	*–0.1*	3.4
7	^1^H_ß_(1)	^13^C_ß_(1)	–6.9	–6.5	–6.5	*-4.2*	*–3.9*	*–1.6*	*–0.4*	–6.2
8	^1^H_ß_(2)	^13^C_ß_(2)	–12.3	–12.3	–12.2	*-8.1*	*–8.2*	*–2.5*	*–0.3*	*–9.6*
9	^1^H_ß_(3)	^13^C_ß_(3)	–17.1	–15.3	–15.4	–9.2	–9.5	–2.6	*–0.4*	*–11.7*
10	^1^H_ß_(4)	^13^C_ß_(4)	–12.0	–12.1	–11.9	*-8.9*	*–9.0*	*–1.7*	*–0.0*	–10.2
11	^1^H_ß_(5)	^13^C_ß_(5)	–11.3	–11.1	–11.2	*-7.9*	*–8.0*	*–3.2*	*–0.4*	–10.0
12	^1^H_ß_(6)	^13^C_ß_(6)	–12.6	–12.1	–12.6	*-8.6*	*–8.7*	*–1.6*	*–0.1*	*–9.9*
13	^1^H_ß_(7)	^13^C_ß_(7)	–12.8	–11.7	–12.0	*-6.3*	*–5.9*	*–1.2*	*–0.3*	*–9.5*
14	^1^H_α*Re*_(1)	^13^C_α_(1)	–6.2	–6.4	–6.4	*-4.0*	*–3.8*	*–1.3*	*–0.4*	–6.0
15	^1^H_α*Re*_(2)	^13^C_α_(2)	–11.0	–10.6	–11.0	*-8.2*	*–8.3*	*–2.9*	*–0.6*	*–8.6*
16	^1^H_α*Re*_(4)	^13^C_α_(4)	–12.5	–12.5	–12.3	*–9.1*	*–9.2*	*–3.1*	*–0.5*	*–9.1*
17	^1^H_α*Re*_(6)	^13^C_α_(6)	–8.6	–9.7	–9.6	–7.0	–6.9	*–1.7*	*–0.4*	–10.2
18	^1^H_α*Re*_(7)	^13^C_α_(7)	–8.7	–8.5	–8.3	*–4.4*	*–4.1*	*–1.3*	*–0.4*	–7.3
19	^1^H_α*Si*_(1)	^13^C_α_(1)	1.5	1.4	1.3	0.7	0.8	0.1	0.1	*4.8*
20	^1^H_α*Si*_(2)	^13^C_α_(2)	6.9	7.1	7.0	6.5	6.0	*0.9*	*0.3*	5.2
21	^1^H_α*Si*_(5)	^13^C_α_(5)	10.1	9.7	9.5	*6.0*	*6.4*	*0.5*	*0.3*	*6.8*
22	^1^H_α*Si*_(6)	^13^C_α_(6)	7.1	6.1	6.1	*3.8*	*3.4*	*0.7*	*0.1*	5.8
23	^1^H_α*Si*_(7)	^13^C_α_(7)	2.2	2.4	2.6	2.3	2.6	1.1	0.2	3.5
24	^1^H_α*Re*_(1)	^1^H_α*Si*_(1)	–1.5	–1.2	–1.1	0.1	0.3	–0.2	–0.1	*0.9*
25	^1^H_α*Re*_(2)	^1^H_α*Si*_(2)	1.1	0.6	0.6	0.3	0.1	–0.8	–0.1	1.3
26	^1^H_α*Re*_(3)	^1^H_α*Si*_(3)	–0.9	–1.4	–1.3	–2.0	–1.7	–0.6	0.1	*–4.3*
27	^1^H_α*Re*_(5)	^1^H_α*Si*_(5)	2.4	2.5	2.9	1.4	1.8	*–0.6*	*0.1*	1.2
28	^1^H_α*Re*_(6)	^1^H_α*Si*_(6)	–0.2	0.7	1.0	0.3	0.3	–0.2	–0.1	*–2.5*
29	^1^H_α*Re*_(7)	^1^H_α*Si*_(7)	–2.0	–2.1	–2.3	–0.9	–0.7	–0.3	–0.1	*1.6*
30	^1^H_γ_(1)	^13^C_γ_(1)	–1.2	–1.4	–1.0	–1.6	–1.4	–0.2	–0.0	*3.6*
31	^1^H_γ*Re*_(3)	^13^C_γ_(3)	–2.9	–3.3	–3.2	*0.6*	*0.6*	*–0.3*	*–0.0*	*4.8*
32	^1^H_γSi_(3)	^13^C_γ_(3)	4.1	2.8	2.4	*–5.3*	*–5.2*	*0.0*	*0.1*	*1.6*
33	^1^H_γ*Re*_(3)	^1^H_γSi_(3)	0.8	1.1	1.4	*–3.0*	*–2.9*	0.0	0.1	*8.6*
34	^1^H_δ_(3)	^13^C_δ_(3)	6.0	5.9	5.6	*3.9*	4.2	*1.6*	*0.3*	*–5.3*
35	^1^H_γ_(5)	^13^C_γ_(5)	–4.1	–4.2	–3.9	–2.2	*–1.9*	*–0.9*	*–0.2*	*–11.4*
36	^1^H_γ*Re*_(7)	^13^C_γ_(7)	–3.6	–4.0	–3.7	–1.7	*–1.3*	*–0.3*	*–0.1*	*3.6*
37	^1^H_γSi_(7)	^13^C_γ_(7)	2.3	1.6	1.9	*–1.0*	*–0.9*	0.4	*0.0*	*–7.4*
38	^1^H_γ*Re*_(7)	^1^H_γSi_(7)	–1.4	–1.1	–1.0	–1.8	–1.7	0.0	–0.0	0.4
39	^1^H_δ_(7)	^13^C_δ_(7)	6.0	5.8	5.7	*2.7*	*2.8*	*1.1*	*0.3*	*2.3*
*RMSD*				0.6	0.6	*3.3*	*3.3*	*6.1*	*7.0*	*4.1*

a *MDsol*: MD simulation of the peptide solvated in methanol without any restraining of the molecule (*K*^RDC,*msy*^ = 0 kJ mol^–1^ Hz^–2^ using the *HRS* method). *HRSrMDsol*: RDC-restraining MD simulations with *K*^RDC,*msy*^ = 0.05 kJ mol^–1^ Hz^–2^ and . The *mfv* parameters of the *HRS* method have the values *γ^mfv^* = 2.4 ps^–1^, *K*^RDC,*mfv*^ = 100 kJ mol^–1^ Hz^–2^,  and *N_mfv_* = 100. *ATrMDsol*: RDC-restraining MD simulations of the peptide solvated in methanol using the alignment-tensor (*AT*) formalism with  and for different values of *K*^RDC,*AT*^. *ATrEMvac*: Energy-minimised (simulated annealing), RDC-, torsional-angle- and NOE-distance-restrained structure of the peptide in vacuo from ref (24). *RMSD*: root-mean-square difference between  and  for the 39 bond-vector RDCs. The residue sequence numbers of the atoms are within parentheses. RDC-values  from Tables 1 and 2 of ref (24). Deviations of averaged RDC-values  from their target values  larger than 2 Hz are in italics.

Applying RDC restraining to the molecule (*K*^RDC,*msy*^ > 0) using appropriate *mfv* parameter values (*N*_*mfv*_ > 10), that is, the *msy* part of the algorithm, also does not improve the agreement between simulated and experimentally derived NOE atom–atom distance upper bounds (Tables 1 and S6). Use of *N*_*mfv*_ = 10 leads to more violations, both for the set of 42 NOEs and for the set of 119 NOEs (Table S6).

A similar observation holds for the ^3^*J*-couplings (Tables 2 and S7). Using *N*_*mfv*_ = 10, two or four deviations of more than 2 Hz are observed.

Using the *mfv* parameter values *K*^RDC,*mfv*^ = 100 kJ mol^–1^ Hz^–2^,  and *N*_*mfv*_ = 100, the three helical hydrogen bonds are still present (Table 3). In the presence of RDC-restraining forces on the molecule, ignoring the cases mentioned before that lead to magic-angle peaks, according to Table S8, for the two *K*^RDC,*msy*^-values investigated, these hydrogen bonds are lost at  . Table S9 shows that for *N*_*mfv*_ = 10, the three hydrogen bonds are not at all dominant. They are dominant for *K*^RDC,*mfv*^ = 10 or 100 kJ mol^–1^ Hz^–2^ with  and *N*_*mfv*_ = 100 or 1000.

### 5.4 RDC Values of Unrestrained MD Simulations in Methanol Solution (*MDsol*) Using Subsets of the 39 RDC Values Derived from Experiment

**Figure 3:**
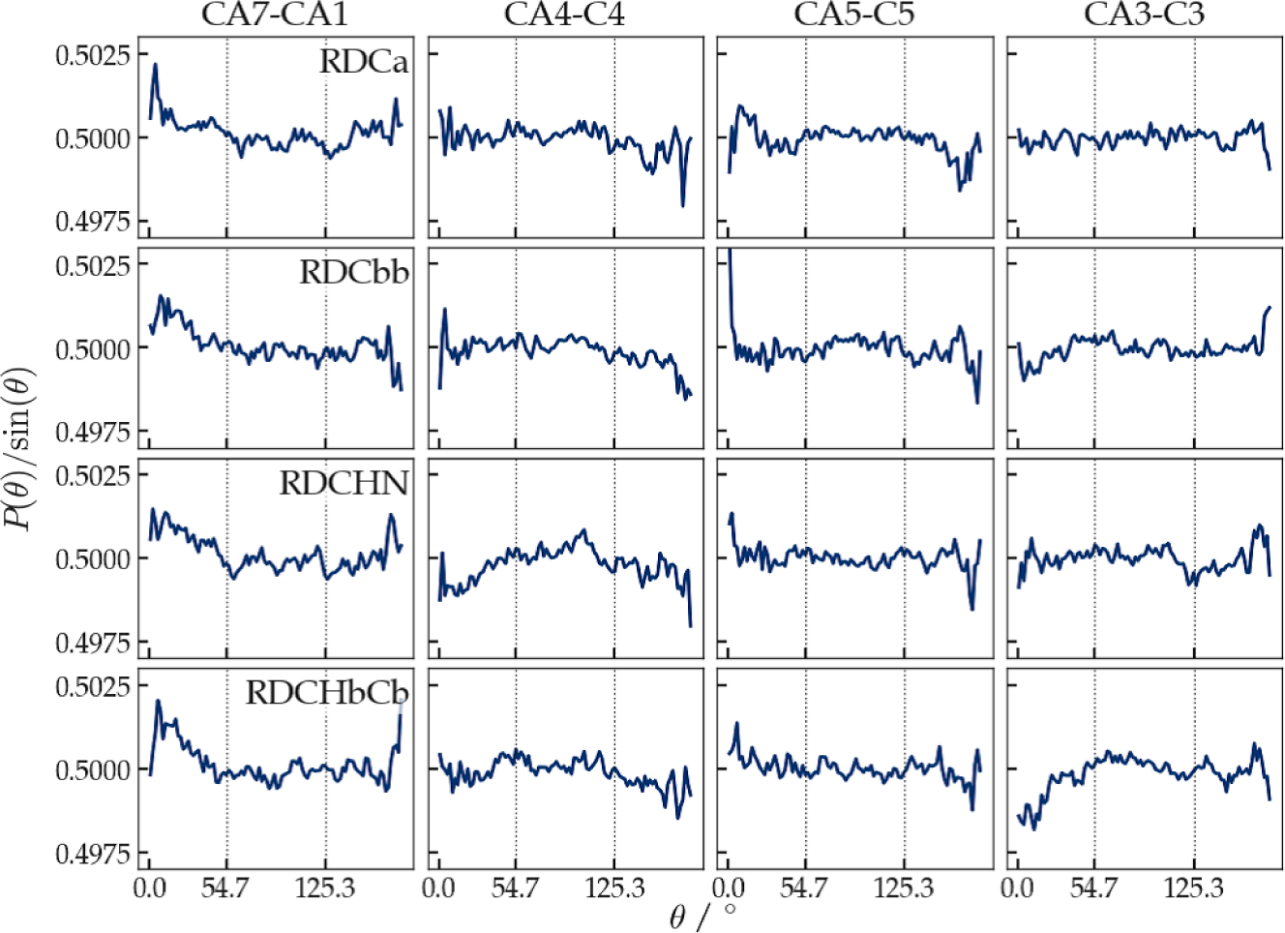
Distribution of the angle  between the four vectors , where *a* and *b* indicate the four atom pairs C_α_(1)–C_α_(7), C_α_(3)–C(3), C_α_(4)–C(4), and C_α_(5)–C(5), and the magnetic-field direction  from MD simulations of the ß-heptapeptide solvated in methanol without any restraining of the molecule (*MDsol*, *K*^RDC,*msy*^ = 0) using four different (sub)sets of RDCs (Table 5). *RDCa*: all 39 RDCs, *RDCbb*: only the 29 backbone RDCs, No 1–29. *RDCHN*: only the 6 H–N backbone RDCs, No 1–6. *RDCHBCB*: only the 7 backbone HB–CB RDCs, No 7–13. Parameter values of the *t*^*msy*^ = 100 ns *HRS* simulations are *γ*^*mfv*^ = 2.4 ps^–1^, *ΔD*^*fb*^ = 2.0 Hz, *K*^*RDC,mfv*^ = 100 kJ mol^–1^ Hz^–2^,  and *N*_*mfv*_ = 100. The residue sequence numbers of the atoms are indicated at the atom names.

When determining RDC values from a molecular trajectory using only magnetic-field vector RDC restraining (*K*^RDC.*msy*^ = 0), i.e., without RDC restraining of the molecule, the obtained RDC values will mainly depend on the set of target  values used because of the procedure applied of fitting <*D*_*k*_> to . Thus, the use of different subsets of target RDCs may result in differences in RDC values. Table 5 shows the RDC values obtained for different (sub)sets of RDCs. As expected, the *RMSD*(<*D*_*k*_>−*D*_*k*_^0^) values for the set of RDC-restrained RDCs (*rRMSD*) become lower the lower the number *N*_RDC_ of restraints applied. For all three subsets of RDCs, the *RMSD*(<*D*_*k*_>−*D*_*k*_^0^) values for the set of unrestrained RDCs (*urRMSD*) are rather large: 5.7, 6.4, and 5.5 Hz when restraining the 29 backbone RDCs, restraining the 6 backbone H–N RDCs or restraining the 7 backbone HB-CB RDCs, respectively. This means that the magnetic-field vector orientation distribution is specific for the (sub)set of RDCs used in the restraining. Figure 3 shows orientation distributions for the four vectors C_α_(1)–C_α_(7), C_α_(3)–C(3), C_α_(4)–C(4), and C_α_(5)–C(5) resulting from these simulations. The distributions should not be flat because of the magnetic-field vector RDC restraining, but this can barely be observed, *a.o.* due to the noise in the data.

**Table 5:** List of 39 RDC-Values (Hz) Derived from Experiment, , and the Averages <*D*_*k*_> from MD Simulations of the ß-Heptapeptide Solvated in Methanol Without any Restraining of the Molecule (*MDsol*, *K*^RDC,*msy*^ = 0 kJ mol^–1^ Hz^–2^) Using Four Different (Sub)sets of RDCs^a^

RDC sequence number	RDC atoms		(Hz)	(Hz)				(Hz)
				*RDCa*	*RDCbb*	*RDCHN*	*RDCHBCB*	*ATrEMvac*
1	^1^H(2)	^15^N(2)	3.4	3.5	3.1	3.3	**2.9**	2.3
2	^1^H(3)	^15^N(3)	5.6	5.3	5.3	5.4	**6.2**	4.0
3	^1^H(4)	^15^N(4)	4.4	4.5	4.8	4.5	**3.4**	3.0
4	^1^H(5)	^15^N(5)	4.5	4.3	4.2	4.4	**3.4**	3.0
5	^1^H(6)	^15^N(6)	5.2	4.9	5.0	4.9	**6.2**	3.8
6	^1^H(7)	^15^N(7)	4.7	4.6	4.5	4.4	**3.5**	3.4
7	^1^H_ß_(1)	^13^C_ß_(1)	–6.9	–6.5	–6.8	***–0.4***	–6.8	–6.2
8	^1^H_ß_(2)	^13^C_ß_(2)	–12.3	–12.3	–12.2	**–13.2**	–12.4	*–9.6*
9	^1^H_ß_(3)	^13^C_ß_(3)	–17.1	–15.3	–16.2	***–11.2***	–16.6	*–11.7*
10	^1^H_ß_(4)	^13^C_ß_(4)	–12.0	–12.1	–12.0	**–13.1**	–12.2	–10.2
11	^1^H_ß_(5)	^13^C_ß_(5)	–11.3	–11.1	–11.1	**–11.3**	–11.2	–10.0
12	^1^H_ß_(6)	^13^C_ß_(6)	–12.6	–12.1	–12.3	**–11.2**	–12.7	*–9.9*
13	^1^H_ß_(7)	^13^C_ß_(7)	–12.8	–11.7	–12.4	**–14.1**	–12.7	*–9.5*
14	^1^H_αRe_(1)	^13^C_α_(1)	–6.2	–6.4	–6.5	***–2.1***	***–8.8***	–6.0
15	^1^H_αRe_(2)	^13^C_α_(2)	–11.0	–10.6	–10.7	***–13.5***	**–12.3**	***–**8.6*
16	^1^H_αRe_(4)	^13^C_α_(4)	–12.5	–12.5	–12.5	**–13.6**	**–13.6**	***–**9.1*
17	^1^H_αRe_(6)	^13^C_α_(6)	–8.6	–9.7	–9.5	***–10.9***	***–12.3***	–10.2
18	^1^H_αRe_(7)	^13^C_α_(7)	–8.7	–8.5	–8.3	***–5.9***	***–4.0***	–7.3
19	^1^H_αSi_(1)	^13^C_α_(1)	1.5	1.4	1.2	***–13.0***	***–7.0***	*4.8*
20	^1^H_αSi_(2)	^13^C_α_(2)	6.9	7.1	7.2	***19.0***	***17.1***	5.2
21	^1^H_αSi_(5)	^13^C_α_(5)	10.1	9.7	*9.8*	***15.6***	**8.2**	*6.8*
22	^1^H_αSi_(6)	^13^C_α_(6)	7.1	6.1	6.3	***–1.6***	***0.3***	5.8
23	^1^H_αSi_(7)	^13^C_α_(7)	2.2	2.4	2.4	***10.0***	***7.4***	3.5
24	^1^H_αRe_(1)	^1^H_αSi_(1)	–1.5	–1.2	–1.2	***–5.2***	***–7.0***	*0.9*
25	^1^H_αRe_(2)	^1^H_αSi_(2)	1.1	0.6	0.7	**3.0**	***4.4***	1.3
26	^1^H_αRe_(3)	^1^H_αSi_(3)	–0.9	–1.4	–1.2	***–4.2***	***–10.7***	***–**4.3*
27	^1^H_αRe_(5)	^1^H_αSi_(5)	2.4	2.5	2.3	***6.7***	***6.0***	1.2
28	^1^H_αRe_(6)	^1^H_αSi_(6)	–0.2	0.7	0.3	***–4.8***	***–4.3***	***–**2.5*
29	^1^H_αRe_(7)	^1^H_αSi_(7)	–2.0	–2.1	–2.3	***5.8***	***3.9***	*1.6*
30	^1^H_γ_(1)	^13^C_γ_(1)	–1.2	–1.4	**–1.1**	***–3.4***	***1.9***	***3.6***
31	^1^H_γRe_(3)	^13^C_γ_(3)	–2.9	–3.3	***2.4***	***4.2***	***5.2***	***4.8***
32	^1^H_γSi_(3)	^13^C_γ_(3)	4.1	2.8	***–10.4***	***–7.0***	***–11.0***	***1.6***
33	^1^H_γRe_(3)	^1^H_γSi_(3)	0.8	1.1	***–4.1***	***–5.4***	***–3.8***	***8.6***
34	^1^H_δ_(3)	^13^C_δ_(3)	6.0	5.9	***9.1***	***16.8***	***14.5***	***–5.3***
35	^1^H_γ_(5)	^13^C_γ_(5)	–4.1	–4.2	**–5.7**	***–0.4***	**–2.2**	***–11.4***
36	^1^H_γRe_(7)	^13^C_γ_(7)	–3.6	–4.0	***–1.4***	***–1.1***	***0.8***	***3.6***
37	^1^H_γSi_(7)	^13^C_γ_(7)	2.3	1.6	***–3.9***	***–10.3***	***–3.5***	***–7.4***
38	^1^H_γRe_(7)	^1^H_γSi_(7)	–1.4	–1.1	**–2.3**	***–8.4***	**–2.6**	**0.4**
39	^1^H_δ_(7)	^13^C_δ_(7)	6.0	5.8	**6.3**	**5.9**	***11.0***	***2.3***
*RMSD*				0.6	*2.9*	*5.8*	*5.0*	*4.1*
*rRMSD*				0.6	0.4	0.2	0.2	*2.3*
*urRMSD*				-	***5.7***	***6.4***	***5.5***	***7.0***

a *RDCa*: all 39 RDCs, *RDCbb*: only the 29 backbone RDCs, No 1–29. *RDCHN*: only the 6 H–N backbone RDCs, No 1–6. *RDCHBCB*: only the 7 backbone HB–CB RDCs, No 7–13. Parameter values of the *t^msy^* = 100 ns *HRS* simulations are *γ^mfv^* = 2.4 ps^–1^, *K*^RDC,*mfv*^ = 100 kJ mol^–1^ Hz^–2^, *N_mfv_* = 100, and *ΔD^fb^* = 2.0 Hz. *ATrEMvac*: Energy-minimised (simulated annealing), RDC-, torsional-angle- and NOE-distance-restrained structure of the peptide in vacuo from ref (24) The residue sequence numbers of the atoms are within parentheses. RDC values  from Tables 1 and 2 of ref (24). The values for the RDCs that are not part of the subset of RDC restraints applied are in bold. *RMSD*: RMSD values calculated over all, *mfv*-restrained and unrestrained, RDCs. *rRMSD*: RMSD values calculated over the particular subset of *mfv*-restrained RDCs. *urRMSD*: RMSD values calculated over the unrestrained RDCs. Deviations of averaged RDC values  from their target values  larger than 2 Hz are in italics.

These results demonstrate the limited usefulness of the measured RDC values for molecular structure determination. In contrast to other quantities such as ^3^*J*-couplings observable by NMR techniques, an RDC is not defined in terms of a single structure, but by a (two-dimensional) orientation or rotational distribution of the molecule with respect to the magnetic field direction. In addition, since this distribution cannot be measured, it is common practice to generate such a distribution by fitting *N*_RDC_ calculated RDC values <*D*_*k*_> to a set of *N*_RDC_ measured, target RDC values (*k* = 1, 2, ..., *N*_RDC_). Since fitting of <*D*_*k*_> to  by varying the orientation or rotational distribution is relatively easy, because of the large variability offered by a two-dimensional orientation distribution (in the *HRS* case consisting of *N*_*mfv*_ configurations), the orientation distribution obtained through fitting strongly depends on the set of target RDCs and RDC values used in the fitting. This renders measured RDC values less useful for the structure determination of molecules.

### 5.5 RDC Values of MD Simulations in Methanol Solution with RDC Restraining of the Molecule (*HRSrMDsol*) Using Subsets of the 39 RDC Values Derived from Experiment

Since for a simulation with *K*^RDC.*msy*^ = 0, there are no RDC-restraining forces acting on the molecule, the dependence of the molecular structures upon the particular set of target RDC values used in the RDC-restraining can only be evaluated using *K*^RDC.*msy*^ > 0. Table S10 corresponds to Table 5, but for *K*^RDC,*msy*^ = 0.05 kJ mol^–1^ Hz^–2^ and  . It shows a similar picture to that of Table 5.

The molecular structure is not changing much by RDC-restraining of the molecular degrees of freedom. Table S11 shows the average values of the torsional angles of the molecule and their fluctuations obtained by RDC restraining of the molecule to the four (sub)sets of target RDC values. The average torsional-angle values are very similar to those when only the magnetic-field vector is restrained (*K*^RDC,*msy*^ = 0), the largest difference being only 26°.

### 5.6 Comparison of Rotational-Sampling and Alignment-Tensor Approaches

**Figure 4:**
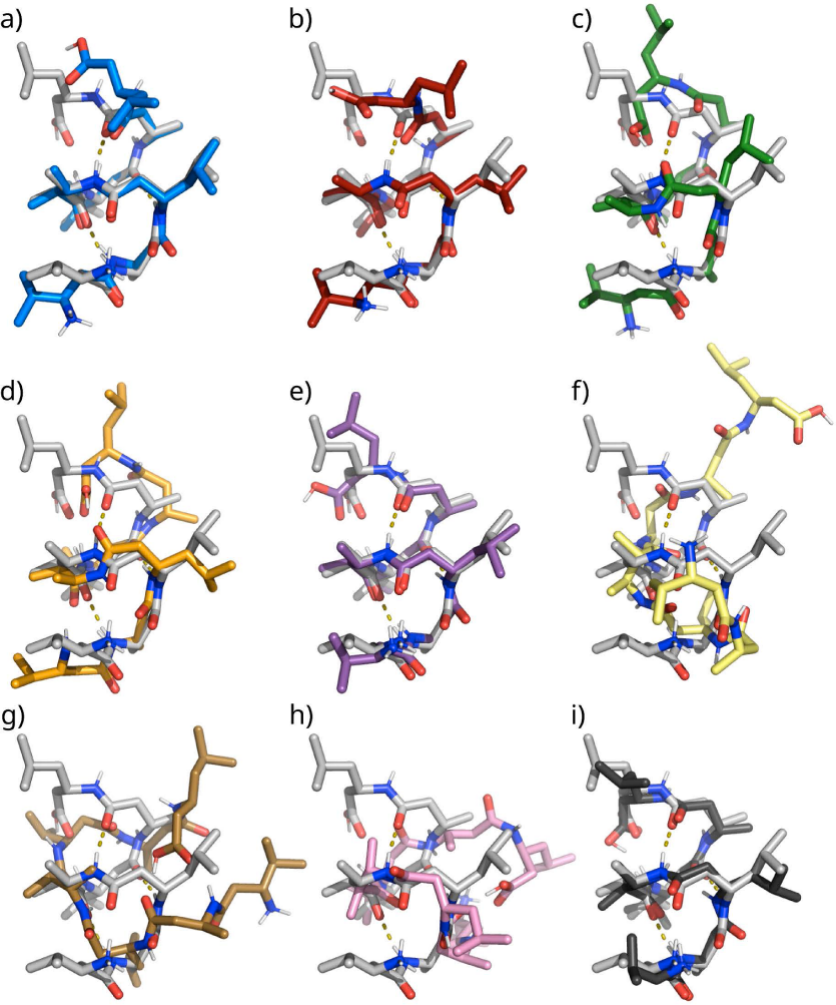
Initial structure and final trajectory structures from the various simulations of the ß-heptapeptide, and the model structure *ATrEMvac* obtained by a simulated annealing procedure in ref (24) From top left to bottom right: a) *MDsol* (blue), b) *HRSrMDsol* (red), c) *SDvac* (green), d) *HRSrSDvac* (orange), e) *ATrMDsol(0.1)* (violet), f) *ATrMDsol(10)* (yellow), g) *ATrSDvac(0.1)* (brown), h) *ATrMDvac(10)* (pink), and (i) *ATrEMvac* (black). The initial and other structures were superimposed using the backbone atoms of residues 2 to 6.

**Figure 5:**
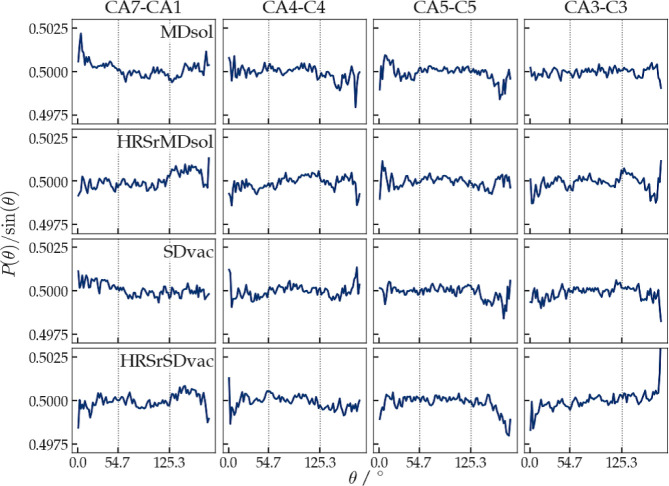
Distribution of the angle *θ*_*ab,H*_ between the four vectors , where *a* and *b* indicate the four atom pairs C_α_(1)–C_α_(7), C_α_(3)–C(3), C_α_(4)–C(4), and C_α_(5)–C(5), and the magnetic field direction  from MD or SD simulations of the ß-heptapeptide solvated in methanol or in vacuo (*MDsol, SDvac*) without any restraining of the molecule (*K*^RDC,*msy*^ = 0), and with (*K*^RDC,*msy*^ = 0.05 kJ mol^–1^ Hz^–2^, ) RDC restraining (*HRSrMDsol*, *HRSrSDvac*) of the molecule. Parameter values of the *t*^*msy*^ = 100 ns simulations are *γ*^*mfv*^ = 2.4 ps^–1^, *ΔD*^*fb*^ = 2.0 Hz, *K*^*RDC,mfv*^ = 100 kJmol^–1^Hz^2–^, , and *N*_*mfv*_ = 100. The residue sequence numbers of the atoms are indicated at the atom names.

**Figure 6:**
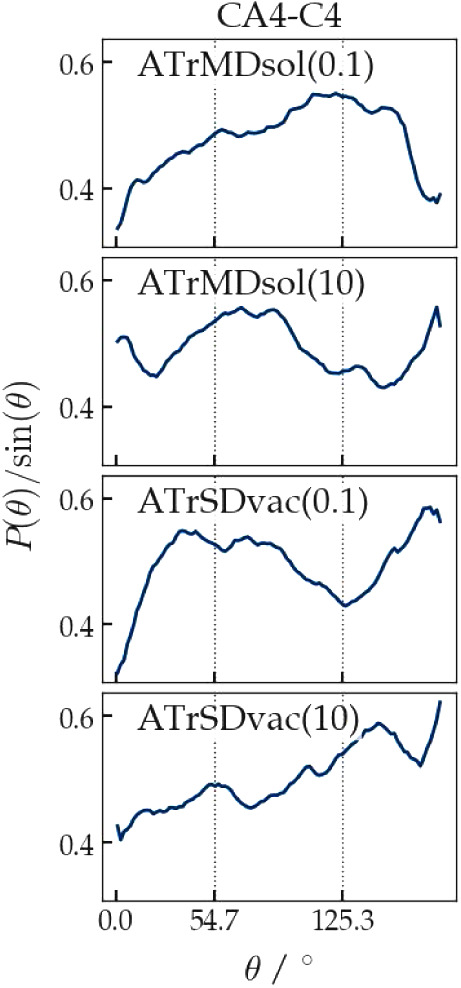
Distribution of the angle *θ*_*ab,H*_ between the four vectors , where *a* and *b* indicate the atom pair C_α_(4)–C(4), and the magnetic field direction  from RDC-restrained (*ΔD*^*fb*^ = 2.0 Hz) *t*^*AT*^ = 100 ns MD or SD (*γ*_*solv*_ = 60 ps^–1^) simulations of the ß-heptapeptide solvated in methanol (*ATrMDsol*) or in vacuo (A*TrSDvac*) using the alignment-tensor (*AT*) formalism with  and *K*^RDC,*AT*^ = 0.1 or 10 kJ mol^–1^ Hz^–2^. The residue sequence numbers of the atoms are indicated at the atom names.

The *HRSrMDsol* simulations can be compared to corresponding *ATrMDsol* simulations, see the results for simulations *MDsol*, *HRSrMDsol,* and *ATrMDsol* in Tables 1–4 and 6 and Figures 4–6. Using the alignment-tensor method, the *RMSD*(<*D*_*k*_>−) values in Table 4 are 3.3, 3.3, 6.1, and 7.0 Hz using an RDC-restraining force constant *K*^RDC,*AT*^ = 0, 0.1, 1, or 10 kJ mol^–1^ Hz^–2^, respectively. These values are much higher than those, 0.6 Hz for both *MDsol* and *HRSrMDsol*, obtained by applying the *HRS* method. This can be explained by the different procedures to match the RDC value of a trajectory structure with its target RDC value. In the *AT* approach, the five parameters determining the alignment tensor are for each trajectory structure varied to minimize the differences between *D*_*k*_ and , while in the *HRS* method, the orientation distribution of the magnetic-field vector  is biased to drive < *D*_*k*_ > toward . Since the *HRS* parameter *N*_*mfv*_ is generally larger than five, the *HRS* method allows more freedom to the orientation distribution of the magnetic-field vector (or equivalently of the molecule) to match <*D*_*k*_> with , than the *AT* method. Table 3 shows that for *K*^RDC,*AT*^ = 1 or 10 kJ mol^–1^ Hz^–2^, the backbone hydrogen bonds characteristic for the helical structure of the molecule are (largely) lost. For these values of the *AT* force constant, the violations of the NOE atom–atom distance upper bounds do increase (Table 1). We note that the three NOE-bound violations larger than 0.1 nm, mentioned in Section 5.2, No 106 (0.21 nm), H–C_β_(2)–H–C_δ_(3), No 111 (0.23 nm), H–N(4)–Me–C_δ1_(5) and No 115 (0.12 nm), H–N(6)–H–C_β_(7), are also violated in the *ATrMDsol* simulations, except when applying the large *AT* force-constant values *K*^RDC,*AT*^ = 1 or 10 kJ mol^–1^ Hz^–2^. The deviations of the simulated from the experimental ^3^*J*-couplings also increase the larger the value of *K*^RDC,*AT*^ becomes (Table 2).

**Table 6:** Averages and Root-Mean-Square Fluctuations (RMSF) of 19 Backbone and 6 Side-Chain Torsional Angles (Degree) from Unrestrained and RDC-Restrained (*ΔD*^*fb*^ = 2.0 Hz) *t*^*msy*^ = *t*^*AT*^ = 100 ns MD Simulations of the ß-Heptapeptide^a^

		(Average) angle and fluctuations (degree)								
				*HRSrMDsol*		*ATrMDsol*				
		*MDsol*		*K*^RDC,*msy*^ = 0.05 kJ mol^–1^ Hz^–2^		*K*^RDC,*AT*^ = 0.1 kJ mol^–1^ Hz^–2^		*K*^RDC,*AT*^ = 10 kJ mol^–1^ Hz^–2^		*ATrEMvac*
*Simulation*	Torsional angle	<angle>	*RMSF*	<angle>	*RMSF*	<angle>	*RMSF*	<angle>	*RMSF*	angle
backbone										
1	N(1)–C_ß_(1)–C_α_(1)–C(1)	73	15	74	17	74	19	86	41	46
2	C_ß_(1)–C_α_(1)–C(1)–N(2)	–52	147	–43	147	–51	146	65	126	–132
3	C(1)–N(2)–C_ß_(2)–C_α_(2)	–126	17	–125	19	–125	18	–110	44	–146
4	N(2)–C_ß_(2)–C_α_(2)–C(2)	58	11	55	23	59	15	74	86	65
5	C_ß_(2)–C_α_(2)–C(2)–N(3)	–132	17	–117	67	–120	60	22	106	–124
6	C(2)–N(3)–C_ß_(3)–C_α_(3)	–128	15	–129	15	–129	15	–92	70	–162
7	N(3)–C_ß_(3)–C_α_(3)–C(3)	58	11	56	9	56	10	48	69	58
8	C_ß_(3)–C_α_(3)–C(3)–N(4)	–119	72	–142	10	–141	13	30	99	–103
9	C(3)–N(4)–C_ß_(4)–C_α_(4)	–124	11	–125	10	–124	11	–116	51	–158
10	N(4)–C_ß_(4)–C_α_(4)–C(4)	51	12	51	9	49	18	–30	56	40
11	C_ß_(4)–C_α_(4)–C(4)–N(5)	–140	11	–140	10	–139	11	–110	44	–138
12	C(4)–N(5)–C_ß_(5)–C_α_(5)	–123	10	–123	9	–124	10	–103	39	–121
13	N(5)–C_ß_(5)–C_α_(5)–C(5)	62	12	61	10	63	11	76	44	60
14	C_ß_(5)–C_α_(5)–C(5)–N(6)	–106	99	–129	69	–103	103	56	83	–172
15	C(5)–N(6)–C_ß_(6)–C_α_(6)	–125	17	–124	14	–124	18	–108	64	–118
16	N(6)–C_ß_(6)–C_α_(6)–C(6)	69	36	69	30	68	38	59	102	68
17	C_ß_(6)–C_α_(6)–C(6)–N(7)	–2	142	–6	145	24	141	15	107	–121
18	C(6)–N(7)–C_ß_(7)–C_α_(7)	–116	18	–117	18	–116	18	–108	44	–149
19	N(7)–C_ß_(7)–C_α_(7)–C(7)	68	62	69	59	67	62	73	62	81
side-chains										
20	C_α_(1)–C_ß_(1)–C_γ_(1)–C_δ1_(1)	28	96	31	101	38	99	49	104	–68
21	C_α_(3)–C_ß_(3)–C_γ_(3)–C_δ_(3)	79	70	79	64	81	67	98	77	121
22	C_ß_(3)–C_γ_(3)–C_δ_(3)–C_ε1_(3)	80	68	78	64	78	69	81	91	–109
23	C_α_(5)–C_ß_(5)–C_γ_(5)–C_δ1_(5)	50	92	55	93	29	92	52	95	–138
24	C_α_(7)–C_ß_(7)–C_γ_(7)–C_δ_(7)	90	84	86	86	88	84	95	85	121
25	C_ß_(7)–C_γ_(7)–C_δ_(7)–C_ε1_(7)	84	85	85	84	86	82	77	93	–146

a *MDsol*: MD simulation of the peptide solvated in methanol without any restraining of the molecule (*K*^RDC,*msy*^ = 0 kJ mol^–1^ Hz^–2^ using the *HRS* method). *HRSrMDsol*: RDC-restraining MD simulations with *K*^RDC,*msy*^ = 0.05 kJ mol^–1^ Hz^–2^ and . The *mfv* parameters of the *HRS* method have the values *γ^mfv^* = 2.4 ps^–1^, *K*^RDC,*mfv*^ = 100 kJ mol^–1^ Hz^–2^,  and *N_mfv_* = 100. *ATrMDsol*: RDC-restraining MD simulations of the peptide solvated in methanol using the alignment-tensor (*AT*) formalism with  and *K*^RDC,*AT*^ = 0.1 or 10 kJ mol^–1^ Hz^–2^. *ATrEMvac*: Energy-minimised (simulated annealing), RDC-, torsional-angle- and NOE-distance-restrained structure of the peptide in vacuo from ref (24). The residue sequence numbers of the atoms are within parentheses.

The structure of the molecule in terms of average torsional-angle values and fluctuations changes significantly for *K*^RDC,*AT*^ = 10 kJ mol^– 1^Hz^–2^ compared to the *HRS* results (Table 6).

The *HRS* orientation distributions, for *MDsol* and *HRSrMDsol* in Figure 5, and the *AT* orientation distributions, for *ATrMDsol(0.1)* and for *ATrMDsol(10)* in Figure 6, are rather different. The variation in the *AT*-generated distributions is much larger than that in the *HRS*-generated distributions. This is the result of the *HRS* method using an arbitrary magnetic-field orientation distribution when fitting calculated to target RDC values, whereas the *AT* method is based on a magnetic-field orientation distribution in terms of spherical harmonic functions of order 2. The final structures of the various simulations shown in Figure 4 also illustrate the different results obtained by the *HRS* and *AT* approaches.

### 5.7 Representation of Molecular Flexibility and Mobility

It is well known that for flexible molecules or parts of molecules, accounting for the presence of multiple different conformations in structure determination or refinement is mandatory.^5−12^ This is also true when calculating RDC values using the *AT* approach.

Table 4 shows that the RDC values calculated from the *ATrMDsol* simulations become smaller in absolute value when the value of the restraining force constant *K*^RDC,*AT*^ is increased. This can be explained by the presence of multiple conformations in the simulations. When the molecule is switching between different conformations, for which the RDC vectors have different angles with the magnetic field vector (or different, positive, and negative RDC values), the *AT* approach without time or molecule averaging results in an average, reduced RDC value.

### 5.8 Comparison of the *HRS* Generated Structures and Their Properties with a Structure of the β-Heptapeptide Obtained by Simulated Annealing In Vacuo

Tables 1–4 and 6 and Figure 4 also allow a comparison of the trajectory structures generated in the *HRS* and *AT* simulations of the molecule in methanol solution with the molecular structure as available in Supporting Information of ref (24). The latter structure, here indicated as *ATrEMvac*, was obtained from a simulated annealing procedure^24^ using the program XPLOR-NIH by applying not only 29 backbone RDC restraints but also 124 NOE atom–atom distance upper bound restraints (5 more than the 119 bounds analyzed here) listed in the Supporting Information of ref (24) (ref (24) itself mentions 126 bounds), and 11 backbone dihedral-angle restraints. The latter were derived from the experimentally determined ^3^*J*-coupling values. The variation of the 11 backbone torsional angles was limited to ±40°. In total, 164 restraints were applied. The single-structure simulated annealing procedure^24^ involved energy minimization (EM) in vacuo (no methanol solvent around the molecule), so no time- or conformational averaging, and the program PALES^66^ was used as a fitting tool to select the structure among the lowest energy conformers that showed the best fit to the experimental RDC values. Since the program PALES uses the alignment-tensor formalism, no rotational sampling was applied to obtain structure *ATrEMvac*.

Table 4 shows an *RMSD*(<*D*_*k*_>−)-value of 4.1 Hz for *ATrEMvac*, which is much larger than the value 0.6 Hz obtained for *MDsol* and *HRSrMDsol*. For 21 out of 39 RDCs, the deviation between the calculated and experimentally derived RDC values is larger than 2 Hz. The six ^1^H–^15^N RDCs, which reflect the direction of the helical hydrogen bonds, are well reproduced in *ATrEMvac*. Yet, the NH (4)–O(6) backbone hydrogen bond is not present (Table 3).

Table 5 allows a comparison between structure *ATrEMvac*, which was obtained using 29 backbone target RDC values and the unrestrained *MDsol* simulation *RDCbb*, in which the same set of 29 backbone target RDC values was used to calculate RDC values. For the latter simulation, the *RMSD*(<*D*_*k*_>−) value is 2.9 Hz, with 6 deviations larger than 2 Hz between calculated and target RDC values, to be compared to a value of 4.1 Hz for the *ATrEMvac* structure, with 21 deviations larger than 2 Hz between calculated and target RDC values.

As expected, the *ATrEMvac* structure largely satisfies the upper bounds of the NOE atom–atom distance (Table 1). Only one bound is violated by more than 0.1 nm, between H–N(5) and H–N(6) (No 31) of the set of 42 bounds of ref (45) (which bound is absent in the set of 119 bounds of ref (24). The corresponding RMSD values of the bound violations are *RMSD42* = 0.02 nm and *RMSD119* = 0.03 nm. Although in the *MDsol* and *HRSrMDsol* simulations no NOE atom–atom distance upper-bound restraints were applied, in contrast to the procedure leading to *ATrEMvac*, these simulations show 0 and 3 bound violations larger than 0.1 nm for the set of 42 bounds of ref (45) and the set of 119 bounds of ref (24), respectively. The corresponding RMSD values of the bound violations are *RMSD42* = 0.01 nm and *RMSD119* = 0.03 nm.

Table 2 shows the ^3^*J*-coupling values obtained for the *ATrEMvac* structure. For five ^3^*J*-couplings, the deviation from the experimentally derived value is larger than 2 Hz, H_ß_(1)–C_ß_(1)–C_α_(1)–H_αSi_(1) (No 1) with a deviation of 2.4 Hz (from the value of ref (45) H_ß_(7)–C_ß_(7)–C_α_(7)–H_αSi_(7) (No 6) with a deviation of −2.7 Hz (from the value of ref (45) H_ß_(1)–C_ß_(1)–C_γ_(1)–H_γ_(1) (No 7) with a deviation of 8.1 Hz (from the value of ref (45) and 7.7 Hz (from the value of ref (24) H_ß_(5)–C_ß_(5)–C_γ_(5)–H_γ_(5) (No 8) with a deviation of −4.5 Hz (from the values of refs. (24, 45) and H_N_(3)–N(3)–C_ß_(3)–H_ß_(3) (No 10) with a deviation of −3.1 Hz (from the value of ref (45) or −2.7 Hz from the value of ref (24). The overall RMSD value for structure *ATrEMvac* is 2.5 Hz, which is also larger than 2 Hz. We note that the application of torsional-angle restraints that do not account for the multiplicity of the inverse function of the Karplus relation expressing ^3^*J*-couplings in terms of molecular torsional angles^5^ unphysically limits the motion or the conformational variability along the torsional-angle degrees of freedom of the molecule. Although in the *MDsol* and *HRSrMDsol* simulations, no torsional-angle restraints were applied, in contrast to the procedure leading to *ATrEMvac* (11 torsional-angle restraints), these simulations show zero deviations larger than 2 Hz and an RMSD value with respect to the experimentally derived ^3^*J*-couplings of 0.5 Hz.

The torsional-angle values of the *ATrEMvac* structure differ for 8 torsional angles significantly from the *MDsol* and *HRSrMDsol* ones (Table 6), backbone torsional angles C_ß_(1)–C_α_(1)–C(1)–N(2) (No 2), C_ß_(5)–C_α_(5)–C(5)–N(6) (No 14), and C_ß_(6)–C_α_(6)–C(6)–N(7) (No 17), and side-chain torsional angles C_α_(1)–C_ß_(1)–C_γ_(1)–C_δ1_(1) (No 20), C_ß_(3)–C_γ_(3)–C_δ_(3)–C_ε1_(3) (No 22), C_α_(5)–C_ß_(5)–C_γ_(5)–C_δ1_(5) (No 23), and C_ß_(7)–C_γ_(7)–C_δ_(7)–C_ε1_(7) (No 25).

### 5.9 Comparison Structure Refinement in Solution and In Vacuo

Structure refinement based on RDCs is commonly carried out using the vacuum boundary condition, i.e., without any explicit solvent molecules present in the molecular system. For the β-heptapeptide, it has been shown that the helical structure is severely distorted when simulated by MD or SD in vacuo^67^ without any restraints. Thus, we compared structure refinement of the peptide in vacuo (*HRS* or *AT rSDvac*) with that in a methanol solution (*HRS* or *AT rMDsol*). Table S12 shows an *RMSD*(<*D*_*k*_>−*)* value of 0.5 Hz for *SDvac* and a value of 0.4 Hz for *HRSrSDvac*, which are comparable to the value of 0.6 Hz obtained for *MDsol* and *HRSrMDsol*. The *ATrSDvac* simulations result in much larger values, 5.5 Hz when *K*^RDC,*AT*^ = 0.1 kJ mol^–1^ Hz^–2^, and 7.0 Hz when *K*^RDC,*AT*^ = 10 kJ mol^–1^ Hz^–2^.

In the *SDvac* and *HRSrSDvac* simulations, the three backbone hydrogen bonds are still present, although the occurrence of the NH(4)–O(6) one is much lower than when methanol solvent is present (Table S13). The two *ATrSDvac* simulations show very little backbone hydrogen bonding. Surprisingly, the *SDvac* simulation shows a low number of violations of the upper bounds of the NOE atom–atom distance (Table S14). Only one bound is violated by more than 0.1 nm, between H–N(4) and H–C_β_(6) of the set of 42 bounds of ref (45) and 3 violations of the bounds of the set of 119 bounds of ref (24). The corresponding RMSD values of the bound violations are *RMSD42* = 0.03 nm and *RMSD119* = 0.03 nm, only slightly larger than when methanol solvent is present (0.01 and 0.03 nm, respectively). The *ATrSDvac* simulations show a considerably increased number of bound violations and larger *RMSD42* and *RMSD119* values. We note that of the three NOE upper bound violations larger than 0.1 nm mentioned before, No 106, H–C_β_(2)–H–C_δ_(3), shows a small violation (0.05, 0.02 nm) in the *ATrSDvac* simulations and No 115, H–N(6)–H–C_β_(7), virtually no violation (0.01 nm) for *ATrSDvac(10)*.

Table S15 shows the ^3^*J*-coupling values as obtained from simulations in vacuo (*SDvac*, *HRSrSDvac,* and *ATrSDvac*), which are to be compared to the ones obtained in methanol (*MDsol* and *HRSrMDsol*). The *SDvac* simulation shows only one deviation between simulated and experimentally derived values larger than 2 Hz, for the side-chain torsional angle H_ß_(5)–C_ß_(5)–C_γ_(5)–H_γ_(5) with a deviation of −3.0 Hz. RDC restraining (*HRSrSDvac*) adds another deviation larger than 2 Hz, H_ß_(3)–C_ß_(3)-–_α_(3)–H_αRe_(3) with a deviation of −2.2 Hz. The RMSD values for the ^3^*J*-couplings in these simulations in vacuo are, with 1.2 Hz (*SDvac*) and 1.1 Hz (*HRSrSDvac*), larger than those for the simulations in methanol, with an RMSD value of 0.5 Hz for *MDsol* as well as *HRSrMDsol*. The *ATrSDvac* simulations show much larger deviations with RMSD values of 2.1 Hz (*ATrSDvac(0.1)*) and 2.5 Hz (*ATrSDvac(10)*).

The structure of the molecule in terms of average torsional-angle values and fluctuations changes significantly when the methanol solvent environment of the molecule is omitted (Table S16). In the *SDvac* simulation, five backbone angles C_ß_(2)–C_α_(2)–C(2)–N(3) (No 5), C_ß_(3)–C_α_(3)–C(3)–N(4) (No 8), C(3)–N(4)–C_ß_(4)–C_α_(4) (No 9), C_ß_(5)–C_α_(5)–C(5)–N(6) (No 14), and C_ß_(6)–C_α_(6)–C(6)–N(7) (No 17) show large changes. Large changes in fluctuations are observed for these backbone angles except for C_ß_(3)–C_α_(3)–C(3)–N(4) (No 8). The *ATrSDvac* simulations show rather large changes in average torsional-angle values and fluctuations for the same backbone angles and a few more. Figure 6 shows the differences between the final structures obtained in methanol (*MDsol*, *HRSrMDsol*) and in vacuo (*SDvac*, *HRSrSDvac, ATrSDvac*).

**Figure 7:**
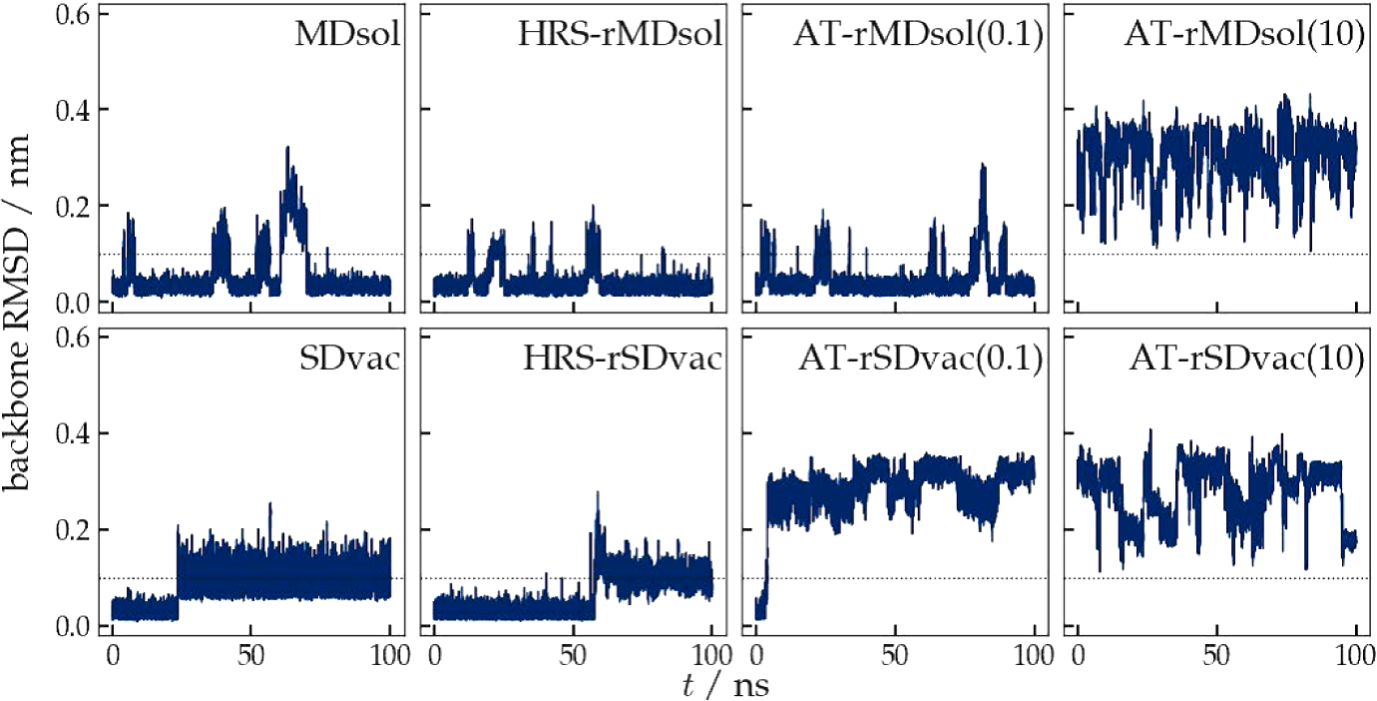
Backbone atom-positional root-mean-square deviation (RMSD) from the left-handed *M*-3_14_-helical model structure (Figure 2) for residues 2 to 6 as a function of time for the different types of simulations.

In ref (67), it was shown that the helical structure of the β-heptapeptide is not stable when simulated in vacuo. Whether the dominance of the *M*-3_14_-helical structure of the β-heptapeptide solvated in methanol at 300 K and 1 atm is preserved in the different simulations presented here can be determined, apart from monitoring backbone hydrogen bonding, by showing the atom-positional root-mean-square deviation (RMSD) for the backbone atoms of residues 2 to 6 between the trajectory structures and the helical initial structure (Figure 7). The helical structure is lost in all simulations but recovered after short excursions only in the *MDsol*, *HRSrMDsol,* and *ATrMDsol(0.1)* simulations. To simulate the helical folding/unfolding equilibrium of the β-heptapeptide solvated in methanol, the presence of solvent molecules seems to be required.

### 5.10 Analysis of Three NOE Atom–Atom Distance Upper-Bound Violations

The three NOE-bound violations larger than 0.1 nm, mentioned in Section 5.2, that were observed in the *MDsol* and *HRSrMDsol* simulations, No 106 (0.21 nm, 0.18 nm), H–C_β_(2)–H–C_δ_(3), No 111 (0.13 nm, 0.13 nm), H–N(4)–Me–C_δ1_(5) and No 115 (0.12 nm, 0.12 nm), H–N(6)–H–C_β_(7), are discussed in more detail. We note that these NOE upper bounds are also violated in the *ATrMDsol* simulations, except for NOE No 106, H–C_β_(2)–H–C_δ_(3), and NOE No 111 (0.13 nm, 0.13 nm), H–N(4)–Me-C_δ1_(5), which show no violation in the *ATrMDsol(1)* and *ATrMDsol(10)* simulations. The location of these distance bounds is indicated in Figure 1. As was mentioned before, these violations could be due to a particular feature of the (GROMOS) force field used or the bounds derived from experiment (ref (24)) may be too tight.

The literature concerning the applications of the GROMOS force field to β-peptides in methanol shows that this force field is able, without any restraints, to reproduce experimentally derived data for these molecules.^45−50,68,69^ A folding equilibrium between left-handed and right-handed helical folds could be shown to resolve an NMR experimental paradox.^69^ A comparison of the *MDsol* simulation using the GROMOS force field without any restraints applied, with the *ATrEMvac* structure obtained using the X-PLOR force field^70^ in the presence of 164 restraints, 124 atom–atom distance, 11 torsional-angle, and 29 backbone RDC restraints, shows that in the *MDsol* simulation the experimental data are better reproduced than in the *ATrEMvac* structure (which was obtained by fitting to the experimental data).

1. Regarding NOE atom–atom distance upper bounds, for *MDsol*, *Nviol42* = 0, *RMSD42* = 0.01 nm, *Nviol119* = 3, *RMSD119* = 0.03 nm, while for *ATrEMvac*, *Nviol42* = 0, *RMSD42* = 0.02 nm, *Nviol119* = 0, *RMSD119* = 0.03 nm.
2. Regarding ^3^*J*-couplings, for *MDsol* there are no deviations from the experiment larger than 2 Hz and an RMSD value of 0.5 Hz, whereas for *ATrEMvac*, there are 5 deviations larger than 2 Hz and an RMSD value of 2.5 Hz.
3. Regarding RDCs, *MDsol* shows no deviations from experiment larger than 2 Hz and an RMSD value of 0.6 Hz between simulation and experiment, whereas the *ATrEMvac* structure shows 21 deviations larger than 2 Hz and an RMSD value of 4.1 Hz.

This illustrates the quality of the GROMOS force field.

Personal correspondence with the authors of ref (24) identified that the cross-peaks corresponding to distances 106, 111, and 115 are all weak, and the integration error is considerable. Moreover, in each of these cases, the cross-peaks could only be integrated on one side of the diagonal. In addition, we note that NOE No 111 involves the methyl group Me–C_δ1_(5), which is at 0.93 ppm. However, the methyl protons of Me–C_δ1_(5) and Me–C_δ2_(5) have very similar chemical shifts, with the latter at 0.94 ppm. Thus, the intensity of NOE No 111 could have been increased by peak overlap, i.e., could have contributions from both methyl groups. NOE No. 106 involves H–C_β_(2). The chemical shift of the proton is given as 4.58 ppm in ref (24), whereas in ref (25) it is listed as 4.41 ppm. Proton H–C_β_(3) also has a chemical shift of 4.41 ppm in both papers. So if the assignment in ref (25) is correct, NOE No 106 could have contributions from H–C_β_(2) and from H–C_β_(3), which would make it more intense and hence having a shorter distance upper bound than is realistic. NOE No. 115 involves H–C_β_(7) at 4.44 ppm. This chemical shift is close to 4.41 ppm, which is the value for H–C_β_(3) (and perhaps H–C_β_(2)) and so there is the possibility of peak overlap here too.

Finally, one may investigate whether a structure of the β-heptapeptide that more or less matches the four mentioned atom–atom NOE distance upper bounds also matches other experimentally derived data, such as ^3^*J*-couplings. Comparing the ^*3*^*J*-couplings, structure *ATrEMvac* shows an RMSD deviation from the experiment for the 21 ^3^*J*-couplings (Table 2) of 2.5 Hz with 5 deviations larger than 2 Hz, whereas this value is 0.5 Hz with zero deviations larger than 2 Hz for the *MDsol* and *HRSrMDsol* simulations. Among the five ^3^*J*-coupling deviations larger than 2 Hz in the *ATrEMvac* structure, there is one, H_ß_(5)–C_ß_(5)–C_γ_(5)–H_γ_(5) (No 8), that involves the torsional angle C_α_(5)–C_ß_(5)–C_γ_(5)–C_δ1_(5) (No 23), which may be influenced by the distance restraint of NOE No 111. The value of the torsional angle C_α_(5)–C_ß_(5)–C_γ_(5)–C_δ1_(5) is −138° in *ATrEMvac* to be compared to an average value of 50° in *MDsol* and 55° in *HRSrMDsol* (Table S16).

In view of the considerations regarding the three NOEs discussed above, we could conclude that integration errors and possible peak overlap are likely to have given rise to a too-short distance upper bound and hence NOE violations in the simulations.

## 6 Discussion and Conclusions

The experimental determination of residual dipolar couplings (RDCs) rests on sampling the rotational motion of a molecule in an environment that induces a slightly nonuniform, unfortunately immeasurable and unknown, orientation distribution of the molecule in solution. Averaging over this slightly nonuniform, anisotropic distribution reduces the size of the dipolar couplings (DCs) from the kHz range to the Hz range for the resulting RDCs, so by a factor of 10^3^ to 10^4^, due to compensation between large positive and negative values of the function *P*_*k*_(θ_*k*_(*t*)) that is being averaged. These two features hamper the use of measured RDCs to contribute to the structure determination or refinement of (bio)molecules. If, when modeling or in a molecular simulation, the nonuniform orientation distribution is generated by a chosen biasing potential-energy term in the potential-energy function, RDC values can straightforwardly be calculated from the computer-generated nonuniform orientation distribution. Experimentally, a nonuniform rotational distribution can be induced in different ways,^17^ for example, using the paramagnetic susceptibility of a molecule, using electrostatic interactions with a molecule, or, most commonly, by immersing the molecule in a medium that contains some order that will influence the angular distribution *P*(θ) of the angle θ of some axis in the molecule with the direction of the magnetic field. Modeling such anisotropy-inducing forces in a molecular simulation or structure calculation in such a way that it exactly matches experiment is virtually impossible, e.g., due to the difficulty of realistically representing macroscopic conditions at a molecular level of resolution.

In the alignment-tensor (*AT*) approach, the orientation distribution of the molecule with respect to a magnetic-field direction is not sampled, but described^20^ in terms of a basis set of five spherical harmonic functions of order 2. The real physical orientation distribution may, however, differ from the *AT* one described in terms of spherical harmonics of order 2. Contributions of orders different from 2 are not accounted for in the *AT* approach, in which the unknown orientation or alignment distribution of the molecule is represented by a so-called 3 × 3 alignment tensor, which depends on 5 parameters, 3 (Euler) angles, and 2 nonspherical symmetry parameters for the molecule. The 3 × 3 tensor is symmetric and has trace zero, so it has only 5 independent tensor elements. These represent the reduction of the size of the average dipolar couplings from the kHz range to the Hz range and the anisotropy of the orientation distribution of the molecule in terms of the 5 coefficients *a*_*m*_ (*m* = 1–5) of the five spherical harmonic basis functions of order 2, see e.g., refs (33),^34^

The 5 parameters determining the molecular alignment distribution can be determined in various ways.

1. Estimation (i) from the shape of the molecule,^71^ or (ii) from its moments of inertia,^72^ or (iii) from its gyration tensor,^73^ or (iv) from its hydrodynamic shape,^74^ or (v) from its alignment against a hypothetical wall, while considering its charge distribution.^66^
2. Use of a given set of (measured) RDC values by optimizing the agreement between the given and calculated RDCs of the set, which must contain sufficient RDC values. This optimization can be performed by a linear least-squares fit.^20^

In the *AT* formalism, the motions of the molecule can be accounted for in three different ways.

1. Orientational constraints^75^ or tensorial constraints^76^ can be applied to sample a limited part of the rotational space of the molecule.
2. Generalized order parameters *S* for RDC-vectors may be defined, which represent the small amplitude directional variation of these RDC-vectors, e.g., with values in the range [0.7–0.9].^77^ In the simulated annealing procedure for structure determination, the *S*-value, which is taken equal for all RDCs,^77^ is allowed to vary in order to minimize *RMSD*(<*D*_*k*_>−*D*_*k*_^0^), the root-mean-square difference between the calculated (*D*_*k*_), and target (*D*_*k*_^0^) RDC values.
3. An alternative is the use of multiple conformations in structure refinement, in which two or more (*N*_*c*_) conformations of the molecule are used with equal weights 1/*N*_*c*_ in the structure refinement procedure that minimizes *RMSD*(<*D*_*k*_>−).^78,79^

The introduction of additional parameters or conformational degrees of freedom in structure refinement leads, unavoidably, to lower *RMSD*(*D*_*k*_–) values. The mentioned extensions of the *AT* formalism allow for a representation of small amplitude motion of parts of the molecule but are not able to reflect the physical dynamics of the motion, and they ignore the statistical-mechanical Boltzmann (energy-dependent) weights of the different molecular configurations. This means that these extended *AT* approaches are expected to perform well for rigid molecules or when many structural restraints, i.e., NOE distance restraints, ^3^*J*-coupling derived torsional-angle restraints and hydrogen-bond restraints are applied.^78,79^ However, for flexible molecules, in the absence of other restraints rigidifying the molecule, these methods are not expected to perform well.

The past decades have shown many applications of measured RDC values in regard to structure determination or refinement of biomolecules, in particular proteins, see refs (80−82) and the studies described and quoted therein. These are based on the alignment-tensor (*AT*) formalism and thus are prone to uncertainties due to the validity of the assumptions on which the *AT* formalism rests. The assumption of rigidity of the molecule is, for example, addressed by postulating motional models for the RDC vector, e.g., the Gaussian axial fluctuations (*GAF*) model, for local parts of the molecule. Another way of approximately accounting for mobility or conformational variability is the application of a simulated annealing (*SA*) procedure, that is, lowering the temperature of the molecule in vacuo in order to enhance the search for conformations optimized regarding deviations between calculated and (measured) target RDC values and regarding (low) intramolecular energy. But, such conformations are not selected using statistical-mechanically correct Boltzmann weighting, as can be done by MD or SD simulation of the molecule in solution at physiological temperature and pressure.

In contrast to the *AT*-based approaches, the molecular rotational-sampling (*MRS*^22^) and magnetic-field rotational-sampling (*HRS*) approaches are based on extensive sampling of the molecular orientation distribution. In the *HRS* approach, investigated in the present article, this is done by SD simulation of the rotational motion of the (*mfv*) magnetic-field vector alternating with MD or SD simulation of the motions along the molecular degrees of freedom of the (*msy*) molecular system in combination with molecule-internal configurational averaging. It does not use an alignment tensor.

Application of the *HRS* algorithm to the β-heptapeptide solvated in methanol shows that, using appropriate *mfv* parameters (*K*^RDC,*mfv*^ = 100 kJ mol^–1^ Hz^–2^,  and *N*_*mfv*_ = 100) when simulating the rotational motion of the magnetic field, fitting the averaged RDC values <*D*_*k*_> to the measured target RDC values  already leads to an orientation distribution of the molecule that yields RDC values close to the measured ones, the *RMSD*(<*D*_*k*_>−) value calculated over the 39 RDCs being 0.6 Hz. In the current case of the β-heptapeptide in methanol solution, virtually no change in structure was required to match the  values.

The latter satisfies all 42 NOE-derived atom–atom distance upper bounds and 21 ^3^*J*-couplings derived from NMR experiments.^25^ It shows NOE distance upper bound violations for three pairs of atoms in a set of 119 NOE-derived atom–atom distance upper bounds.^24^ It has been argued that these three NOE distance upper-bound violations are not due to a possible deficiency of the GROMOS force field used in the simulations but rather to experimental cross-peak integration errors or possible overlap of some resonances of the ROESY spectra, giving rise to increased intensities and hence too short distance upper bounds.

The comparison of the molecular properties obtained without any restraining of the molecule (*MDsol*), with RDC-restraining of the molecule using either the *HRS* approach (*HRSrMDsol*) or the *AT* approach (*ATrMDsol*) shows that the latter reproduces RDCs not as well with an RMSD between calculation and experiment varying between 3.3 and 7.0 Hz for the different *AT* calculations (*ATrMDsol*) to be compared with 0.6 Hz for the unrestrained (*MDsol*) and *HRS* (*HRSrMDsol*) simulations. Only for very low RDC-restraining force-constant values (*K*^RDC,*AT*^ = 0 or 0.1 kJ mol^–1^ Hz^–2^) are the NOE distance upper bounds satisfied, the ^3^*J*-couplings reproduced, and the helical backbone hydrogen bonds maintained. For *K*^RDC,*AT*^ = 1 or 10 kJ mol^–1^ Hz^–2^, the number and size of the NOE upper-bound violations and ^3^*J*-coupling deviations from the experiment increase.

Simulating the β-heptapeptide in vacuo leads to a deformation of the molecule. Yet, the RDCs are still well reproduced in unrestrained (*SDvac*) and *HRS* RDC-restrained (*HRSrSDvac*) simulations due to the feature of the *HRS* algorithm that allows a detailed variation of the orientation distribution of the molecule in order to fit calculated RDC values to target RDC values.

The structure reported in ref (24) and obtained by simulated annealing in vacuo in the presence of 29 backbone RDC-restraints, 124 NOE upper-bound restraints, and 11 torsional-angle restraints (A*TrEMvac*) shows much larger discrepancies with the experiment than the unrestrained (*MDsol*) or RDC-restrained (*HRSrMDsol*) MD simulations of the β-heptapeptide solvated in methanol. This reflects the inadequacy of the assumptions on which the alignment-tensor formalism rests on the one hand and, on the other hand, illustrates the importance and dominant role of the force field used in structure refinement based on NMR data. In case of the β-heptapeptide, the GROMOS force field, a biomolecular force field based on free-energy data, leads to molecular trajectories that are completely compatible with the NMR data on the molecules, whereas the X-PLOR force field in combination with many restraints making use of the alignment-tensor approach appears to fail to properly reproduce the above-mentioned set of NMR data.

The present investigation illustrates the limitations of the assumptions on which the alignment-tensor approach in structure determination or refinement of molecular systems based on measured RDC values rests: (i) an orientation distribution of the molecule in terms of spherical harmonic functions of order 2, (ii) rigidity of the molecule, and (iii) the absence of coupling between rotational and internal motions of the molecule.

In addition, the application of the more general magnetic-field rotational sampling (*HRS*) algorithm demonstrates that experimentally measured RDCs are less useful for molecular structure determination or refinement than other observable quantities measurable by NMR techniques, such as NOE intensities, ^3^*J*-coupling constants and *S*^2^ motional order parameters, because of the particular characteristics of their definition in terms of an orientation distribution of the molecule of interest:

1. The real orientation distribution of the solute molecule of interest, which is needed to properly calculate the average, that is the RDC, is not measurable.
2. The real orientation distribution of the solute molecule of interest, which is needed to properly calculate the average, that is the RDC, cannot properly be modeled or simulated, because of the difficulty of realistically and accurately representing macroscopic conditions at the molecular level of resolution.
3. Therefore, the common way to obtain this orientation distribution is by fitting, for a particular set of RDCs, the RDC values (*D*_*k*_ or <*D*_*k*_>) calculated for one or more (modeled or MD-generated trajectory) structures to the (measured) target RDC values ().
4. This procedure leads to a rather strong dependence of the obtained orientation distribution and thus of the RDC values calculated from it upon the particular set of target  values used in the minimization procedure.

The definition and fundamental properties of RDCs and the way RDC values are calculated for a given structure, that is, by minimizing the difference between calculated and target (measured) RDC values, are independent of the particular molecule of interest, be it a peptide, protein, or any other type of molecule. They limit the usefulness of measured RDC values for structure determination or refinement of biomolecules.

In the present case of the β-heptapeptide, it seems that the question of whether the dominant helical conformation of the peptide in pure methanol solution is similar to the dominant conformation of the peptide in methanol with stretched polyvinyl acetate added cannot be answered using RDC values.

In comparison to other quantities observable by NMR techniques, such as NOESY or ROESY intensities, ^3^*J*-couplings or relaxation properties expressed as *S*^2^ order parameters, measured RDC values are fundamentally less useful for structure determination or refinement of biomolecules. While an NOE intensity can relatively straightforwardly be related to an atom–atom distance and a ^3^*J*-coupling to a torsional angle using a Karplus-type relation, there exists no direct relationship between an RDC and a molecular configuration due to its definition in terms of a rotational distribution of the angle between the RDC vector and the magnetic field. Since this distribution is immeasurable and cannot be reliably simulated at the molecular level of resolution and since the slight anisotropy of the rotational distribution of the molecule in the biasing medium reduces the RDC signal from the kHz range to the Hz range, that is over at least 3 orders of magnitude, the only viable procedure to calculate RDC values for a molecular structure is to apply a procedure in which the difference between calculated and (measured) target RDC values is minimized. These features introduce a much larger uncertainty in calculated RDC values and their effect in structure determination of refinement of biomolecules than the use of NOE intensities or ^3^*J*-couplings.

*S*^2^ order parameters are, like RDCs, also not defined in terms of a single molecular structure but in terms of a distribution of the direction of an atom–atom vector in the molecule. However, this distribution is not the overall rotational distribution of the molecule, but a local, directionally rather limited one. Such a distribution can be simulated rather well using MD simulation of the molecule in solution at physiological temperature and pressure. Thus, for the calculation of *S*^2^ order parameters, one need not resort to a procedure in which the difference between calculated and target *S*^*2*^ order parameters is minimized.^14^

The present investigation illustrates the inadequacy of applying single-structure determination or refinement procedures to flexible molecules or molecules with flexible parts, for which procedures do not properly account for molecular motion or the proper statistical-mechanical Boltzmann probabilities of occurrence of multiple conformations.

Summarizing, one could state that in structure determination or refinement of biomolecules based on NMR data, it seems mandatory to refrain from the vacuum boundary condition, from torsional-angle restraints that do not account for the multiplicity of the inverse function of the Karplus relation expressing ^3^*J*-couplings in terms of molecular torsional angles, to allow for Boltzmann-weighted time- or molecule-averaging of molecular conformations, and, not the least, to use a force field that has an adequate basis in thermodynamic data of biomolecules. Finally, in the case of RDC-restraining, a method that properly samples the (unmeasurable) rotational distribution or motion of the molecule is to be used. If the orientation or rotational distribution of the molecule is obtained by fitting calculated RDC values to measured target RDC values, the obtained calculated RDC values will be dependent on the values and set of target RDC values used in the fitting.

## Supporting Information Available

Table S1. Root-mean-square difference (*RMSD*) between the averages  from the MD simulations of the β-heptapeptide solvated in methanol and the target (derived from experiment) for the 39 bond-vector RDCs using different values of the parameters *K*^RDC,*mfv*^, , *N^mfv^*, *K*^RDC,*msy*^ and ; Table S2. Average of the absolute values of the restraining forces on the magnetic-field vector, <|*f^mfv^*|>, from the MD simulations of the β-heptapeptide solvated in methanol for the 39 bond-vector RDCs using different values of the parameters *K*^RDC,*mfv*^, , *N^mfv^*, *K*^RDC,*msy*^ and ; Table S3. Ratio of the occurrence of angles *θ_ab,H_* between the four vectors mentioned in Section 4.8 and the magnetic-field vector  around (±5.7°) the magic angles (55°,125°) and around 90° (±5.7°), averaged over the four vectors , from the MD simulations of the β-heptapeptide solvated in methanol using different values of the parameters *K*^RDC,*mfv*^, , *N^mfv^*, *K*^RDC,*msy*^ and ; Table S4. Average of the absolute values of the restraining forces on the molecule, <|*f^msy^*|>, from the MD simulations of the β-heptapeptide solvated in methanol for a set of 9 bond-vector RDCs (No 1, 3, 7, 11, 14, 22, 25, 35, 36) using different values of the parameters *K*^RDC,*mfv*^, , *N^mfv^*, *K*^RDC^,*^msy^,* and ; Table S5. List of 39 RDC values derived from experiment, , and the averages <*D_k_*> from unrestrained and RDC-restrained MD simulations of the ß-heptapeptide using different values of the parameters *K*^RDC,*mfv*^, , *Nmfv*, and *K*^RDC,*msy;*^ Table S6. Lists of 42^45^ and 119^24^ NOE atom–atom upper distance bounds derived from experiment and the corresponding *r^–6^* averaged distances from unrestrained and RDC-restrained MD simulations of the ß-heptapeptide using different values of the parameters *K*^RDC,*mfv*^, , *N*^mfv^, and *K*^RDC,*msy;*^ Table S7. List of 21 ^3^*J*-coupling constants derived from experiment and averaged from unrestrained and RDC-restrained MD simulations of the ß-heptapeptide using different values of the parameters *K*^RDC,*mfv*^, , *N*^mfv^, and *K*^RDC,*msy;*^ Table S8. Average occurrence of the three hydrogen bonds NH(2)–O(4), NH(3)–O(5), and NH(4)–O(6) (characterizing the left-handed M-314-helical fold) in the MD simulations of the β-heptapeptide solvated in methanol using different values of the parameters *K*^RDC,*mfv*^, , *N^mfv^*, *K*^RDC,msy^, and ; Table S9. Intramolecular M-3_14_-helical hydrogen bonds (%) from unrestrained and RDC-restrained MD simulations of the ß-heptapeptide using different values of the parameters *K*^RDC,*mfv*^, , *N*^mfv^, and *K*^RDC,msy^; Table S10. List of 39 RDC values derived from experiment, , and the averages <*D_k_*> from MD simulations of the ß-heptapeptide solvated in methanol with RDC-restraining of the magnetic-field vector and the molecule (*HRSrMDsol*) using four different (sub)sets of target RDCs. Table S11. Averages and root-mean-square fluctuations (*RMSF*) of 19 backbone and 6 side-chain torsional angles (degree) from MD simulations of the ß-heptapeptide solvated in methanol without any restraining of the molecule using four different (sub)sets of RDCs; Table S12. List of 39 RDC values derived from experiment, , and the averages <*D_k_*> from unrestrained and RDC-restrained (using the *HRS* and *AT* methods) MD or SD simulations of the ß-heptapeptide solvated in methanol (MD) or in vacuo (SD); Table S13. Intramolecular M-3_14_-helical hydrogen bonds from unrestrained and RDC-restrained (using the *HRS* and *AT* methods) MD or SD simulations of the ß-heptapeptide solvated in methanol (MD) or in vacuo (SD). Table S14. Lists of 42^45^ and 119^24^ NOE atom–atom upper distance bounds derived from experiment and the corresponding *r^–6^* averaged distances from unrestrained and RDC-restrained (using the *HRS* and *AT* methods) MD or SD simulations of the ß-heptapeptide solvated in methanol (MD) or in vacuo (SD); Table S15. List of 21 ^3^*J*-coupling constants derived from experiment and averaged from unrestrained and RDC-restrained (using the *HRS* and *AT* methods) MD or SD simulations of the ß-heptapeptide solvated in methanol (MD) or in vacuo (SD); Table S16. Averages and root-mean-square fluctuations (*RMSF*) of 19 backbone and 6 side-chain torsional angles (degree) from unrestrained and RDC-restrained (using the*HRS*and *AT* methods) MD or SD simulations of the ß-heptapeptide solvated in methanol (MD) or in vacuo (SD); Figure S1. Distribution of the angle  between 10 RDC-vectors for  and the magnetic-field direction  from MD simulations of the ß-heptapeptide solvated in methanol without any restraining of the molecule with , using different values of the parameters *N^mfv^* and*K*^RDC,mfv^; Figure S2. Distribution of the angle  between 10 RDC-vectors for  and the magnetic-field direction  from MD simulations of the ß-heptapeptide solvated in methanol without any restraining of the molecule with  using different values of the parameters *N^mfv^* and *K*^RDC,*mfv*^; Figure S2. Distribution of the angle  between 10 RDC-vectors for  and the magnetic-field direction  from MD simulations of the ß-heptapeptide solvated in methanol without any restraining of the molecule with  = 100 ns, using different values of the parameters *N*_*mfv*_ and *K*^RDC,*mfv*^(PDF)
