# Correction to “Molecular Structure Refinement of a β‑Heptapeptide Based on Residual Dipolar Couplings: The Challenge of Extracting Structural Information from Measured RDCs”

**DOI:** 10.1021/acs.jpcb.5c03491

**Published:** 2025-06-06

**Authors:** Maria Pechlaner, Wilfred F. van Gunsteren, Lorna J. Smith, Niels Hansen

Equations 24a and 24b of ref 1

$$\beta_{1}\left(t\right)=\left(1/2\right)\arccos\left(4a_{1}\left(t\right)/3\right),$$

$$\beta_{2}\left(t\right)=\left(1/2\right)\arccos\left(4a_{2}\left(t\right)/3\right),$$

are not correct; a term −1/3 is missing in the argument of the arccos function. These wrong equations were used to obtain the angular distributions in Figure 6 of ref 2.

As a consequence, the lines presented in Figure 6 of ref 2, which show the angle θ_*ab,H*_ between the vector ${\mathrm{r⃗}}_{ab}$ connecting the atoms *a* and *b* in the molecule and the vector representing the direction of the magnetic field H⃗, are incorrect. The lines have limited value, because they do not represent the orientation distribution of the molecule. Therefore, their calculation is not repeated. These findings have no consequences for the conclusions of the study.

It should be noted that eqs 36 and 37 in ref 2 should be eqs 21 and 22, respectively. There are no missing equations in ref 2.
