# Supplementary material for: Molecular Structure Refinement of a ß-Heptapeptide Based on Residual Dipolar Couplings: The Challenge of Extracting Structural Information from Measured RDCs
Source: J Phys Chem B. 2025 Mar 13;129(12):3131–58. doi: 10.1021/acs.jpcb.4c06955 (PMC11956018; doi:10.1021/acs.jpcb.4c06955)
Supplement: Supplementary file 1 — jp4c06955_si_001.pdf [file jp4c06955_si_001.pdf]

Molecular Structure Refinement of a Beta-Heptapeptide Based on Residual Dipolar Couplings: The Challenge of Extracting Structural Information from Measured RDCs

Supporting Information

Maria Pechlaner[#]*, Wilfred F. van Gunsteren[#], Lorna J. Smith[†], and Niels Hansen[&]

[#]Institute of Molecular Physical Science, Swiss Federal Institute of Technology, ETH, CH-8093 Zurich, Switzerland,

[†]Department of Chemistry, Inorganic Chemistry Laboratory, University of Oxford, South Parks Road, Oxford, OX1 3QR, UK,

[&]Institute of Thermodynamics and Thermal Process Engineering, University of Stuttgart, D-70569 Stuttgart, Germany

[*]Corresponding author. Electronic mail: maria.pechlaner@chem.ethz.ch

Version/Date: 09-12-2024

Tables S1 – S16

Table S1. Root-mean-square difference (*RMSD*, Hz) between the averages $< D_k >_{t^{msy}}$ from the MD simulations of the β-heptapeptide solvated in methanol and the target D_k^0 (derived from experiment) for the 39 bond-vector RDCs (Hz). $K^{RDC,msy}$ = 0: MD simulation without any restraining of the molecule (*MDsol*). $K^{RDC,msy}$ > 0: MD simulation with RDC-restraining of the molecule (*HRSrMDsol*). Parameter values of the t^{msy} = 100 ns *HRS* simulations are γ^{mfv} = 2.4 ps^{-1} and ΔD^{fb} = 2.0 Hz, while the values of the parameters $K^{RDC,mfv}$ (kJmol^{-1}Hz^{-2}), $\tau_\theta^{RDC,mfv}$ (ns), N_{mfv} , $K^{RDC,msy}$ (kJmol^{-1}Hz^{-2}) and $\tau_\theta^{RDC,msy}$ (ns) were varied. RDC-values D_k^0 from Tables 1 and 2 of Ref.[24]. *RMSD*-values of averaged RDC-values $< D_{k_1k_2} >_{t^{msy}}$ from their target values $D_{k_1k_2}^0$ larger than 2 Hz are in (red) italics. "*sf*": Result not available due to SHAKE failure: too large (restraining) forces.

$K^{RDC,mfv}$ (kJmol^{-1}Hz^{-2})	$\tau_\theta^{RDC,mfv}$ (ns)	N_{mfv}	$\tau_\theta^{RDC,msy}$ = 0.1 ns			$\tau_\theta^{RDC,msy}$ = 1 ns	
			$K^{RDC,msy}$ = 0	$K^{RDC,msy}$ = 0.05	$K^{RDC,msy}$ = 0.5	$K^{RDC,msy}$ = 0.05	$K^{RDC,msy}$ = 0.5
1	10	10	*5.7*	*sf*	*sf*	*10.0*	*sf*
		100	*3.0*	*4.7*	*sf*	*2.8*	*4.8*
		1000	*3.2*	*3.9*	*5.0*	*2.9*	*4.8*
	100	10	*11.2*			*10.6*	*sf*
		100	*5.8*			*6.1*	*sf*
		1000	*4.4*			*4.2*	*6.1*
10	10	10	1.4	1.5	*sf*	1.5	*sf*
		100	1.2	1.3	*sf*	1.2	1.7
		1000	1.7	1.6	*2.8*	1.6	*2.6*
	100	10	*3.1*	*sf*	*sf*	*4.8*	*sf*
		100	1.8	*2.4*	*sf*	1.5	*2.2*
		1000	1.9	2.0	*2.8*	1.9	*3.2*
100	10	10	0.5	0.4	*sf*	0.4	0.6
		100	0.5	0.6	0.7	0.6	0.6
		1000	1.5	1.5		1.4	1.5
	100	10	0.6			0.8	*sf*
		100	0.6			0.6	0.6
		1000	0.7			0.7	0.6
1000	10	10	0.5	0.4	*sf*		
		100	0.7	0.7	0.8		

Table S2. Average of the absolute values of the restraining forces on the magnetic-field vector ($kJmol^{-1}nm^{-1}$), $<|f^{mfv}|>$, from the MD simulations of the β-heptapeptide solvated in methanol for the 39 bond-vector RDCs (Hz). $K^{RDC,msy}$ = 0: MD simulation without any restraining of the molecule (*MDsol*). $K^{RDC,msy}$ > 0: MD simulation with RDC-restraining of the molecule (*HRSrMDsol*). Parameter values of the t^{msy} = 100 ns *HRS* simulations are γ^{mfv} = 2.4 ps^{-1} and ΔD^{fb} = 2.0 Hz, while the values of the parameters $K^{RDC,mfv}$ ($kJmol^{-1}Hz^{-2}$), $\tau_\theta^{RDC,mfv}$ (ns), N_{mfv} , $K^{RDC,msy}$ ($kJmol^{-1}Hz^{-2}$) and $\tau_\theta^{RDC,msy}$ (ns) were varied. Target RDC-values D_k^0 from Tables 1 and 2 of Ref.[24]. $<|f^{mfv}|>$-values larger than 10 $kJmol^{-1}nm^{-1}$ are in (red) italics. "*sf*": Result not available due to SHAKE failure: too large (restraining) forces.

$K^{RDC,mfv}$ ($kJmol^{-1}Hz^{-2}$)	$\tau_\theta^{RDC,mfv}$ (ns)	N_{mfv}	$\tau_\theta^{RDC,msy}$ = 0.1 ns			$\tau_\theta^{RDC,msy}$ = 1 ns	
			$K^{RDC,msy}$ = 0	$K^{RDC,msy}$ = 0.05	$K^{RDC,msy}$ = 0.5	$K^{RDC,msy}$ = 0.05	$K^{RDC,msy}$ = 0.5
1	10	10	0.7	*sf*	*sf*	0.5	*sf*
		100	0.7	0.6	*sf*	0.7	0.6
		1000	0.8	0.8	0.7	0.8	0.8
	100	10	0.1			0.0	*sf*
		100	0.1			0.1	*sf*
		1000	0.1			0.1	0.1
10	10	10	4.7	4.4	*sf*	4.6	*sf*
		100	4.8	4.7	*sf*	4.8	4.7
		1000	5.1	6.5		5.1	5.1
	100	10	0.5	*sf*	*sf*	0.4	*sf*
		100	0.5	0.5	*sf*	0.5	0.4
		1000	0.6	0.5	0.5	0.6	0.5
100	10	10	*35.8*	*35.4*	*sf*	*35.8*	*35.1*
		100	*34.8*	*34.7*	*34.1*	*34.7*	*34.8*
		1000	*35.9*			*36.1*	*36.0*
	100	10	2.9			2.9	*sf*
		100	2.8			2.8	2.8
		1000	2.8			2.8	2.8
1000	10	10	*403.0*	*392.6*	*sf*		
		100	*373.2*	*374.3*	*358.0*		

Table S3. Ratio of the occurrence of angles $\theta_{ab,H}$ between the four vectors $\vec{r}_{ab}$ mentioned in section 4.8 and the magnetic-field vector $\vec{H}$ around (± 5.7°) the magic angles (55°,125°) and around 90° (± 5.7°), averaged over the four vectors $\vec{r}_{ab}$, from the MD simulations of the β-heptapeptide solvated in methanol. $K^{RDC,msy}$ = 0: MD simulation without any restraining of the molecule (*MDsol*). $K^{RDC,msy}$ > 0: MD simulation with RDC-restraining of the molecule (*HRSrMDsol*). Parameter values of the t^{msy} = 100 ns *HRS* simulations are γ^{mfv} = 2.4 ps^{-1} and ΔD^{fb} = 2.0 Hz, while the values of the parameters $K^{RDC,mfv}$ ($kJmol^{-1}Hz^{-2}$), $\tau_{\theta}^{RDC,mfv}$ (ns), N_{mfv}, $K^{RDC,msy}$ ($kJmol^{-1}Hz^{-2}$) and $\tau_{\theta}^{RDC,msy}$ (ns) were varied. Target RDC-values D_k^0 from Tables 1 and 2 of Ref.[24]. Ratios larger than 0.9 are in (red) italics. "*sf*": Result not available due to SHAKE failure: too large (restraining) forces.

$K^{RDC,mfv}$ ($kJmol^{-1}Hz^{-2}$)	$\tau_{\theta}^{RDC,mfv}$ (ns)	N_{mfv}	$\tau_{\theta}^{RDC,msy}$ = 0.1 ns			$\tau_{\theta}^{RDC,msy}$ = 1 ns	
			$K^{RDC,msy}$ = 0	$K^{RDC,msy}$ = 0.05	$K^{RDC,msy}$ = 0.5	$K^{RDC,msy}$ = 0.05	$K^{RDC,msy}$ = 0.5
1	10	10	0.45	*sf*	*sf*	0.63	*sf*
		100	0.07	0.12	*sf*	0.27	0.24
		1000	0.12	0.13	0.06	0.12	0.06
	100	10	0.33			0.39	*sf*
		100	0.28			0.15	*sf*
		1000	0.10			0.12	0.05
10	10	10	0.65	0.65	*sf*	0.36	*sf*
		100	0.12	0.22	*sf*	0.28	0.15
		1000	0.21	0.21	0.07	0.17	0.04
	100	10	0.38	*sf*	*sf*	0.43	*sf*
		100	0.25	0.19	*sf*	0.12	0.16
		1000	0.14	0.09	0.06	0.14	0.04
100	10	10	*2.53*	*1.76*	*sf*	*2.81*	*1.48*
		100	*2.13*	*2.28*	*0.94*	*2.15*	*2.28*
		1000	*2.28*	*2.65*		*2.73*	*2.64*
	100	10	0.75			0.40	*sf*
		100	0.10			0.14	0.25
		1000	0.15			0.13	0.16
1000	10	10	*10.62*	*7.27*	*sf*		
		100	*6.82*	*8.10*	*1.43*		

Table S4. Average of the absolute values of the restraining forces on the molecule ($kJmol^{-1}nm^{-1}$), $<|f^{msy}|>$, from the MD simulations of the β-heptapeptide solvated in methanol for a set of 9 bond-vector RDCs (No 1, 3, 7, 11, 14, 22, 25, 35, 36). $K^{RDC,msy} = 0$: MD simulation without any restraining of the molecule (*MDsol*). $K^{RDC,msy} > 0$: MD simulation with RDC-restraining of the molecule (*HRSrMDsol*). Parameter values of the $t^{msy} = 100$ ns *HRS* simulations are $\gamma^{mfv} = 2.4\ ps^{-1}$ and $\Delta D^{fb} = 2.0$ Hz, while the values of the parameters $K^{RDC,mfv}$ ($kJmol^{-1}Hz^{-2}$), $\tau_{\theta}^{RDC,mfv}$ (ns), N_{mfv} , $K^{RDC,msy}$ ($kJmol^{-1}Hz^{-2}$) and $\tau_{\theta}^{RDC,msy}$ (ns) were varied. Target RDC-values D_k^0 from Tables 1 and 2 of Ref.[24]. "*sf*": Result not available due to SHAKE failure: too large (restraining) forces.

$K^{RDC,mfv}$ ($kJmol^{-1}Hz^{-2}$)	$\tau_{\theta}^{RDC,mfv}$ (ns)	N_{mfv}	$\tau_{\theta}^{RDC,msy}$ = 0.1 ns			$\tau_{\theta}^{RDC,msy}$ = 1 ns	
			$K^{RDC,msy}$ = 0	$K^{RDC,msy}$ = 0.05	$K^{RDC,msy}$ = 0.5	$K^{RDC,msy}$ = 0.05	$K^{RDC,msy}$ = 0.5
1	10	10	0.0	*sf*	*sf*	75.5	*sf*
		100	0.0	62.9	*sf*	12.7	165.3
		1000	0.0	10.3	120.7	2.3	26.9
	100	10	0.0			105.9	*sf*
		100	0.0			27.9	*sf*
		1000	0.0			5.1	67.8
10	10	10	0.0	148.0	*sf*	18.6	*sf*
		100	0.0	16.9	*sf*	3.3	32.8
		1000	0.0	2.6	24.5	0.3	14.1
	100	10	0.0	*sf*	*sf*	82.1	*sf*
		100	0.0	79.4	*sf*	11.3	142.5
		1000	0.0	10.0	121.3	1.4	22.1
100	10	10	0.0	40.0	*sf*	5.1	53.1
		100	0.0	5.1	50.7	0.1	0.6
		1000	0.0			0.0	0.6
	100	10	0.0			27.3	*sf*
		100	0.0			3.3	34.8
		1000	0.0			0.0	0.1
1000	10	10	0.0	24.0	*sf*		
		100	0.0	3.3	32.7		

Table S5. List of 39 RDC-values (Hz) derived from experiment, D_k^0, and the averages $<D_k>$ from unrestrained and RDC-restrained MD simulations of the ß-heptapeptide. *MDsol*: MD simulation of the peptide solvated in methanol without any restraining of the molecule ($K^{RDC,msy}$ = 0 kJmol^{-1}Hz^{-2}). *HRSrMDsol*: RDC-restraining MD simulations with $K^{RDC,msy}$ = 0.05 kJmol^{-1}Hz^{-2} in which the parameters $K^{RDC,mfv}$, $\tau_\theta^{RDC,mfv}$ and N_{mfv} were varied. Other parameter values of the t^{msy} = 100 ns *HRS* simulations are γ^{mfv} = 2.4 ps^{-1}, ΔD^{fb} = 2.0 Hz, $\tau_\theta^{RDC,msy}$ = 1 ns. *RMSD*: root-mean-square difference between $< D_{k_1k_2} >_{t^{msy}}$ and $D_{k_1k_2}^0$ for the 39 bond-vector RDCs. The residue sequence numbers of the atoms are within parentheses. RDC-values D_k^0 from Tables 1 and 2 of Ref.[24]. Deviations of averaged RDC-values $< D_{k_1k_2} >_{t^{msy}}$ from their target values $D_{k_1k_2}^0$ larger than 2 Hz are in (red) italics.

RDC sequence number	RDC atoms		D_k^0 (Hz)	$< D_{k_1k_2} >_{t^{msy}}$ (Hz)								
Simulation				*MDsol*	*HRSMDsol*							
$K^{RDC,mfv}$	kJmol^{-1}Hz^{-2}			100	10	10	10	10	10	100	100	100
$\tau_\theta^{RDC,mfv}$	ns			100	10	10	10	100	100	100	100	100
N_{mfv}				100	10	100	1000	100	1000	10	100	1000
$K^{RDC,msy}$	kJmol^{-1}Hz^{-2}			0	0.05	0.05	0.05	0.05	0.05	0.05	0.05	0.05
1	^{1}H(2)	^{15}N(2)	3.4	3.5	*5.9*	3.2	3.2	2.2	3.4	4.3	3.7	3.2
2	^{1}H(3)	^{15}N(3)	5.6	5.3	6.1	5.0	4.5	5.1	3.9	6.3	5.3	5.2
3	^{1}H(4)	^{15}N(4)	4.4	4.5	5.0	3.9	4.3	4.1	4.6	5.0	4.8	4.4
4	^{1}H(5)	^{15}N(5)	4.5	4.3	3.5	3.1	3.4	4.1	3.4	5.0	4.5	4.0
5	^{1}H(6)	^{15}N(6)	5.2	4.9	3.3	4.9	4.1	3.3	3.6	4.3	5.0	4.6
6	^{1}H(7)	^{15}N(7)	4.7	4.6	*2.6*	3.9	3.7	3.8	3.0	5.8	4.1	4.2
7	^{1}H$_ß$(1)	^{13}C$_ß$(1)	-6.9	-6.5	-7.9	-6.7	-5.6	-6.4	-5.7	-7.2	-6.5	-6.6

8	$^{1}H_{\beta}(2)$	$^{13}C_{\beta}(2)$	-12.3	-12.3	-10.3	-11.1	-10.6	-11.4	-10.3	-11.4	-12.2	-11.8
9	$^{1}H_{\beta}(3)$	$^{13}C_{\beta}(3)$	-17.1	-15.3	*-14.1*	*-13.8*	*-12.1*	*-14.2*	*-12.5*	-15.6	-15.4	-15.1
10	$^{1}H_{\beta}(4)$	$^{13}C_{\beta}(4)$	-12.0	-12.1	*-14.5*	-12.0	-11.4	-13.0	-11.7	-10.9	-11.9	-12.0
11	$^{1}H_{\beta}(5)$	$^{13}C_{\beta}(5)$	-11.3	-11.1	-9.8	-11.0	-10.0	-9.8	-10.0	-10.2	-11.2	-10.9
12	$^{1}H_{\beta}(6)$	$^{13}C_{\beta}(6)$	-12.6	-12.1	-12.8	-12.2	-11.3	-11.5	-11.2	-11.8	-12.6	-12.3
13	$^{1}H_{\beta}(7)$	$^{13}C_{\beta}(7)$	-12.8	-11.7	*-10.4*	*-10.0*	*-9.3*	-11.1	*-9.5*	-11.5	-12.0	-11.2
14	$^{1}H_{\alpha Re}(1)$	$^{13}C_{\alpha}(1)$	-6.2	-6.4	-4.4	-6.5	-6.0	-5.9	-5.4	-5.0	-6.4	-6.2
15	$^{1}H_{\alpha Re}(2)$	$^{13}C_{\alpha}(2)$	-11.0	-10.6	-9.7	-11.0	-10.1	-10.8	-9.9	-10.4	-11.0	-10.8
16	$^{1}H_{\alpha Re}(4)$	$^{13}C_{\alpha}(4)$	-12.5	-12.5	-12.8	-12.4	-11.5	-12.8	-12.0	-12.2	-12.3	-12.4
17	$^{1}H_{\alpha Re}(6)$	$^{13}C_{\alpha}(6)$	-8.6	-9.7	-9.9	-9.5	-9.0	-10.2	-9.7	-8.0	-9.6	-9.4
18	$^{1}H_{\alpha Re}(7)$	$^{13}C_{\alpha}(7)$	-8.7	-8.5	-8.8	-7.6	-7.0	*-5.8*	*-6.3*	-9.3	-8.3	-8.0
19	$^{1}H_{\alpha Si}(1)$	$^{13}C_{\alpha}(1)$	1.5	1.4	1.8	1.0	1.3	1.9	1.0	1.5	1.3	1.5
20	$^{1}H_{\alpha Si}(2)$	$^{13}C_{\alpha}(2)$	6.9	7.1	6.8	7.0	7.2	6.9	7.3	5.3	7.0	7.3
21	$^{1}H_{\alpha Si}(5)$	$^{13}C_{\alpha}(5)$	10.1	9.7	8.1	8.8	8.4	*7.5*	*7.9*	9.2	9.5	9.5
22	$^{1}H_{\alpha Si}(6)$	$^{13}C_{\alpha}(6)$	7.1	6.1	*5.0*	5.4	5.4	5.8	5.2	6.8	6.1	6.0
23	$^{1}H_{\alpha Si}(7)$	$^{13}C_{\alpha}(7)$	2.2	2.4	1.3	2.4	3.0	4.2	3.5	1.8	2.6	2.8
24	$^{1}H_{\alpha Re}(1)$	$^{1}H_{\alpha Si}(1)$	-1.5	-1.2	0.4	-1.1	-1.0	-0.4	-0.4	-1.7	-1.1	-1.1
25	$^{1}H_{\alpha Re}(2)$	$^{1}H_{\alpha Si}(2)$	1.1	0.6	*-1.0*	0.5	0.1	0.8	0.7	1.6	0.6	0.7
26	$^{1}H_{\alpha Re}(3)$	$^{1}H_{\alpha Si}(3)$	-0.9	-1.4	-1.8	-1.9	-1.9	-1.4	-2.1	-1.0	-1.3	-1.3
27	$^{1}H_{\alpha Re}(5)$	$^{1}H_{\alpha Si}(5)$	2.4	2.5	1.7	3.0	2.1	1.5	1.9	1.5	2.9	2.5
28	$^{1}H_{\alpha Re}(6)$	$^{1}H_{\alpha Si}(6)$	-0.2	0.7	1.7	0.8	0.6	0.0	0.6	-0.5	1.0	0.7
29	$^{1}H_{\alpha Re}(7)$	$^{1}H_{\alpha Si}(7)$	-2.0	-2.1	-2.4	-2.8	-2.2	-1.7	-1.4	-1.7	-2.3	-2.4
30	$^{1}H_{\gamma}(1)$	$^{13}C_{\gamma}(1)$	-1.2	-1.4	-1.4	-0.8	-1.6	-2.1	-1.2	-1.0	-1.0	-1.3
31	$^{1}H_{\gamma Re}(3)$	$^{13}C_{\gamma}(3)$	-2.9	-3.3	-3.4	-2.1	-2.2	-1.8	-1.2	-3.3	-3.2	-3.1
32	$^{1}H_{\gamma Si}(3)$	$^{13}C_{\gamma}(3)$	4.1	2.8	*1.3*	*1.3*	*-0.7*	*-0.6*	-2.1	4.2	2.4	2.3
33	$^{1}H_{\gamma Re}(3)$	$^{1}H_{\gamma Si}(3)$	0.8	1.1	1.4	0.2	-0.7	1.3	-0.8	0.4	1.4	1.0
34	$^{1}H_{\delta}(3)$	$^{13}C_{\delta}(3)$	6.0	5.9	6.1	*3.7*	4.0	4.8	4.0	5.5	5.6	5.4
35	$^{1}H_{\gamma}(5)$	$^{13}C_{\gamma}(5)$	-4.1	-4.2	-5.0	-3.6	-3.6	-2.1	-3.0	-3.9	-3.9	-4.1
36	$^{1}H_{\gamma Re}(7)$	$^{13}C_{\gamma}(7)$	-3.6	-4.0	-3.7	-3.4	-3.4	-4.4	-2.8	-4.2	-3.7	-3.6
37	$^{1}H_{\gamma Si}(7)$	$^{13}C_{\gamma}(7)$	2.3	1.6	1.5	0.5	0.4	*-0.1*	*-0.3*	1.9	1.9	1.6
38	$^{1}H_{\gamma Re}(7)$	$^{1}H_{\gamma Si}(7)$	-1.4	-1.1	-1.5	-1.9	-1.7	-1.9	-1.4	-1.0	-1.0	-1.2
39	$^{1}H_{\delta}(7)$	$^{13}C_{\delta}(7)$	6.0	5.8	*3.6*	4.6	*3.9*	4.5	4.1	4.8	5.7	5.5

RMSD				0.6	1.5	1.2	1.6	1.5	1.9	0.8	0.6	0.7

Table S6. Lists of 42[45] and 119[24] NOE atom-atom upper distance (nm) bounds derived from experiment and the corresponding r^{-6} averaged distances (in nm) from unrestrained and RDC-restrained MD simulations of the ß-heptapeptide. *MDsol*: MD simulation of the peptide solvated in methanol without any restraining of the molecule ($K^{RDC,msy}$ = 0 kJmol^{-1}Hz^{-2}). *HRSrMDsol*: RDC-restraining MD simulations with $K^{RDC,msy}$ = 0.05 kJmol^{-1}Hz^{-2} in which the parameters $K^{RDC,mfv}$, $\tau_{\theta}^{RDC,mfv}$ and N_{mfv} were varied. Other parameter values of the t^{msy} = 100 ns *HRS* simulations are γ^{mfv} = 2.4 ps^{-1}, ΔD^{fb} = 2.0 Hz, $\tau_{\theta}^{RDC,msy}$ = 1 ns. *NOE distance upper bounds*: 42 from Table 1 of Ref.[45] and 119 (excluding five NOE bounds involving an "HT" atom) from Table S2 of Supplementary Information of Ref.[24]. *RMSD42*: root-mean-square deviations of the r^{-6} averaged distances beyond the 42 NOE upper bounds of Ref.[45]. *Nviol42*: number of distance-bound violations larger than 0.1 nm. *RMSD119*: root-mean-square deviations of the r^{-6} averaged distances beyond the 119 NOE upper bounds of Ref.[24]. *Nviol119*: number of distance-bound violations larger than 0.1 nm. The residue sequence numbers of the atoms are within parentheses. Values 0.1 nm larger than the largest experimentally derived NOE upper bound value are in (red) italics. "Me": three H-atoms of a methyl group. The three hydrogens of the methyl group attached to the C_{α}(4)-atom, denoted as Me-C_{α}(4) in Ref.[45], and as Hδ*(4) in Ref.[24], are denoted as Me-C_{δ}(4).

NOE sequence number	NOE H-atom pair	NOE distance upper bound (nm)		r^{-6} averaged distance (nm)								
Simulation		Ref. [45]	Ref. [24]	*MDsol*	*HRSrMDsol*							
$K^{RDC,mfv}$	kJmol^{-1}Hz^{-2}				10	10	10	10	10	100	100	100
$\tau_{\theta}^{RDC,mfv}$	ns				10	10	10	100	100	100	100	100
N_{mfv}					10	100	1000	100	1000	10	100	1000
$K^{RDC,msy}$	kJmol^{-1}Hz^{-2}			0	0.05	0.05	0.05	0.05	0.05	0.05	0.05	0.05

1	H-N(1)	H-C_{β}(1)	0.28	-	0.26	0.26	0.26	0.26	0.26	0.26	0.26	0.26	0.25
2	H-C_{β}(1)	H_{Re}-C_{α}(1)	0.29	0.28	0.28	0.28	0.28	0.28	0.28	0.28	0.27	0.28	0.28
3	H-C_{β}(1)	H_{Si}-C_{α}(1)	0.30	0.24	0.24	0.25	0.24	0.24	0.25	0.24	0.25	0.24	0.24
4	H-N(2)	H_{Re}-C_{α}(1)	0.24	0.26	0.24	0.26	0.24	0.24	0.24	0.24	0.26	0.24	0.24
5	H-N(2)	H_{Si}-C_{α}(1)	0.29	0.33	0.25	0.24	0.25	0.25	0.25	0.25	0.23	0.25	0.25
6	H-N(2)	H-C_{β}(2)	0.33	-	0.28	0.28	0.28	0.28	0.28	0.28	0.28	0.28	0.28
7	H-N(2)	H-C_{β}(4)	0.35	0.33	0.36	0.42	0.36	0.36	0.36	0.36	*0.48*	0.36	0.36
8	H-N(2)	H-C_{β}(5)	0.33	0.31	0.31	0.35	0.31	0.31	0.31	0.30	0.41	0.30	0.30
9	H-C_{β}(2)	Me-C_{γ}(2)	0.32	0.38	0.24	0.24	0.24	0.24	0.24	0.24	0.24	0.24	0.24
10	H-C_{β}(2)	H_{Si}-C_{α}(2)	0.23	0.23	0.24	0.25	0.24	0.24	0.24	0.24	0.25	0.24	0.24
11	H-N(3)	H_{Re}-C_{α}(2)	0.22	0.24	0.21	0.23	0.21	0.21	0.22	0.21	0.25	0.22	0.21
12	H-N(3)	H_{Si}-C_{α}(2)	0.31	-	0.30	0.25	0.30	0.30	0.29	0.30	0.24	0.28	0.30
13	H-N(3)	H-C_{β}(3)	0.31	-	0.29	0.28	0.28	0.29	0.28	0.29	0.28	0.29	0.29
14	H-N(3)	H_{Re}-C_{α}(3)	0.26	0.29	0.24	0.25	0.25	0.25	0.24	0.25	0.25	0.24	0.25
15	H-N(3)	H-N(4)	0.38	-	0.38	0.38	0.38	0.37	0.37	0.37	0.38	0.37	0.37
16	H-N(3)	H-C_{β}(5)	0.34	0.34	0.36	0.41	0.36	0.36	0.35	0.35	*0.48*	0.35	0.35
17	H-N(3)	H-C_{β}(6)	0.32	0.31	0.30	0.35	0.30	0.30	0.30	0.30	0.41	0.30	0.30
18	H-C_{β}(3)	H-C_{δ}(3)	0.30	-	0.27	0.27	0.27	0.27	0.27	0.27	0.27	0.27	0.27
19	H-N(4)	H_{Re}-C_{α}(3)	0.23	0.24	0.22	0.23	0.22	0.22	0.22	0.21	0.24	0.21	0.21
20	H-N(4)	H_{Si}-C_{α}(3)	0.28	0.44	0.28	0.25	0.28	0.28	0.29	0.29	0.25	0.29	0.29
21	H-N(4)	H-C_{β}(4)	0.29	-	0.29	0.28	0.29	0.29	0.29	0.29	0.28	0.29	0.29
22	H-N(4)	Me-C_{γ}(4)	0.40	0.48	0.29	0.29	0.29	0.29	0.29	0.29	0.29	0.29	0.29
23	H-N(4)	H-C_{β}(6)	0.32	0.33	0.36	0.41	0.37	0.36	0.36	0.36	*0.47*	0.36	0.36
24	H-N(4)	H-C_{β}(7)	0.37	0.31	0.30	0.35	0.30	0.30	0.30	0.29	0.41	0.29	0.29
25	H-C_{β}(4)	H_{Re}-C_{α}(1)	0.26	0.23	0.25	0.30	0.25	0.25	0.26	0.25	0.35	0.25	0.25
26	H-N(5)	H-N(4)	0.37	-	0.38	0.38	0.37	0.38	0.38	0.38	0.40	0.38	0.38
27	H-N(5)	H_{Re}-C_{α}(4)	0.22	0.23	0.22	0.22	0.22	0.22	0.22	0.22	0.23	0.22	0.22
28	H-N(5)	Me-C_{δ}(4)	0.45	0.52	0.35	0.34	0.35	0.35	0.35	0.35	0.34	0.35	0.35
29	H-N(5)	H-C_{β}(5)	0.35	-	0.28	0.28	0.28	0.28	0.28	0.28	0.27	0.28	0.28
30	H-N(5)	H_{Re}-C_{α}(5)	0.25	0.28	0.25	0.25	0.25	0.25	0.25	0.25	0.25	0.25	0.25
31	H-N(5)	H-N(6)	0.35	-	0.40	0.41	0.40	0.39	0.39	0.39	0.41	0.39	0.40
32	H-C_{β}(5)	H_{Re}-C_{α}(2)	0.23	0.23	0.26	0.30	0.26	0.26	0.26	0.25	*0.35*	0.26	0.25

33	H-$C_ß$(5)	H-C_γ(5)	0.26	0.25	0.25	0.25	0.25	0.25	0.25	0.25	0.25	0.25	0.25
34	H-$C_ß$(5)	H_{Si}-C_α(5)	0.25	0.23	0.24	0.24	0.24	0.24	0.24	0.24	0.24	0.24	0.24
35	H-N(6)	H-$C_ß$(6)	0.29	-	0.28	0.28	0.28	0.28	0.28	0.28	0.28	0.28	0.28
36	H-N(6)	H_{Re}-C_α(6)	0.25	0.27	0.24	0.25	0.25	0.25	0.25	0.24	0.26	0.24	0.24
37	H-N(6)	H_{Re}-C_α(5)	0.22	0.25	0.23	0.24	0.23	0.22	0.22	0.22	0.25	0.22	0.22
38	H-$C_ß$(6)	H_{Re}-C_α(3)	0.25	0.23	0.25	0.29	0.25	0.25	0.25	0.25	0.34	0.25	*0.25*
39	H-$C_ß$(6)	H_{Si}-C_α(6)	0.26	0.23	0.24	0.25	0.24	0.24	0.24	0.24	0.25	0.24	0.24
40	H-N(7)	H_{Re}-C_α(6)	0.24	0.26	0.24	0.24	0.24	0.24	0.23	0.24	0.24	0.24	0.24
41	H-N(7)	H-$C_ß$(7)	0.30	-	0.28	0.28	0.28	0.28	0.28	0.28	0.28	0.28	0.28
42	H-N(7)	H_{Re}-C_α(7)	0.27	0.27	0.25	0.25	0.25	0.25	0.25	0.25	0.26	0.25	0.25
43	H-C_γ(1)	H_{Si}-C_α(1)	-	0.27	0.26	0.25	0.25	0.26	0.25	0.25	0.26	0.26	0.26
44	H-C_γ(1)	H_{Re}-C_α(1)	-	0.31	0.30	0.30	0.30	0.31	0.30	0.30	0.30	0.30	0.30
45	H-$C_ß$(1)	H-C_γ(1)	-	0.24	0.24	0.24	0.24	0.24	0.24	0.24	0.24	0.24	0.24
46	H_{Si}-C_α(2)	Me-C_γ(2)	-	0.41	0.29	0.28	0.29	0.29	0.29	0.29	0.28	0.29	0.29
47	H_{Re}-C_α(2)	Me-C_γ(2)	-	0.41	0.29	0.30	0.29	0.29	0.29	0.29	0.31	0.29	0.29
48	H_{Re}-C_α(2)	H-N(2)	-	0.28	0.25	0.25	0.25	0.25	0.25	0.25	0.25	0.25	0.25
49	H-$C_ß$(2)	H_{Re}-C_α(2)	-	0.31	0.29	0.27	0.29	0.29	0.29	0.29	0.26	0.28	0.29
50	Me-C_γ(2)	H-N(2)	-	0.51	0.30	0.30	0.30	0.30	0.30	0.30	0.29	0.30	0.30
51	H_{Si}-C_α(3)	H-$C_ß$(3)	-	0.23	0.24	0.24	0.24	0.24	0.24	0.24	0.25	0.24	0.24
52	H_{Si}-C_α(3)	H-C_δ(3)	-	0.29	0.23	0.24	0.23	0.23	0.23	0.23	0.25	0.22	0.23
53	H_{Si}-C_α(3)	H_{Si}-C_γ(3)	-	0.27	0.33	0.30	0.33	0.34	0.34	0.34	0.29	0.34	0.34
54	H_{Si}-C_α(3)	Me-$C_{\varepsilon 2}$(3)	-	0.54	0.42	0.43	0.43	0.43	0.42	0.43	0.44	0.42	0.43
55	Me-$C_{\varepsilon 1}$(3)	H-$C_ß$(3)	-	0.41	0.31	0.32	0.31	0.31	0.31	0.31	0.33	0.30	0.31
56	H-$C_ß$(3)	Me-$C_{\varepsilon 2}$(3)	-	0.47	0.35	0.33	0.35	0.35	0.36	0.35	0.32	0.36	0.36
57	H-$C_ß$(3)	H_{Re}-C_γ(3)	-	0.31	0.27	0.26	0.27	0.27	0.27	0.27	0.26	0.27	0.27
58	H-$C_ß$(3)	H_{Si}-C_γ(3)	-	0.27	0.25	0.26	0.25	0.25	0.25	0.25	0.26	0.25	0.25
59	H-C_δ(3)	H-N(3)	-	0.38	0.37	0.33	0.36	0.37	0.37	0.38	0.31	0.38	0.38
60	Me-$C_{\varepsilon 1}$(3)	H_{Si}-C_γ(3)	-	0.42	0.28	0.28	0.28	0.28	0.28	0.28	0.28	0.28	0.28
61	H_{Re}-C_γ(3)	H_{Si}-C_α(3)	-	0.35	0.26	0.25	0.26	0.26	0.26	0.27	0.25	0.27	0.27
62	H_{Re}-C_γ(3)	H_{Re}-C_α(3)	-	0.26	0.24	0.26	0.24	0.24	0.24	0.24	0.27	0.24	0.24
63	H_{Re}-C_γ(3)	H-C_δ(3)	-	0.24	0.25	0.26	0.25	0.25	0.25	0.25	0.26	0.25	0.25
64	H_{Si}-C_γ(3)	H-C_δ(3)	-	0.26	0.27	0.26	0.27	0.27	0.27	0.27	0.26	0.27	0.27

65	H_{Re}-C_{γ}(3)	Me-$C_{\varepsilon 1}$(3)	-	0.47	0.34	0.32	0.33	0.34	0.34	0.34	0.32	0.34	0.34
66	H_{Re}-C_{γ}(3)	H-N(3)	-	0.34	0.28	0.29	0.28	0.28	0.28	0.28	0.30	0.28	0.28
67	H_{Si}-C_{γ}(3)	H_{Re}-C_{α}(3)	-	0.30	0.30	0.30	0.29	0.30	0.29	0.29	0.30	0.30	0.29
68	H_{Si}-C_{γ}(3)	H-N(3)	-	0.43	0.27	0.26	0.27	0.27	0.27	0.28	0.25	0.28	0.28
69	H_{Re}-C_{α}(4)	Me-C_{γ}(4)	-	0.40	0.28	0.29	0.28	0.28	0.28	0.28	0.30	0.28	0.28
70	H-N(4)	H_{Re}-C_{α}(4)	-	0.29	0.25	0.27	0.25	0.25	0.25	0.25	0.27	0.25	0.25
71	H-C_{β}(4)	H_{Re}-C_{α}(4)	-	0.28	0.28	0.25	0.28	0.28	0.28	0.28	0.24	0.28	0.28
72	H-C_{β}(4)	Me-C_{δ}(4)	-	0.40	0.29	0.29	0.29	0.29	0.29	0.29	0.30	0.29	0.29
73	H-C_{β}(4)	Me-C_{γ}(4)	-	0.38	0.24	0.24	0.24	0.24	0.24	0.24	0.24	0.24	0.24
74	H_{Si}-C_{α}(5)	Me-$C_{\delta 2}$(5)	-	0.41	0.33	0.32	0.33	0.33	0.32	0.33	0.32	0.33	0.33
75	H-C_{γ}(5)	H_{Si}-C_{α}(5)	-	0.28	0.26	0.27	0.26	0.27	0.26	0.26	0.26	0.26	0.26
76	H_{Re}-C_{α}(5)	Me-$C_{\delta 2}$(5)	-	0.45	0.34	0.35	0.34	0.34	0.34	0.34	0.35	0.34	0.34
77	H_{Re}-C_{α}(5)	H-C_{γ}(5)	-	0.28	0.27	0.28	0.28	0.28	0.28	0.27	0.29	0.27	0.27
78	H-C_{β}(5)	H_{Re}-C_{α}(5)	-	0.31	0.29	0.27	0.29	0.29	0.29	0.29	0.27	0.29	0.29
79	H-C_{β}(5)	Me-$C_{\delta 2}$(5)	-	0.39	0.30	0.30	0.30	0.30	0.30	0.30	0.30	0.30	0.30
80	Me-$C_{\delta 2}$(5)	H-N(5)	-	0.48	0.33	0.33	0.33	0.34	0.33	0.33	0.32	0.33	0.33
81	Me-$C_{\delta 1}$(5)	H-C_{γ}(5)	-	0.36	0.24	0.24	0.24	0.24	0.24	0.24	0.24	0.24	0.24
82	H-C_{γ}(5)	H-N(5)	-	0.32	0.28	0.28	0.28	0.28	0.29	0.28	0.28	0.28	0.28
83	H_{Si}-C_{α}(6)	Me-C_{γ}(6)	-	0.41	0.28	0.28	0.28	0.28	0.28	0.28	0.29	0.28	0.28
84	H_{Re}-C_{α}(6)	H-C_{β}(6)	-	0.31	0.28	0.27	0.28	0.28	0.28	0.28	0.26	0.28	0.28
85	Me-C_{γ}(6)	H_{Re}-C_{α}(6)	-	0.44	0.30	0.31	0.30	0.30	0.30	0.30	0.31	0.30	0.30
86	H-C_{β}(6)	Me-C_{γ}(6)	-	0.38	0.24	0.24	0.24	0.24	0.24	0.24	0.24	0.24	0.24
87	Me-C_{γ}(6)	H-N(6)	-	0.48	0.29	0.29	0.29	0.29	0.29	0.29	0.29	0.29	0.29
88	H_{Si}-C_{α}(7)	H-C_{β}(7)	-	0.25	0.25	0.25	0.25	0.25	0.24	0.24	0.25	0.25	0.25
89	H_{Si}-C_{α}(7)	H-C_{δ}(7)	-	0.27	0.23	0.25	0.23	0.23	0.23	0.23	0.25	0.23	0.23
90	H_{Si}-C_{γ}(7)	H_{Si}-C_{α}(7)	-	0.26	0.30	0.29	0.30	0.30	0.30	0.30	0.30	0.30	0.30
91	H_{Si}-C_{α}(7)	H-N(7)	-	0.35	0.32	0.31	0.32	0.33	0.32	0.33	0.31	0.32	0.32
92	H_{Re}-C_{γ}(7)	H_{Re}-C_{α}(7)	-	0.28	0.27	0.27	0.27	0.26	0.26	0.26	0.27	0.27	0.27
93	H_{Re}-C_{α}(7)	H_{Si}-C_{γ}(7)	-	0.28	0.31	0.31	0.31	0.31	0.31	0.31	0.31	0.31	0.31
94	H-C_{β}(7)	Me-$C_{\varepsilon 2}$(7)	-	0.39	0.33	0.32	0.33	0.33	0.34	0.34	0.32	0.33	0.33
95	H_{Re}-C_{γ}(7)	H-C_{β}(7)	-	0.29	0.26	0.26	0.26	0.26	0.26	0.26	0.26	0.26	0.26
96	H-C_{β}(7)	H_{Si}-C_{γ}(7)	-	0.26	0.26	0.26	0.26	0.26	0.26	0.26	0.26	0.26	0.26

97	H-C_{δ}(7)	H-N(7)	-	0.36	0.33	0.32	0.33	0.33	0.33	0.33	0.32	0.33	0.33
98	Me-$C_{\varepsilon 2}$(7)	H-N(7)	-	0.64	0.48	0.47	0.48	0.48	0.47	0.47	0.48	0.47	0.47
99	Me-$C_{\varepsilon 1}$(7)	H-C_{δ}(7)	-	0.35	0.24	0.24	0.24	0.24	0.24	0.24	0.24	0.24	0.24
100	Me-$C_{\varepsilon 2}$(7)	H_{Si}-C_{γ}(7)	-	0.42	0.32	0.32	0.32	0.32	0.32	0.32	0.32	0.32	0.32
101	Me-$C_{\varepsilon 1}$(7)	H_{Si}-C_{γ}(7)	-	0.41	0.28	0.28	0.28	0.28	0.28	0.28	0.28	0.28	0.28
102	Me-$C_{\varepsilon 1}$(7)	H_{Re}-C_{γ}(7)	-	0.39	0.33	0.32	0.33	0.33	0.33	0.33	0.32	0.33	0.33
103	H_{Re}-C_{γ}(7)	H-N(7)	-	0.32	0.28	0.29	0.28	0.28	0.28	0.28	0.29	0.28	0.28
104	H_{Si}-C_{γ}(7)	H-N(7)	-	0.39	0.25	0.25	0.26	0.25	0.26	0.25	0.25	0.25	0.25
105	H-C_{β}(1)	H-N(2)	-	0.48	0.42	0.38	0.42	0.41	0.41	0.42	0.37	0.41	0.42
106	H-C_{β}(2)	H-C_{δ}(3)	-	0.44	*0.65*	0.46	*0.64*	*0.61*	*0.63*	*0.65*	0.42	*0.62*	*0.65*
107	H-C_{β}(2)	H-N(3)	-	0.41	0.43	0.34	0.43	0.40	0.42	0.44	0.32	0.42	0.44
108	H-N(2)	H-C_{β}(3)	-	0.45	0.48	0.51	0.48	0.48	0.48	0.47	0.55	0.48	0.48
109	H-C_{β}(4)	H-N(5)	-	0.42	0.44	0.33	0.44	0.44	0.44	0.44	0.27	0.44	0.44
110	H-N(4)	H-C_{β}(5)	-	0.45	0.47	0.48	0.48	0.47	0.47	0.47	0.51	0.47	0.47
111	H-N(4)	Me-$C_{\delta 1}$(5)	-	0.57	*0.70*	0.67	*0.69*	*0.69*	*0.69*	*0.70*	0.61	*0.70*	*0.70*
112	H_{Si}-C_{α}(5)	H-N(6)	-	0.34	0.27	0.25	0.27	0.27	0.28	0.28	0.24	0.28	0.27
113	H-C_{β}(5)	H-N(6)	-	0.43	0.42	0.31	0.40	0.42	0.44	0.44	0.28	0.44	0.44
114	H-N(5)	H-C_{β}(6)	-	0.44	0.50	0.52	0.50	0.50	0.49	0.50	0.53	0.50	0.50
115	H-N(6)	H-C_{β}(7)	-	0.42	*0.54*	*0.54*	*0.54*	*0.54*	*0.53*	*0.54*	0.52	*0.54*	*0.55*
116	H-C_{β}(6)	H-N(7)	-	0.42	0.39	0.32	0.40	0.38	0.42	0.41	0.31	0.41	0.41
117	H-C_{γ}(1)	H-C_{β}(4)	-	0.35	0.39	*0.46*	0.39	0.40	0.40	0.39	*0.54*	0.40	0.39
118	H-N(2)	Me-C_{δ}(4)	-	0.60	0.48	0.54	0.48	0.47	0.48	0.47	0.60	0.48	0.47
119	Me-C_{γ}(2)	H-C_{β}(5)	-	0.44	0.37	0.43	0.37	0.37	0.37	0.37	0.49	0.37	0.37
120	Me-C_{γ}(2)	Me-$C_{\delta 2}$(5)	-	0.63	0.45	0.53	0.46	0.45	0.45	0.45	0.58	0.46	0.45
121	H-N(2)	Me-$C_{\delta 2}$(5)	-	0.58	0.48	0.55	0.49	0.48	0.49	0.48	0.61	0.48	0.47
122	H-N(2)	H-C_{γ}(5)	-	0.53	0.48	0.55	0.47	0.47	0.47	0.47	0.58	0.48	0.47
123	H-N(3)	Me-$C_{\delta 2}$(5)	-	0.60	0.65	*0.72*	0.65	0.64	0.64	0.64	0.66	0.64	0.64
124	H-N(3)	Me-C_{γ}(6)	-	0.62	0.47	0.53	0.47	0.47	0.48	0.47	0.61	0.47	0.46
125	H_{Re}-C_{γ}(3)	H-C_{β}(6)	-	0.29	0.28	0.32	0.28	0.28	0.28	0.27	0.38	0.27	0.27
126	H_{Si}-C_{γ}(3)	H-C_{β}(6)	-	0.32	0.32	0.36	0.31	0.32	0.32	0.31	*0.43*	0.32	0.31
127	H-N(3)	H_{Si}-C_{α}(6)	-	0.45	0.52	*0.59*	0.53	0.53	0.52	0.53	*0.63*	0.53	0.53
128	H_{Re}-C_{α}(4)	H-C_{β}(7)	-	0.24	0.32	*0.35*	0.32	0.31	0.30	0.32	*0.41*	0.31	0.32

129	Me-$C_γ$(4)	H-$C_β$(7)	-	0.45	0.37	0.43	0.38	0.37	0.37	0.37	0.50	0.37	0.37
130	Me-$C_γ$(4)	Me-$C_{ε1}$(7)	-	0.65	0.41	0.47	0.41	0.41	0.40	0.40	0.53	0.40	0.41
131	H-N(5)	H-$C_β$(7)	-	0.36	0.44	*0.48*	0.43	0.43	0.42	0.43	*0.55*	0.43	0.44
Nviol42					0	0	0	0	0	0	4	0	0
RMSD42					0.01	0.03	0.01	0.01	0.01	0.01	0.05	0.01	0.01
Nviol119					3	6	3	3	3	3	5	3	3
RMSD119					0.03	0.04	0.03	0.03	0.03	0.03	0.05	0.03	0.03

Table S7. List of 21 ^{3}J-coupling constants (Hz) derived from experiment and averaged from unrestrained and RDC-restrained MD simulations of the ß-heptapeptide. *MDsol*: MD simulation of the peptide solvated in methanol without any restraining of the molecule ($K^{RDC,msy}$ = 0 kJmol^{-1}Hz^{-2}). *HRSrMDsol*: RDC-restraining MD simulations with $K^{RDC,msy}$ = 0.05 kJmol^{-1}Hz^{-2} in which the parameters $K^{RDC,mfv}$, $\tau_{\theta}^{RDC,mfv}$ and N_{mfv} were varied. Other parameter values of the t^{msy} = 100 ns *HRS* simulations are γ^{mfv} = 2.4 ps^{-1}, ΔD^{fb} = 2.0 Hz, $\tau_{\theta}^{RDC,msy}$ = 1 ns. *Ndev*: number of deviations between simulation and experiment larger than 2 Hz. *RMSD*: root-mean-square difference between experiment (Ref.[45]) and simulation for the 21 ^{3}J-coupling constants (Hz). The residue sequence numbers of the atoms are within parentheses. Experimentally derived ^{3}J-couplings from Table 2 of Ref.[45] and from Tables 2 and 3 of Ref.[24]. MD values differing more than 2 Hz from the experimental value are in (red) italics.

^{3}J-coupling sequence number	^{3}J-coupling dihedral angle	Exp. value (Hz)		^{3}J-coupling (Hz)								
Simulation		Ref. [45]	Ref. [24]	*MDsol*	*HRSrMDsol*							
$K^{RDC,mfv}$	kJmol^{-1}Hz^{-2}				10	10	10	10	10	100	100	100
$\tau_{\theta}^{RDC,mfv}$	ns				10	10	10	100	100	100	100	100
N_{mfv}					10	100	1000	100	1000	10	100	1000
$K^{RDC,msy}$	kJmol^{-1}Hz^{-2}			0	0.05	0.05	0.05	0.05	0.05	0.05	0.05	0.05
1	$H_{ß}(1)$-$C_{ß}(1)$-$C_{\alpha}(1)$-$H_{\alpha Si}(1)$	2.8		2.6	3.4	2.7	2.8	3.0	2.7	3.8	2.7	2.6
2	$H_{ß}(2)$-$C_{ß}(2)$-$C_{\alpha}(2)$-$H_{\alpha Si}(2)$	4.5	4.4	3.7	5.1	3.7	3.8	3.8	3.8	6.4	3.8	3.8
3	$H_{ß}(3)$-$C_{ß}(3)$-$C_{\alpha}(3)$-$H_{\alpha Si}(3)$	4.5	4.2	3.9	4.9	3.9	4.0	4.0	4.0	5.4	4.0	4.0
4	$H_{ß}(5)$-$C_{ß}(5)$-$C_{\alpha}(5)$-$H_{\alpha Si}(5)$	3.9	3.7	3.4	4.1	3.4	3.4	3.6	3.4	4.3	3.4	3.4
5	$H_{ß}(6)$-$C_{ß}(6)$-$C_{\alpha}(6)$-$H_{\alpha Si}(6)$	3.8	4.1	3.3	5.4	3.8	3.5	3.4	3.2	5.9	3.2	3.2
6	$H_{ß}(7)$-$C_{ß}(7)$-$C_{\alpha}(7)$-$H_{\alpha Si}(7)$	4.5		4.1	4.9	4.2	4.1	4.2	3.9	4.9	4.1	4.4
7	$H_{ß}(1)$-$C_{ß}(1)$-$C_{\gamma}(1)$-$H_{\gamma}(1)$	4.7	5.1	5.0	4.8	4.7	4.3	4.4	4.6	4.7	4.4	4.6
8	$H_{ß}(5)$-$C_{ß}(5)$-$C_{\gamma}(5)$-$H_{\gamma}(5)$	7.0	7.0	6.4	6.1	6.0	6.2	5.5	6.2	5.8	6.2	6.5

9	H_{N}(2)-N(2)-C_{β}(2)-H_{β}(2)	9.2	9.1	9.2	8.8	9.2	9.2	9.1	9.2	8.7	9.2	9.2
10	H_{N}(3)-N(3)-C_{β}(3)-H_{β}(3)	9.6	9.2	9.1	9.1	9.2	9.1	9.1	9.1	9.0	9.1	9.1
11	H_{N}(4)-N(4)-C_{β}(4)-H_{β}(4)	9.3	9.0	9.3	9.1	9.3	9.3	9.3	9.3	8.9	9.3	9.3
12	H_{N}(5)-N(5)-C_{β}(5)-H_{β}(5)	9.6	9.4	9.3	9.2	9.3	9.3	9.3	9.3	9.0	9.3	9.4
13	H_{N}(6)-N(6)-C_{β}(6)-H_{β}(6)	8.7	8.6	9.2	8.9	9.2	9.2	9.2	9.2	8.6	9.2	9.2
14	H_{N}(7)-N(7)-C_{β}(7)-H_{β}(7)	9.5	9.3	9.2	9.0	9.2	9.2	9.1	9.2	9.0	9.2	9.2
15	H_{β}(1)-C_{β}(1)-C_{α}(1)-$H_{\alpha Re}$(1)	11.5	11.7	11.9	10.8	11.9	11.7	11.4	11.9	10.1	11.8	11.9
16	H_{β}(2)-C_{β}(2)-C_{α}(2)-$H_{\alpha Re}$(2)	12.0	12.1	12.6	10.0	12.6	12.4	12.4	12.6	*8.4*	12.3	12.6
17	H_{β}(3)-C_{β}(3)-C_{α}(3)-$H_{\alpha Re}$(3)	12.3		12.5	10.3	12.5	12.4	12.5	12.6	*9.1*	12.6	12.6
18	H_{β}(4)-C_{β}(4)-C_{α}(4)-$H_{\alpha Re}$(4)	10.8	10.9	12.4	*8.5*	12.4	12.1	12.4	12.4	*6.8*	12.4	12.4
19	H_{β}(5)-C_{β}(5)-C_{α}(5)-$H_{\alpha Re}$(5)	12.3	12.2	12.5	10.7	12.4	12.4	12.6	12.6	10.3	12.6	12.5
20	H_{β}(6)-C_{β}(6)-C_{α}(6)-$H_{\alpha Re}$(6)	11.6	11.4	11.4	*9.2*	11.0	11.3	11.6	11.6	*7.6*	11.6	11.5
21	H_{β}(7)-C_{β}(7)-C_{α}(7)-$H_{\alpha Re}$(7)	10.0		9.4	8.3	10.0	9.5	9.1	9.4	8.7	9.6	9.4
Ndev				0	2	0	0	0	0	4	0	0
RMSD				0.5	1.2	0.6	0.5	0.6	0.6	1.9	0.5	0.5

Table S8. Average occurrence of the three hydrogen bonds NH(2)-O(4), NH(3)-O(5) and NH(4)-O(6) (characterising the left-handed M-3_{14}-helical fold) in the MD simulations of the β-heptapeptide solvated in methanol. $K^{RDC,msy}$ = 0: MD simulation without any restraining of the molecule (*MDsol*). $K^{RDC,msy}$ > 0: MD simulation with RDC-restraining of the molecule (*HRSrMDsol*). Parameter values of the t^{msy} = 100 ns *HRS* simulations are γ^{mfv} = 2.4 ps^{-1} and ΔD^{fb} = 2.0 Hz, while the values of the parameters $K^{RDC,mfv}$ (kJmol^{-1}Hz^{-2}), $\tau_{\theta}^{RDC,mfv}$ (ns), N_{mfv} , $K^{RDC,msy}$ (kJmol^{-1}Hz^{-2}) and $\tau_{\theta}^{RDC,msy}$ (ns) were varied. Target RDC-values D_k^0 from Tables 1 and 2 of Ref.[24]. The hydrogen-bond definition is mentioned in Section 4.7. Hydrogen-bonding percentages smaller than 80% are in (red) italics. "*sf*": Result not available due to SHAKE failure: too large (restraining) forces.

$K^{RDC,mfv}$ (kJmol^{-1}Hz^{-2})	$\tau_{\theta}^{RDC,mfv}$ (ns)	N_{mfv}	$\tau_{\theta}^{RDC,msy}$ = 0.1 ns			$\tau_{\theta}^{RDC,msy}$ = 1 ns	
			$K^{RDC,msy}$ = 0	$K^{RDC,msy}$ = 0.05	$K^{RDC,msy}$ = 0.5	$K^{RDC,msy}$ = 0.05	$K^{RDC,msy}$ = 0.5
1	10	10	86	*sf*	*sf*	*1*	*sf*
		100	86	*1*	*sf*	80	*0*
		1000	86	*44*	*0*	95	*5*
	100	10	86			*1*	*sf*
		100	86			*6*	*sf*
		1000	86			94	*1*
10	10	10	86	*0*	*sf*	*35*	*sf*
		100	86	*70*	*sf*	85	*6*
		1000	86	95	*3*	88	*12*
	100	10	86	*sf*	*sf*	*1*	*sf*
		100	86	*1*	*sf*	91	*1*
		1000	86	*71*	*1*	95	*3*
100	10	10	86	*3*	*sf*	85	*1*
		100	86	90	*5*	89	95
		1000	86	95		95	93
	100	10	86			*14*	*sf*
		100	86			92	*1*
		1000	86			94	97
1000	10	10	86	*45*	*sf*		
		100	86	96	*1*		

Table S9. Intra-molecular M-3_{14}-helical hydrogen bonds (%) from unrestrained and RDC-restrained MD simulations of the ß-heptapeptide. *MDsol*: MD simulation of the peptide solvated in methanol without any restraining of the molecule ($K^{RDC,msy}$ = 0 kJmol^{-1}Hz^{-2}, using the *HRS*-method). *HRSrMDsol*: RDC-restraining MD simulations with $K^{RDC,msy}$ = 0.05 kJmol^{-1}Hz^{-2} in which the parameters $K^{RDC,mfv}$, $\tau_\theta^{RDC,mfv}$ and N_{mfv} were varied. Other parameter values of the t^{msy} = 100 ns *HRS* simulations are γ^{mfv} = 2.4 ps^{-1}, ΔD^{fb} = 2.0 Hz, $\tau_\theta^{RDC,msy}$ = 1 ns. The residue sequence numbers of the atoms are within parentheses.

Hydrogen-bond sequence number	Donor and acceptor atoms		% hydrogen-bonding								
Simulation			*MDsol*	*HRSrMDsol*							
$K^{RDC,mfv}$	kJmol^{-1}Hz^{-2}			10	10	10	10	10	100	100	100
$\tau_\theta^{RDC,mfv}$	ns			10	10	10	100	100	100	100	100
N_{mfv}				10	100	1000	100	1000	10	100	1000
$K^{RDC,msy}$	kJmol^{-1}Hz^{-2}		0	0.05	0.05	0.05	0.05	0.05	0.05	0.05	0.05
1	NH1(1)	O(3)	8	4	8	7	7	8	2	7	8
2	NH2(1)	O(3)	8	4	8	7	7	7	2	7	8
3	NH(2)	O(4)	87	34	87	90	89	96	14	89	97
4	NH(3)	O(5)	88	37	88	91	94	98	14	98	98
5	NH(4)	O(6)	81	35	79	83	91	90	13	90	86

Table S10. List of 39 RDC-values (Hz) derived experimentally, D_k^0, and the averages <D_k> from MD simulations of the ß-heptapeptide solvated in methanol with RDC-restraining of the magnetic-field vector and the molecule (*HRSrMDsol*, $K^{RDC,msy}$ = 0.05 kJmol^{-1}Hz^{-2}, $\tau_\theta^{RDC,msy}$ = 1 ns) using four different (sub)sets of RDCs. *RDCa*: all 39 RDCs, *RDCbb*: only the 29 backbone RDCs, No 1 – 29. *RDCHN*: only the 6 H - N backbone RDCs, No 1 – 6. *RDCHBCB*: only the 7 backbone HB – CB RDCs, No 7 - 13. Parameter values of the t^{msy} = 100 ns *HRS* simulations are γ^{mfv} = 2.4 ps^{-1}, $K^{RDC,mfv}$ = 100 kJmol^{-1}Hz^{-2}, $\tau_\theta^{RDC,mfv}$ = 100 ns N_{mfv} = 100, and ΔD^{fb} = 2.0 Hz. The residue sequence numbers of the atoms are within parentheses. RDC-values D_k^0 from Tables 1 and 2 of Ref.[24]. The values for the RDCs that are *not* part of the subset of RDC restraints applied, are in bold. *RMSD*: RMSD-values calculated over all, *mfv*-restrained and unrestrained, RDCs. *rRMSD*: RMSD-values calculated over the particular subset of *mfv*-restrained RDCs. *urRMSD*: RMSD-values calculated over the unrestrained RDCs. Deviations of averaged RDC-values $< D_{k_1k_2} >_{t^{msy}}$ from their target values $D_{k_1k_2}^0$ larger than 2 Hz are in (red) italics.

RDC sequence number	RDC atoms		D_k^0 (Hz)	$< D_{k_1k_2} >_{t^{msy}}$ (Hz)			
				RDCa	*RDCbb*	*RDCHN*	*RDCHBCB*
1	^{1}H(2)	^{15}N(2)	3.4	3.7	3.5	3.4	**3.6**
2	^{1}H(3)	^{15}N(3)	5.6	5.3	5.4	5.5	**6.8**
3	^{1}H(4)	^{15}N(4)	4.4	4.8	4.5	4.6	**5.7**
4	^{1}H(5)	^{15}N(5)	4.5	4.5	4.3	4.2	**3.6**
5	^{1}H(6)	^{15}N(6)	5.2	5.0	5.1	4.9	**4.7**
6	^{1}H(7)	^{15}N(7)	4.7	4.1	4.1	4.3	**3.7**
7	^{1}H$_ß$(1)	^{13}C$_ß$(1)	-6.9	-6.5	-6.7	**-7.4**	-6.9
8	^{1}H$_ß$(2)	^{13}C$_ß$(2)	-12.3	-12.2	-11.9	***-15.0***	-12.0
9	^{1}H$_ß$(3)	^{13}C$_ß$(3)	-17.1	-15.4	-16.5	**-15.8**	-16.6
10	^{1}H$_ß$(4)	^{13}C$_ß$(4)	-12.0	-11.9	-12.3	***-15.5***	-12.2
11	^{1}H$_ß$(5)	^{13}C$_ß$(5)	-11.3	-11.2	-11.5	**-13.3**	-11.3
12	^{1}H$_ß$(6)	^{13}C$_ß$(6)	-12.6	-12.6	-12.6	***-16.2***	-13.2
13	^{1}H$_ß$(7)	^{13}C$_ß$(7)	-12.8	-12.0	-12.3	**-12.0**	-12.4
14	^{1}H$_{\alpha Re}$(1)	^{13}C$_\alpha$(1)	-6.2	-6.4	-6.1	***-11.8***	**-6.0**
15	^{1}H$_{\alpha Re}$(2)	^{13}C$_\alpha$(2)	-11.0	-11.0	-10.8	***-14.7***	**-12.8**

16	$^{1}H_{\alpha Re}(4)$	$^{13}C_{\alpha}(4)$	-12.5	-12.3	-12.4	***-16.2***	**-13.4**
17	$^{1}H_{\alpha Re}(6)$	$^{13}C_{\alpha}(6)$	-8.6	-9.6	-9.7	***-11.1***	**-8.9**
18	$^{1}H_{\alpha Re}(7)$	$^{13}C_{\alpha}(7)$	-8.7	-8.3	-8.3	***-3.7***	**-9.8**
19	$^{1}H_{\alpha Si}(1)$	$^{13}C_{\alpha}(1)$	1.5	1.3	1.5	**0.8**	**2.2**
20	$^{1}H_{\alpha Si}(2)$	$^{13}C_{\alpha}(2)$	6.9	7.0	7.0	***14.7***	***11.3***
21	$^{1}H_{\alpha Si}(5)$	$^{13}C_{\alpha}(5)$	10.1	9.5	9.6	**9.3**	**10.4**
22	$^{1}H_{\alpha Si}(6)$	$^{13}C_{\alpha}(6)$	7.1	6.1	5.9	***4.9***	***1.6***
23	$^{1}H_{\alpha Si}(7)$	$^{13}C_{\alpha}(7)$	2.2	2.4	2.4	**2.4**	***6.9***
24	$^{1}H_{\alpha Re}(1)$	$^{1}H_{\alpha Si}(1)$	-1.5	-1.1	-1.4	***-6.4***	**-0.3**
25	$^{1}H_{\alpha Re}(2)$	$^{1}H_{\alpha Si}(2)$	1.1	0.6	0.8	**2.1**	**1.5**
26	$^{1}H_{\alpha Re}(3)$	$^{1}H_{\alpha Si}(3)$	-0.9	-1.3	-1.1	***-3.4***	***-5.3***
27	$^{1}H_{\alpha Re}(5)$	$^{1}H_{\alpha Si}(5)$	2.4	2.9	2.4	**1.1**	**3.5**
28	$^{1}H_{\alpha Re}(6)$	$^{1}H_{\alpha Si}(6)$	-0.2	1.0	0.9	***2.1***	**-2.1**
29	$^{1}H_{\alpha Re}(7)$	$^{1}H_{\alpha Si}(7)$	-2.0	-2.3	-2.3	***1.7***	***0.3***
30	$^{1}H_{\gamma}(1)$	$^{13}C_{\gamma}(1)$	-1.2	-1.0	***-4.5***	***-6.0***	**-2.3**
31	$^{1}H_{\gamma Re}(3)$	$^{13}C_{\gamma}(3)$	-2.9	-3.2	***0.2***	***3.4***	***2.4***
32	$^{1}H_{\gamma Si}(3)$	$^{13}C_{\gamma}(3)$	4.1	2.4	***-8.5***	***-11.2***	***-10.9***
33	$^{1}H_{\gamma Re}(3)$	$^{1}H_{\gamma Si}(3)$	0.8	1.4	***-5.8***	***-3.0***	***-4.7***
34	$^{1}H_{\delta}(3)$	$^{13}C_{\delta}(3)$	6.0	5.6	**5.0**	**7.6**	**5.8**
35	$^{1}H_{\gamma}(5)$	$^{13}C_{\gamma}(5)$	-4.1	-3.9	**-4.2**	***1.5***	***-1.4***
36	$^{1}H_{\gamma Re}(7)$	$^{13}C_{\gamma}(7)$	-3.6	-3.7	**-3.0**	**-4.6**	***1.4***
37	$^{1}H_{\gamma Si}(7)$	$^{13}C_{\gamma}(7)$	2.3	1.9	***-1.2***	**0.5**	***-4.9***
38	$^{1}H_{\gamma Re}(7)$	$^{1}H_{\gamma Si}(7)$	-1.4	-1.0	**-0.9**	***-6.2***	***-6.2***
39	$^{1}H_{\delta}(7)$	$^{13}C_{\delta}(7)$	6.0	5.7	***1.2***	***3.8***	***11.9***
RMSD				0.6	*2.6*	*4.0*	*3.7*
rRMSD				0.6	0.5	0.2	0.4
urRMSD				-	***5.1***	***4.3***	***4.1***

Table S11. Averages and root-mean-square fluctuations (*RMSF*) of 19 backbone and 6 side-chain torsional angles (degree) from MD simulations of the ß-heptapeptide solvated in methanol without any restraining of the molecule (*MDsol*, $K^{RDC,msy}$ = 0 kJmol^{-1}Hz^{-2}) using four different (sub)sets of RDCs. *RDCa*: all 39 RDCs, *RDCbb*: only the 29 backbone RDCs, No 1 – 29. *RDCHN*: only the 6 H - N backbone RDCs, No 1 – 6. *RDCHBCB*: only the 7 backbone HB – CB RDCs, No 7 - 13. Parameter values of the t^{msy} = 100 ns *HRS* simulations are γ^{mfv} = 2.4 ps^{-1}, $K^{RDC,mfv}$ = 100 kJmol^{-1}Hz^{-2}, $\tau_{\theta}^{RDC,mfv}$ = 100 ns N_{mfv} = 100, and ΔD^{fb} = 2.0 Hz. The residue sequence numbers of the atoms are within parentheses.

	Torsional angle	Average angle and fluctuations (degree)							
Simulation		*RDCa*		*RDCbb*		*RDCHN*		*RDCHBCB*	
		<angle>	*RMSF*	<angle>	*RMSF*	<angle>	*RMSF*	<angle>	*RMSF*
backbone									
1	N(1)-$C_ß$(1)-$C_α$(1)-C(1)	74	17	74	17	74	18	74	17
2	$C_ß$(1)-$C_α$(1)-C(1)-N(2)	-43	147	-48	147	-59	143	-58	144
3	C(1)-N(2)-$C_ß$(2)-$C_α$(2)	-125	19	-126	16	-126	16	-125	17
4	N(2)-$C_ß$(2)-$C_α$(2)-C(2)	55	23	58	10	58	10	58	12
5	$C_ß$(2)-$C_α$(2)-C(2)-N(3)	-117	67	-131	33	-133	24	-130	37
6	C(2)-N(3)-$C_ß$(3)-$C_α$(3)	-129	15	-129	14	-129	14	-129	14
7	N(3)-$C_ß$(3)-$C_α$(3)-C(3)	56	9	56	9	56	9	56	9
8	$C_ß$(3)-$C_α$(3)-C(3)-N(4)	-142	10	-142	10	-142	11	-142	10
9	C(3)-N(4)-$C_ß$(4)-$C_α$(4)	-125	10	-125	10	-124	10	-125	10
10	N(4)-$C_ß$(4)-$C_α$(4)-C(4)	51	9	51	9	51	9	51	9
11	$C_ß$(4)-$C_α$(4)-C(4)-N(5)	-140	10	-140	10	-140	10	-140	9
12	C(4)-N(5)-$C_ß$(5)-$C_α$(5)	-123	9	-123	9	-124	9	-123	9
13	N(5)-$C_ß$(5)-$C_α$(5)-C(5)	61	10	61	10	60	9	60	9
14	$C_ß$(5)-$C_α$(5)-C(5)-N(6)	-129	69	-131	64	-137	51	-141	35
15	C(5)-N(6)-$C_ß$(6)-$C_α$(6)	-124	14	-124	15	-125	13	-125	12
16	N(6)-$C_ß$(6)-$C_α$(6)-C(6)	69	30	70	25	72	22	68	17
17	$C_ß$(6)-$C_α$(6)-C(6)-N(7)	-6	145	20	144	17	144	-19	144

18	C(6)-N(7)-$C_ß$(7)-$C_α$(7)	-117	18	-117	17	-118	17	-118	16
19	N(7)-$C_ß$(7)-$C_α$(7)-C(7)	69	59	70	58	71	53	70	51
side-chains									
20	$C_α$(1)-$C_ß$(1)-$C_γ$(1)-$C_{δ1}$(1)	31	101	31	100	48	102	38	103
21	$C_α$(3)-$C_ß$(3)-$C_γ$(3)-$C_δ$(3)	79	64	79	66	79	66	79	69
22	$C_ß$(3)-$C_γ$(3)-$C_δ$(3)-$C_{ε1}$(3)	78	64	78	66	80	65	80	68
23	$C_α$(5)-$C_ß$(5)-$C_γ$(5)-$C_{δ1}$(5)	55	93	56	94	43	93	48	91
24	$C_α$(7)-$C_ß$(7)-$C_γ$(7)-$C_δ$(7)	86	86	86	84	85	80	86	83
25	$C_ß$(7)-$C_γ$(7)-$C_δ$(7)-$C_{ε1}$(7)	85	84	86	81	85	78	87	79

Table S12. List of 39 RDC-values (Hz) derived from experiment, D_k^0, and the averages $<D_k>$ from unrestrained and RDC-restrained (ΔD^{fb} = 2.0 Hz) $t^{msy} = t^{AT}$ = 100 ns MD or SD simulations of the ß-heptapeptide. *MDsol*: MD simulation of the peptide solvated in methanol without any restraining of the molecule ($K^{RDC,msy}$ = 0 kJmol^{-1}Hz^{-2} using the *HRS* method). *HRSrMDsol*: RDC-restraining MD simulations with $K^{RDC,msy}$ = 0.05 kJmol^{-1}Hz^{-2} and $\tau_\theta^{RDC,msy}$ = 1 ns. *SDvac*: SD simulation of the peptide in vacuo without any restraining of the molecule ($K^{RDC,msy}$ = 0 kJmol^{-1}Hz^{-2} using the *HRS* method). *HRSrSDvac*: RDC-restraining SD simulations with $K^{RDC,msy}$ = 0.05 kJmol^{-1}Hz^{-2} and $\tau_\theta^{RDC,msy}$ = 1 ns. The *mfv* parameters of the *HRS* method have the values γ^{mfv} = 2.4 ps^{-1}, $K^{RDC,mfv}$ = 100 kJmol^{-1}Hz^{-2}, $\tau_\theta^{RDC,mfv}$ = 100 ns and N_{mfv} = 100. *ATrSDvac*: RDC-restraining SD simulations of the peptide in vacuo using the alignment-tensor (*AT*) formalism with $\tau_D^{RDC} = \tau_{AT}^{RDC}$ = 0 and $K^{RDC,AT}$ = 0.1 or 10 kJmol^{-1}Hz^{-2}. *RMSD*: root-mean-square difference between $< D_{k_1k_2} >_{t^{msy}}$ and $D_{k_1k_2}^0$ for the 39 bond-vector RDCs. The residue sequence numbers of the atoms are within parentheses. RDC-values D_k^0 from Tables 1 and 2 of Ref.[24]. Deviations of averaged RDC-values $< D_{k_1k_2} >_{t^{msy}}$ from their target values $D_{k_1k_2}^0$ larger than 2 Hz are in (red) italics.

RDC sequence number	RDC atoms		D_k^0 (Hz)	$< D_{k_1k_2} >_{t^{msy}}$ (Hz)					
Simulation				*MDsol*	*HRSrMDsol*	*SDvac*	*HRSrSDvac*	*ATrSDvac*	
$K^{RDC,AT}$	kJmol^{-1}Hz^{-2}							0.1	10
1	^{1}H(2)	^{15}N(2)	3.4	3.5	3.7	2.7	3.0	*0.7*	*0.0*
2	^{1}H(3)	^{15}N(3)	5.6	5.3	5.3	5.5	5.6	*2.1*	*-0.2*
3	^{1}H(4)	^{15}N(4)	4.4	4.5	4.8	4.0	4.6	*0.8*	*-0.2*
4	^{1}H(5)	^{15}N(5)	4.5	4.3	4.5	4.2	4.1	*1.1*	*-0.2*
5	^{1}H(6)	^{15}N(6)	5.2	4.9	5.0	5.0	4.8	*0.5*	*-0.2*
6	^{1}H(7)	^{15}N(7)	4.7	4.6	4.1	4.9	4.3	*1.4*	*-0.0*
7	^{1}Hß(1)	^{13}Cß(1)	-6.9	-6.5	-6.5	-6.6	-6.7	*-2.0*	*-0.5*
8	^{1}Hß(2)	^{13}Cß(2)	-12.3	-12.3	-12.2	-12.5	-11.8	*-4.0*	*-0.5*
9	^{1}Hß(3)	^{13}Cß(3)	-17.1	-15.3	-15.4	-15.7	-15.7	*-4.6*	*-0.6*
10	^{1}Hß(4)	^{13}Cß(4)	-12.0	-12.1	-11.9	-11.8	-11.6	*-1.8*	*-0.2*
11	^{1}Hß(5)	^{13}Cß(5)	-11.3	-11.1	-11.2	-11.4	-11.5	*-3.3*	*-0.1*

12	$^{1}H_{ß}(6)$	$^{13}C_{ß}(6)$	-12.6	-12.1	-12.6	-12.1	-12.2	*-2.4*	*-0.6*
13	$^{1}H_{ß}(7)$	$^{13}C_{ß}(7)$	-12.8	-11.7	-12.0	-11.8	-12.3	*-3.8*	*-0.4*
14	$^{1}H_{\alpha Re}(1)$	$^{13}C_{\alpha}(1)$	-6.2	-6.4	-6.4	-6.5	-6.1	*-2.8*	*-0.5*
15	$^{1}H_{\alpha Re}(2)$	$^{13}C_{\alpha}(2)$	-11.0	-10.6	-11.0	-10.8	-11.0	*-4.3*	*-0.8*
16	$^{1}H_{\alpha Re}(4)$	$^{13}C_{\alpha}(4)$	-12.5	-12.5	-12.3	-12.2	-11.9	*-3.8*	*-0.2*
17	$^{1}H_{\alpha Re}(6)$	$^{13}C_{\alpha}(6)$	-8.6	-9.7	-9.6	-9.1	-8.8	*-4.1*	*-0.6*
18	$^{1}H_{\alpha Re}(7)$	$^{13}C_{\alpha}(7)$	-8.7	-8.5	-8.3	-8.1	-8.1	*-2.4*	*-0.6*
19	$^{1}H_{\alpha Si}(1)$	$^{13}C_{\alpha}(1)$	1.5	1.4	1.3	1.5	1.7	-0.2	0.1
20	$^{1}H_{\alpha Si}(2)$	$^{13}C_{\alpha}(2)$	6.9	7.1	7.0	6.8	6.8	*2.7*	*0.4*
21	$^{1}H_{\alpha Si}(5)$	$^{13}C_{\alpha}(5)$	10.1	9.7	9.5	9.2	9.5	*-1.0*	*0.1*
22	$^{1}H_{\alpha Si}(6)$	$^{13}C_{\alpha}(6)$	7.1	6.1	6.1	7.3	7.0	*1.1*	*0.6*
23	$^{1}H_{\alpha Si}(7)$	$^{13}C_{\alpha}(7)$	2.2	2.4	2.6	2.6	2.4	3.7	0.2
24	$^{1}H_{\alpha Re}(1)$	$^{1}H_{\alpha Si}(1)$	-1.5	-1.2	-1.1	-1.5	-1.8	-2.4	-0.2
25	$^{1}H_{\alpha Re}(2)$	$^{1}H_{\alpha Si}(2)$	1.1	0.6	0.6	1.1	1.0	*-1.8*	-0.3
26	$^{1}H_{\alpha Re}(3)$	$^{1}H_{\alpha Si}(3)$	-0.9	-1.4	-1.3	-1.2	-1.2	-2.4	0.1
27	$^{1}H_{\alpha Re}(5)$	$^{1}H_{\alpha Si}(5)$	2.4	2.5	2.9	2.9	2.5	*-1.1*	*-0.1*
28	$^{1}H_{\alpha Re}(6)$	$^{1}H_{\alpha Si}(6)$	-0.2	0.7	1.0	-0.4	-0.5	-1.8	-0.0
29	$^{1}H_{\alpha Re}(7)$	$^{1}H_{\alpha Si}(7)$	-2.0	-2.1	-2.3	-2.0	-2.0	*0.4*	-0.3
30	$^{1}H_{\gamma}(1)$	$^{13}C_{\gamma}(1)$	-1.2	-1.4	-1.0	-1.1	-1.2	-0.9	-0.0
31	$^{1}H_{\gamma Re}(3)$	$^{13}C_{\gamma}(3)$	-2.9	-3.3	-3.2	-3.5	-3.4	-1.4	*-0.1*
32	$^{1}H_{\gamma Si}(3)$	$^{13}C_{\gamma}(3)$	4.1	2.8	2.4	3.1	3.1	*0.2*	*0.1*
33	$^{1}H_{\gamma Re}(3)$	$^{1}H_{\gamma Si}(3)$	0.8	1.1	1.4	1.2	1.2	-0.6	0.1
34	$^{1}H_{\delta}(3)$	$^{13}C_{\delta}(3)$	6.0	5.9	5.6	5.9	5.6	*1.9*	*0.4*
35	$^{1}H_{\gamma}(5)$	$^{13}C_{\gamma}(5)$	-4.1	-4.2	-3.9	-3.9	-3.7	*-0.6*	*-0.1*
36	$^{1}H_{\gamma Re}(7)$	$^{13}C_{\gamma}(7)$	-3.6	-4.0	-3.7	-4.1	-3.8	*-0.3*	*-0.1*
37	$^{1}H_{\gamma Si}(7)$	$^{13}C_{\gamma}(7)$	2.3	1.6	1.9	1.7	1.7	*-0.7*	*0.1*
38	$^{1}H_{\gamma Re}(7)$	$^{1}H_{\gamma Si}(7)$	-1.4	-1.1	-1.0	-0.7	-0.8	-0.6	-0.0
39	$^{1}H_{\delta}(7)$	$^{13}C_{\delta}(7)$	6.0	5.8	5.7	5.5	5.7	*2.3*	*0.4*
RMSD				0.6	0.6	0.5	0.4	*5.5*	*7.0*

Table S13. Intra-molecular M-3_{14}-helical hydrogen bonds (%) from unrestrained and RDC-restrained (ΔD^{fb} = 2.0 Hz) $t^{msy} = t^{AT}$ = 100 ns MD simulations of the ß-heptapeptide. *MDsol*: MD simulation of the peptide solvated in methanol without any restraining of the molecule ($K^{RDC,msy}$ = 0 kJmol^{-1}Hz^{-2} using the *HRS* method). *HRSrMDsol*: RDC-restraining MD simulations with $K^{RDC,msy}$ = 0.05 kJmol^{-1}Hz^{-2} and $\tau_{\theta}^{RDC,msy}$ = 1 ns. *SDvac*: SD simulation of the peptide in vacuo without any restraining of the molecule ($K^{RDC,msy}$ = 0 kJmol^{-1}Hz^{-2} using the *HRS* method). *HRSrSDvac*: RDC-restraining SD simulations with $K^{RDC,msy}$ = 0.05 kJmol^{-1}Hz^{-2} and $\tau_{\theta}^{RDC,msy}$ = 1 ns. The *mfv* parameters of the *HRS* method have the values γ^{mfv} = 2.4 ps^{-1}, $K^{RDC,mfv}$ = 100 kJmol^{-1}Hz^{-2}, $\tau_{\theta}^{RDC,mfv}$ = 100 ns and N_{mfv} = 100. *ATrSDvac*: RDC-restraining SD simulations of the peptide in vacuo using the alignment-tensor (*AT*) formalism with τ_{D}^{RDC} = τ_{AT}^{RDC} = 0 and $K^{RDC,AT}$ = 0.1 or 10 kJmol^{-1}Hz^{-2}. The residue sequence numbers of the atoms are within parentheses.

Hydrogen-bond sequence number	Donor and acceptor atoms		% hydrogen-bonding					
Simulation			*MDsol*	*HRSrMDsol*	*SDvac*	*HRSrSDvac*	*ATrSDvac*	
$K^{RDC,AT}$	kJmol^{-1}Hz^{-2}						0.1	10
1	NH1(1)	O(3)	8	7	3	9	1	0
2	NH2(1)	O(3)	8	7	4	8	1	0
3	NH(2)	O(4)	87	89	92	89	3	2
4	NH(3)	O(5)	88	98	75	89	4	3
5	NH(4)	O(6)	81	90	23	56	4	1

Table S14. Lists of 42[45] and 119[24] NOE atom-atom upper distance (nm) bounds derived from experiment and the corresponding r^{-6} averaged distances (in nm) from unrestrained and RDC-restrained (ΔD^{fb} = 2.0 Hz) $t^{msy} = t^{AT}$ = 100 ns MD simulations of the ß-heptapeptide. *MDsol*: MD simulation of the peptide solvated in methanol without any restraining of the molecule ($K^{RDC,msy}$ = 0 kJmol^{-1}Hz^{-2} using the *HRS* method). *HRSrMDsol*: RDC-restraining MD simulations with $K^{RDC,msy}$ = 0.05 kJmol^{-1}Hz^{-2} and $\tau_{\theta}^{RDC,msy}$ = 1 ns. *SDvac*: SD simulation of the peptide in vacuo without any restraining of the molecule ($K^{RDC,msy}$ = 0 kJmol^{-1}Hz^{-2} using the *HRS* method). *HRSrSDvac*: RDC-restraining SD simulations with $K^{RDC,msy}$ = 0.05 kJmol^{-1}Hz^{-2} and $\tau_{\theta}^{RDC,msy}$ = 1 ns. The *mfv* parameters of the *HRS* method have the values γ^{mfv} = 2.4 ps^{-1}, $K^{RDC,mfv}$ = 100 kJmol^{-1}Hz^{-2}, $\tau_{\theta}^{RDC,mfv}$ = 100 ns and N_{mfv} = 100. *ATrSDvac*: RDC-restraining SD simulations of the peptide in vacuo using the alignment-tensor (*AT*) formalism with $\tau_{D}^{RDC} = \tau_{AT}^{RDC}$ = 0 and $K^{RDC,AT}$ = 0.1 or 10 kJmol^{-1}Hz^{-2}. *NOE distance upper bounds*: 42 from Table 1 of Ref.[45] and 119 (excluding five NOE bounds involving an "HT" atom) from Table S2 of Supplementary Information of Ref.[24]. *RMSD42*: root-mean-square deviations of the r^{-6} averaged distances beyond the 42 NOE upper bounds of Ref.[45]. *Nviol42*: number of distance-bound violations larger than 0.1 nm. *RMSD119*: root-mean-square deviations of the r^{-6} averaged distances beyond the 119 NOE upper bounds of Ref.[24]. *Nviol119*: number of distance-bound violations larger than 0.1 nm. The residue sequence numbers of the atoms are within parentheses. Values 0.1 nm larger than the largest experimentally derived NOE upper bound value are in (red) italics. "Me": three H-atoms of a methyl group. The three hydrogens of the methyl group attached to the C_{α}(4)-atom, denoted as Me-C_{α}(4) in Ref.[45], and as Hδ*(4) in Ref.[24], are denoted as Me-C_{δ}(4).

NOE sequence number	NOE H-atom pair		NOE distance upper bound (nm)		r^{-6} averaged distance (nm)					
					Simulation					
			Ref. [45]	Ref. [24]	*MDsol*	*HRSrMDsol*	*SDvac*	*HRSrSDvac*	*ATrSDvac*: $K^{RDC,AT}$ (kJmol^{-1}Hz^{-2})	
									0.1	10
1	H-N(1)	H-C$_{\beta}$(1)	0.28	-	0.26	0.26	0.26	0.26	0.26	0.26
2	H-C$_{\beta}$(1)	H$_{Re}$-C$_{\alpha}$(1)	0.29	0.28	0.28	0.28	0.28	0.28	0.28	0.28
3	H-C$_{\beta}$(1)	H$_{Si}$-C$_{\alpha}$(1)	0.30	0.24	0.24	0.24	0.24	0.24	0.24	0.24
4	H-N(2)	H$_{Re}$-C$_{\alpha}$(1)	0.24	0.26	0.24	0.24	0.23	0.23	0.24	0.25
5	H-N(2)	H$_{Si}$-C$_{\alpha}$(1)	0.29	0.33	0.25	0.25	0.25	0.25	0.24	0.24
6	H-N(2)	H-C$_{\beta}$(2)	0.33	-	0.28	0.28	0.28	0.28	0.24	0.27
7	H-N(2)	H-C$_{\beta}$(4)	0.35	0.33	0.36	0.36	0.35	0.36	0.40	*0.59*
8	H-N(2)	H-C$_{\beta}$(5)	0.33	0.31	0.31	0.30	0.31	0.31	*0.54*	*0.52*
9	H-C$_{\beta}$(2)	Me-C$_{\gamma}$(2)	0.32	0.38	0.24	0.24	0.24	0.24	0.24	0.24
10	H-C$_{\beta}$(2)	H$_{Si}$-C$_{\alpha}$(2)	0.23	0.23	0.24	0.24	0.24	0.24	0.23	0.24
11	H-N(3)	H$_{Re}$-C$_{\alpha}$(2)	0.22	0.24	0.21	0.22	0.23	0.22	0.24	0.25
12	H-N(3)	H$_{Si}$-C$_{\alpha}$(2)	0.31	-	0.30	0.28	0.26	0.27	0.27	0.26
13	H-N(3)	H-C$_{\beta}$(3)	0.31	-	0.29	0.29	0.28	0.28	0.28	0.24
14	H-N(3)	H$_{Re}$-C$_{\alpha}$(3)	0.26	0.29	0.24	0.24	0.25	0.25	0.27	0.30
15	H-N(3)	H-N(4)	0.38	-	0.38	0.37	0.33	0.34	0.45	0.33
16	H-N(3)	H-C$_{\beta}$(5)	0.34	0.34	0.36	0.35	0.38	0.36	*0.59*	*0.47*
17	H-N(3)	H-C$_{\beta}$(6)	0.32	0.31	0.30	0.30	0.28	0.32	*0.50*	*0.44*
18	H-C$_{\beta}$(3)	H-C$_{\delta}$(3)	0.30	-	0.27	0.27	0.27	0.27	0.27	0.26
19	H-N(4)	H$_{Re}$-C$_{\alpha}$(3)	0.23	0.24	0.22	0.21	0.26	0.23	0.28	0.25
20	H-N(4)	H$_{Si}$-C$_{\alpha}$(3)	0.28	0.44	0.28	0.29	0.28	0.26	0.24	0.26
21	H-N(4)	H-C$_{\beta}$(4)	0.29	-	0.29	0.29	0.22	0.24	0.28	0.27
22	H-N(4)	Me-C$_{\gamma}$(4)	0.40	0.48	0.29	0.29	0.32	0.31	0.30	0.30
23	H-N(4)	H-C$_{\beta}$(6)	0.32	0.33	0.36	0.36	*0.43*	0.38	0.35	*0.46*
24	H-N(4)	H-C$_{\beta}$(7)	0.37	0.31	0.30	0.29	0.41	0.35	*0.51*	*0.64*
25	H-C$_{\beta}$(4)	H$_{Re}$-C$_{\alpha}$(1)	0.26	0.23	0.25	0.25	0.33	0.30	*0.41*	*0.55*

26	H-N(5)	H-N(4)	0.37	-	0.38	0.38	0.41	0.38	0.39	0.37
27	H-N(5)	H$_{Re}$-C$_{\alpha}$(4)	0.22	0.23	0.22	0.22	0.22	0.22	0.21	0.23
28	H-N(5)	Me-C$_{\delta}$(4)	0.45	0.42	0.35	0.35	0.36	0.35	0.38	0.33
29	H-N(5)	H-C$_{\beta}$(5)	0.35	-	0.28	0.28	0.28	0.28	0.28	0.27
30	H-N(5)	H$_{Re}$-C$_{\alpha}$(5)	0.25	0.28	0.25	0.25	0.24	0.25	0.26	0.27
31	H-N(5)	H-N(6)	0.35	-	0.40	0.39	0.40	0.39	0.44	0.39
32	H-C$_{\beta}$(5)	H$_{Re}$-C$_{\alpha}$(2)	0.23	0.23	0.26	0.26	0.25	0.25	*0.43*	*0.46*
33	H-C$_{\beta}$(5)	H-C$_{\gamma}$(5)	0.26	0.25	0.25	0.25	0.24	0.24	0.24	0.25
34	H-C$_{\beta}$(5)	H$_{Si}$-C$_{\alpha}$(5)	0.25	0.23	0.24	0.24	0.24	0.24	0.24	0.25
35	H-N(6)	H-C$_{\beta}$(6)	0.29	-	0.28	0.28	0.28	0.28	0.25	0.25
36	H-N(6)	H$_{Re}$-C$_{\alpha}$(6)	0.25	0.27	0.24	0.24	0.25	0.26	0.28	0.29
37	H-N(6)	H$_{Re}$-C$_{\alpha}$(5)	0.22	0.25	0.23	0.22	0.24	0.23	0.28	0.25
38	H-C$_{\beta}$(6)	H$_{Re}$-C$_{\alpha}$(3)	0.25	0.23	0.25	0.25	0.27	0.27	0.35	*0.52*
39	H-C$_{\beta}$(6)	H$_{Si}$-C$_{\alpha}$(6)	0.26	0.23	0.24	0.24	0.24	0.23	0.24	0.23
40	H-N(7)	H$_{Re}$-C$_{\alpha}$(6)	0.24	0.26	0.24	0.24	0.22	0.24	0.25	0.30
41	H-N(7)	H-C$_{\beta}$(7)	0.30	-	0.28	0.28	0.28	0.24	0.28	0.27
42	H-N(7)	H$_{Re}$-C$_{\alpha}$(7)	0.27	0.27	0.25	0.25	0.25	0.26	0.27	0.26
43	H-C$_{\gamma}$(1)	H$_{Si}$-C$_{\alpha}$(1)	-	0.27	0.26	0.26	0.26	0.27	0.26	0.26
44	H-C$_{\gamma}$(1)	H$_{Re}$-C$_{\alpha}$(1)	-	0.31	0.30	0.30	0.29	0.29	0.28	0.29
45	H-C$_{\beta}$(1)	H-C$_{\gamma}$(1)	-	0.24	0.24	0.24	0.24	0.24	0.25	0.25
46	H$_{Si}$-C$_{\alpha}$(2)	Me-C$_{\gamma}$(2)	-	0.41	0.29	0.29	0.28	0.29	0.30	0.29
47	H$_{Re}$-C$_{\alpha}$(2)	Me-C$_{\gamma}$(2)	-	0.41	0.29	0.29	0.29	0.29	0.29	0.30
48	H$_{Re}$-C$_{\alpha}$(2)	H-N(2)	-	0.28	0.25	0.25	0.24	0.24	0.29	0.27
49	H-C$_{\beta}$(2)	H$_{Re}$-C$_{\alpha}$(2)	-	0.31	0.29	0.28	0.28	0.28	0.27	0.26
50	Me-C$_{\gamma}$(2)	H-N(2)	-	0.51	0.30	0.30	0.32	0.31	0.30	0.29
51	H$_{Si}$-C$_{\alpha}$(3)	H-C$_{\beta}$(3)	-	0.23	0.24	0.24	0.24	0.23	0.24	0.24
52	H$_{Si}$-C$_{\alpha}$(3)	H-C$_{\delta}$(3)	-	0.29	0.23	0.22	0.24	0.24	0.25	0.26
53	H$_{Si}$-C$_{\alpha}$(3)	H$_{Si}$-C$_{\gamma}$(3)	-	0.27	0.33	0.34	0.31	0.33	0.32	0.31
54	H$_{Si}$-C$_{\alpha}$(3)	Me-C$_{\varepsilon 2}$(3)	-	0.54	0.42	0.42	0.43	0.44	0.44	0.45
55	Me-C$_{\varepsilon 1}$(3)	H-C$_{\beta}$(3)	-	0.41	0.31	0.30	0.32	0.31	0.33	0.32
56	H-C$_{\beta}$(3)	Me-C$_{\varepsilon 2}$(3)	-	0.47	0.35	0.36	0.34	0.34	0.32	0.33
57	H-C$_{\beta}$(3)	H$_{Re}$-C$_{\gamma}$(3)	-	0.31	0.27	0.27	0.26	0.27	0.26	0.26

58	H-C_{β}(3)	H_{Si}-C_{γ}(3)	-	0.27	0.25	0.25	0.26	0.26	0.26	0.26
59	H-C_{δ}(3)	H-N(3)	-	0.38	0.37	0.38	0.34	0.34	0.30	0.27
60	Me-$C_{\varepsilon1}$(3)	H_{Si}-C_{γ}(3)	-	0.42	0.28	0.28	0.28	0.28	0.28	0.28
61	H_{Re}-C_{γ}(3)	H_{Si}-C_{α}(3)	-	0.35	0.26	0.27	0.25	0.26	0.25	0.25
62	H_{Re}-C_{γ}(3)	H_{Re}-C_{α}(3)	-	0.26	0.24	0.24	0.25	0.25	0.26	0.26
63	H_{Re}-C_{γ}(3)	H-C_{δ}(3)	-	0.24	0.25	0.25	0.25	0.25	0.26	0.26
64	H_{Si}-C_{γ}(3)	H-C_{δ}(3)	-	0.26	0.27	0.27	0.26	0.26	0.26	0.26
65	H_{Re}-C_{γ}(3)	Me-$C_{\varepsilon1}$(3)	-	0.47	0.34	0.34	0.33	0.33	0.32	0.32
66	H_{Re}-C_{γ}(3)	H-N(3)	-	0.34	0.28	0.28	0.29	0.28	0.28	0.30
67	H_{Si}-C_{γ}(3)	H_{Re}-C_{α}(3)	-	0.30	0.30	0.30	0.28	0.29	0.28	0.31
68	H_{Si}-C_{γ}(3)	H-N(3)	-	0.43	0.27	0.28	0.26	0.25	0.24	0.25
69	H_{Re}-C_{α}(4)	Me-C_{γ}(4)	-	0.40	0.28	0.28	0.29	0.29	0.27	0.28
70	H-N(4)	H_{Re}-C_{α}(4)	-	0.29	0.25	0.25	0.30	0.27	0.33	0.29
71	H-C_{β}(4)	H_{Re}-C_{α}(4)	-	0.28	0.28	0.28	0.28	0.28	0.24	0.25
72	H-C_{β}(4)	Me-C_{δ}(4)	-	0.40	0.29	0.29	0.29	0.29	0.27	0.28
73	H-C_{β}(4)	Me-C_{γ}(4)	-	0.38	0.24	0.24	0.24	0.24	0.24	0.24
74	H_{Si}-C_{α}(5)	Me-$C_{\delta2}$(5)	-	0.41	0.33	0.33	0.32	0.32	0.32	0.32
75	H-C_{γ}(5)	H_{Si}-C_{α}(5)	-	0.28	0.26	0.26	0.24	0.26	0.26	0.25
76	H_{Re}-C_{α}(5)	Me-$C_{\delta2}$(5)	-	0.45	0.34	0.34	0.32	0.32	0.34	0.35
77	H_{Re}-C_{α}(5)	H-C_{γ}(5)	-	0.28	0.27	0.27	0.28	0.28	0.29	0.29
78	H-C_{β}(5)	H_{Re}-C_{α}(5)	-	0.31	0.29	0.29	0.29	0.28	0.28	0.27
79	H-C_{β}(5)	Me-$C_{\delta2}$(5)	-	0.39	0.30	0.30	0.31	0.31	0.30	0.31
80	Me-$C_{\delta2}$(5)	H-N(5)	-	0.48	0.33	0.33	0.33	0.33	0.31	0.30
81	Me-$C_{\delta1}$(5)	H-C_{γ}(5)	-	0.36	0.24	0.24	0.24	0.24	0.24	0.24
82	H-C_{γ}(5)	H-N(5)	-	0.32	0.28	0.28	0.32	0.31	0.27	0.27
83	H_{Si}-C_{α}(6)	Me-C_{γ}(6)	-	0.41	0.28	0.28	0.28	0.30	0.28	0.30
84	H_{Re}-C_{α}(6)	H-C_{β}(6)	-	0.31	0.28	0.28	0.28	0.28	0.27	0.25
85	Me-C_{γ}(6)	H_{Re}-C_{α}(6)	-	0.44	0.30	0.30	0.30	0.28	0.30	0.29
86	H-C_{β}(6)	Me-C_{γ}(6)	-	0.28	0.24	0.24	0.24	0.24	0.24	0.24
87	Me-C_{γ}(6)	H-N(6)	-	0.38	0.29	0.29	0.29	0.29	0.28	0.29
88	H_{Si}-C_{α}(7)	H-C_{β}(7)	-	0.25	0.25	0.25	0.24	0.24	0.24	0.24
89	H_{Si}-C_{α}(7)	H-C_{δ}(7)	-	0.27	0.23	0.23	0.25	0.25	0.27	0.24

90	H_{Si}-C_{γ}(7)	H_{Si}-C_{α}(7)	-	0.26	0.30	0.30	0.30	0.30	0.34	0.31
91	H_{Si}-C_{α}(7)	H-N(7)	-	0.35	0.32	0.32	0.36	0.34	0.31	0.34
92	H_{Re}-C_{γ}(7)	H_{Re}-C_{α}(7)	-	0.28	0.27	0.27	0.27	0.27	0.26	0.26
93	H_{Re}-C_{α}(7)	H_{Si}-C_{γ}(7)	-	0.28	0.31	0.31	0.28	0.29	0.30	0.30
94	H-C_{β}(7)	Me-$C_{\varepsilon2}$(7)	-	0.39	0.33	0.33	0.32	0.32	0.33	0.34
95	H_{Re}-C_{γ}(7)	H-C_{β}(7)	-	0.29	0.26	0.26	0.26	0.26	0.26	0.27
96	H-C_{β}(7)	H_{Si}-C_{γ}(7)	-	0.26	0.26	0.26	0.27	0.26	0.26	0.26
97	H-C_{δ}(7)	H-N(7)	-	0.36	0.33	0.33	0.29	0.31	0.34	0.31
98	Me-$C_{\varepsilon2}$(7)	H-N(7)	-	0.64	0.48	0.47	0.43	0.44	0.48	0.42
99	Me-$C_{\varepsilon1}$(7)	H-C_{δ}(7)	-	0.35	0.24	0.24	0.24	0.24	0.24	0.24
100	Me-$C_{\varepsilon2}$(7)	H_{Si}-C_{γ}(7)	-	0.42	0.32	0.32	0.32	0.32	0.32	0.31
101	Me-$C_{\varepsilon1}$(7)	H_{Si}-C_{γ}(7)	-	0.41	0.28	0.28	0.28	0.28	0.28	0.28
102	Me-$C_{\varepsilon1}$(7)	H_{Re}-C_{γ}(7)	-	0.39	0.33	0.33	0.32	0.32	0.33	0.33
103	H_{Re}-C_{γ}(7)	H-N(7)	-	0.32	0.28	0.28	0.29	0.30	0.29	0.28
104	H_{Si}-C_{γ}(7)	H-N(7)	-	0.39	0.25	0.25	0.24	0.26	0.26	0.25
105	H-C_{β}(1)	H-N(2)	-	0.48	0.42	0.41	0.32	0.33	0.31	0.28
106	H-C_{β}(2)	H-C_{δ}(3)	-	0.44	*0.65*	*0.62*	0.54	*0.59*	0.49	0.46
107	H-C_{β}(2)	H-N(3)	-	0.41	0.43	0.42	0.43	0.39	0.28	0.24
108	H-N(2)	H-C_{β}(3)	-	0.45	0.48	0.48	0.49	0.48	0.47	0.52
109	H-C_{β}(4)	H-N(5)	-	0.42	0.44	0.44	0.43	0.43	0.42	0.29
110	H-N(4)	H-C_{β}(5)	-	0.45	0.47	0.47	0.48	0.47	0.43	0.46
111	H-N(4)	Me-$C_{\delta1}$(5)	-	0.57	*0.70*	*0.70*	*0.69*	*0.68*	0.65	0.61
112	H_{Si}-C_{α}(5)	H-N(6)	-	0.34	0.27	0.28	0.24	0.26	0.24	0.26
113	H-C_{β}(5)	H-N(6)	-	0.43	0.42	0.44	0.41	0.43	0.24	0.24
114	H-N(5)	H-C_{β}(6)	-	0.44	0.50	0.50	0.50	0.48	0.54	0.54
115	H-N(6)	H-C_{β}(7)	-	0.42	*0.54*	*0.54*	0.51	0.51	*0.55*	0.43
116	H-C_{β}(6)	H-N(7)	-	0.42	0.39	0.41	0.41	0.30	0.23	0.29
117	H-C_{γ}(1)	H-C_{β}(4)	-	0.35	0.39	0.40	0.42	0.39	*0.48*	*0.72*
118	H-N(2)	Me-C_{δ}(4)	-	0.60	0.48	0.48	0.49	0.49	0.58	0.62
119	Me-C_{γ}(2)	H-C_{β}(5)	-	0.44	0.37	0.37	0.40	0.38	*0.62*	*0.60*
120	Me-C_{γ}(2)	Me-$C_{\delta2}$(5)	-	0.63	0.45	0.46	0.56	0.48	*0.93*	0.66
121	H-N(2)	Me-$C_{\delta2}$(5)	-	0.58	0.48	0.48	0.53	0.52	*0.88*	0.67

122	H-N(2)	H-C_γ(5)	-	0.53	0.48	0.48	0.47	0.46	*0.86*	*0.65*
123	H-N(3)	Me-$C_{\delta2}$(5)	-	0.60	0.65	0.64	0.68	0.65	*1.01*	0.64
124	H-N(3)	Me-C_γ(6)	-	0.62	0.47	0.47	0.45	0.47	0.67	0.54
125	H_{Re}-C_γ(3)	H-C_β(6)	-	0.29	0.28	0.27	0.34	0.31	0.39	*0.61*
126	H_{Si}-C_γ(3)	H-C_β(6)	-	0.32	0.32	0.32	0.33	0.31	*0.45*	*0.56*
127	H-N(3)	H_{Si}-C_α(6)	-	0.45	0.52	0.53	0.49	0.46	*0.66*	0.49
128	H_{Re}-C_α(4)	H-C_β(7)	-	0.24	0.32	0.31	*0.37*	0.32	*0.50*	*0.68*
129	Me-C_γ(4)	H-C_β(7)	-	0.45	0.37	0.37	0.47	0.41	*0.56*	*0.73*
130	Me-C_γ(4)	Me-$C_{\varepsilon1}$(7)	-	0.65	0.41	0.40	0.53	0.45	0.55	*0.78*
131	H-N(5)	H-C_β(7)	-	0.36	0.44	0.43	*0.47*	0.42	*0.62*	*0.61*
Nviol42					0	0	1	0	6	9
RMSD42					0.01	0.01	0.03	0.01	0.08	0.10
Nviol119					3	3	3	2	18	18
RMSD119					0.03	0.03	0.03	0.03	0.09	0.10

Table S15. List of 21 ^3J-coupling constants (Hz) derived from experiment and averaged from unrestrained and RDC-restrained (ΔD^{fb} = 2.0 Hz) $t^{msy} = t^{AT}$ = 100 ns MD simulations of the ß-heptapeptide. *MDsol*: MD simulation of the peptide solvated in methanol without any restraining of the molecule ($K^{RDC,msy}$ = 0 kJmol^{-1}Hz^{-2} using the *HRS* method). *HRSrMDsol*: RDC-restraining MD simulations with $K^{RDC,msy}$ = 0.05 kJmol^{-1}Hz^{-2} and $\tau_\theta^{RDC,msy}$ = 1 ns. *SDvac*: SD simulation of the peptide in vacuo without any restraining of the molecule ($K^{RDC,msy}$ = 0 kJmol^{-1}Hz^{-2} using the *HRS* method). *HRSrSDvac*: RDC-restraining SD simulations with $K^{RDC,msy}$ = 0.05 kJmol^{-1}Hz^{-2} and $\tau_\theta^{RDC,msy}$ = 1 ns. The *mfv* parameters of the *HRS* method have the values γ^{mfv} = 2.4 ps^{-1}, $K^{RDC,mfv}$ = 100 kJmol^{-1}Hz^{-2}, $\tau_\theta^{RDC,mfv}$ = 100 ns and N_{mfv} = 100. *ATrSDvac*: RDC-restraining SD simulations of the peptide in vacuo using the alignment-tensor (*AT*) formalism with $\tau_D^{RDC} = \tau_{AT}^{RDC}$ = 0 and $K^{RDC,AT}$ = 0.1 or 10 kJmol^{-1}Hz^{-2}. *Ndev*: number of deviations between simulation and experiment larger than 2 Hz. *RMSD*: root-mean-square difference between experiment (Ref.[45]) and simulation for the 21 ^3J-coupling constants (Hz). The residue sequence numbers of the atoms are within parentheses. Experimentally derived ^3J-couplings from Table 2 of Ref.[45] and from Tables 2 and 3 of Ref.[24]. MD values differing more than 2 Hz from the experimental value are in (red) italics.

^3J-coupling sequence number	^3J-coupling dihedral angle	Exp. value (Hz)		^3J-coupling (Hz)					
Simulation		Ref. [45]	Ref. [24]	*MDsol*	*HRSrMDsol*	*SDvac*	*HRSrSDvac*	*ATrSDvac*	
$K^{RDC,AT}$	kJmol^{-1}Hz^{-2}							0.1	10
1	$H_ß(1)$-$C_ß(1)$-$C_α(1)$-$H_{αSi}(1)$	2.8		2.6	2.7	3.1	3.1	3.4	3.4
2	$H_ß(2)$-$C_ß(2)$-$C_α(2)$-$H_{αSi}(2)$	4.5	4.4	3.7	3.8	3.4	3.6	4.2	4.2
3	$H_ß(3)$-$C_ß(3)$-$C_α(3)$-$H_{αSi}(3)$	4.5	4.2	3.9	4.0	3.6	3.8	3.7	4.1
4	$H_ß(5)$-$C_ß(5)$-$C_α(5)$-$H_{αSi}(5)$	3.9	3.7	3.4	3.4	3.3	3.8	3.6	4.6
5	$H_ß(6)$-$C_ß(6)$-$C_α(6)$-$H_{αSi}(6)$	3.8	4.1	3.3	3.2	3.0	4.4	5.2	4.8

6	H$_{\beta}$(7)-C$_{\beta}$(7)-C$_{\alpha}$(7)-H$_{\alpha \mathit{Si}}$(7)	4.5		4.1	4.1	2.7	3.2	3.5	3.3
7	H$_{\beta}$(1)-C$_{\beta}$(1)-C$_{\gamma}$(1)-H$_{\gamma}$(1)	4.7	5.1	5.0	4.4	5.0	5.6	6.3	6.0
8	H$_{\beta}$(5)-C$_{\beta}$(5)-C$_{\gamma}$(5)-H$_{\gamma}$(5)	7.0	7.0	6.4	6.2	*4.0*	*4.2*	5.1	5.6
9	H$_{N}$(2)-N(2)-C$_{\beta}$(2)-H$_{\beta}$(2)	9.2	9.1	9.2	9.2	7.3	7.8	8.0	8.0
10	H$_{N}$(3)-N(3)-C$_{\beta}$(3)-H$_{\beta}$(3)	9.6	9.2	9.1	9.1	9.1	8.4	8.9	*6.7*
11	H$_{N}$(4)-N(4)-C$_{\beta}$(4)-H$_{\beta}$(4)	9.3	9.0	9.3	9.3	7.2	8.0	8.6	8.2
12	H$_{N}$(5)-N(5)-C$_{\beta}$(5)-H$_{\beta}$(5)	9.6	9.4	9.3	9.3	8.7	8.8	9.0	8.0
13	H$_{N}$(6)-N(6)-C$_{\beta}$(6)-H$_{\beta}$(6)	8.7	8.6	9.2	9.2	8.5	8.6	8.0	*6.4*
14	H$_{N}$(7)-N(7)-C$_{\beta}$(7)-H$_{\beta}$(7)	9.5	9.3	9.2	9.2	9.0	8.3	8.7	8.8
15	H$_{\beta}$(1)-C$_{\beta}$(1)-C$_{\alpha}$(1)-H$_{\alpha \mathit{Re}}$(1)	11.5	11.7	11.9	11.8	12.2	12.3	11.3	11.3
16	H$_{\beta}$(2)-C$_{\beta}$(2)-C$_{\alpha}$(2)-H$_{\alpha \mathit{Re}}$(2)	12.0	12.1	12.6	12.3	12.3	12.2	*9.6*	*8.1*
17	H$_{\beta}$(3)-C$_{\beta}$(3)-C$_{\alpha}$(3)-H$_{\alpha \mathit{Re}}$(3)	12.3		12.5	12.6	11.6	*10.1*	11.4	*7.7*
18	H$_{\beta}$(4)-C$_{\beta}$(4)-C$_{\alpha}$(4)-H$_{\alpha \mathit{Re}}$(4)	10.8	10.9	12.4	12.4	12.4	12.0	*3.2*	*5.0*
19	H$_{\beta}$(5)-C$_{\beta}$(5)-C$_{\alpha}$(5)-H$_{\alpha \mathit{Re}}$(5)	12.3	12.2	12.5	12.6	12.3	12.1	11.2	*8.5*
20	H$_{\beta}$(6)-C$_{\beta}$(6)-C$_{\alpha}$(6)-H$_{\alpha \mathit{Re}}$(6)	11.6	11.4	11.4	11.6	12.1	11.3	*9.2*	*7.0*
21	H$_{\beta}$(7)-C$_{\beta}$(7)-C$_{\alpha}$(7)-H$_{\alpha \mathit{Re}}$(7)	10.0		9.4	9.6	11.8	10.5	*7.7*	10.6
Ndev				0	0	1	2	4	7
RMSD				0.5	0.5	1.2	1.1	*2.1*	*2.5*

Table S16. Averages and root-mean-square fluctuations (RMSF) of 19 backbone and 6 side-chain torsional angles (degree) from unrestrained and RDC-restrained (ΔD^{fb} = 2.0 Hz) t^{msy} = t^{AT} = 100 ns MD simulations of the ß-heptapeptide. *MDsol*: MD simulation of the peptide solvated in methanol without any restraining of the molecule ($K^{RDC,msy}$ = 0 kJmol^{-1}Hz^{-2} using the *HRS* method). *HRSrMDsol*: RDC-restraining MD simulations with $K^{RDC,msy}$ = 0.05 kJmol^{-1}Hz^{-2} and $\tau_{\theta}^{RDC,msy}$ = 1 ns. *SDvac*: SD simulation of the peptide in vacuo without any restraining of the molecule ($K^{RDC,msy}$ = 0 kJmol^{-1}Hz^{-2} using the *HRS* method). *HRSrSDvac*: RDC-restraining SD simulations with $K^{RDC,msy}$ = 0.05 kJmol^{-1}Hz^{-2} and $\tau_{\theta}^{RDC,msy}$ = 1 ns. The *mfv* parameters of the *HRS* method have the values γ^{mfv} = 2.4 ps^{-1}, $K^{RDC,mfv}$ = 100 kJmol^{-1}Hz^{-2}, $\tau_{\theta}^{RDC,mfv}$ = 100 ns and N_{mfv} = 100. *ATrSDvac*: RDC-restraining SD simulations of the peptide in vacuo using the alignment-tensor (*AT*) formalism with τ_{D}^{RDC} = τ_{AT}^{RDC} = 0 and $K^{RDC,AT}$ = 0.1 or 10 kJmol^{-1}Hz^{-2}. The residue sequence numbers of the atoms are within parentheses.

	Torsional angle	Average angle and fluctuations (degree)											
Simulation		*MDsol*		*HRSrMDsol*		*SDvac*		*HRSrSDvac*		*ATrSDvac*			
				$K^{RDC,msy}$ = 0.05 kJmol^{-1}Hz^{-2}				$K^{RDC,msy}$ = 0.05 kJmol^{-1}Hz^{-2}		$K^{RDC,AT}$ = 0.1 kJmol^{-1}Hz^{-2}		$K^{RDC,AT}$ = 10 kJmol^{-1}Hz^{-2}	
		<angle>	*RMSF*	<angle>	*RMSF*	<angle>	*RMSF*	<angle>	*RMSF*	<angle>	*RMSF*	<angle>	*RMSF*
backbone													
1	N(1)-Cß(1)-Cα(1)-C(1)	73	15	74	17	67	19	66	18	68	40	67	39
2	Cß(1)-Cα(1)-C(1)-N(2)	-52	147	-43	147	-28	108	-43	107	0	114	23	107
3	C(1)-N(2)-Cß(2)-Cα(2)	-126	17	-125	19	-135	68	-136	53	-52	86	-98	64
4	N(2)-Cß(2)-Cα(2)-C(2)	58	11	55	23	59	20	58	21	27	50	36	74
5	Cß(2)-Cα(2)-C(2)-N(3)	-132	17	-117	67	-26	159	-100	109	-33	86	-13	100
6	C(2)-N(3)-Cß(3)-Cα(3)	-128	15	-129	15	-119	20	-114	31	-105	18	-50	85
7	N(3)-Cß(3)-Cα(3)-C(3)	58	11	56	9	53	33	36	49	50	39	33	74
8	Cß(3)-Cα(3)-C(3)-N(4)	-119	72	-142	10	-17	73	-59	100	53	79	-20	93

9	$C(3)-N(4)-C_{\beta}(4)-C_{\alpha}(4)$	-124	11	-125	10	17	82	-55	96	-123	27	-107	67
10	$N(4)-C_{\beta}(4)-C_{\alpha}(4)-C(4)$	51	12	51	9	51	10	48	20	-41	27	-5	72
11	$C_{\beta}(4)-C_{\alpha}(4)-C(4)-N(5)$	-140	11	-140	10	-136	12	-138	16	-111	22	-67	82
12	$C(4)-N(5)-C_{\beta}(5)-C_{\alpha}(5)$	-123	10	-123	9	-132	16	-128	18	-105	17	-88	44
13	$N(5)-C_{\beta}(5)-C_{\alpha}(5)-C(5)$	62	12	61	10	63	15	55	22	70	28	76	57
14	$C_{\beta}(5)-C_{\alpha}(5)-C(5)-N(6)$	-106	99	-129	69	-8	152	-78	125	52	83	-7	90
15	$C(5)-N(6)-C_{\beta}(6)-C_{\alpha}(6)$	-125	17	-124	14	-115	58	-121	38	-75	60	-67	92
16	$N(6)-C_{\beta}(6)-C_{\alpha}(6)-C(6)$	69	36	69	30	65	19	45	31	78	52	17	81
17	$C_{\beta}(6)-C_{\alpha}(6)-C(6)-N(7)$	-2	142	-6	145	-117	39	-43	115	-20	76	9	128
18	$C(6)-N(7)-C_{\beta}(7)-C_{\alpha}(7)$	-116	18	-117	18	-109	18	-65	84	-123	32	-105	49
19	$N(7)-C_{\beta}(7)-C_{\alpha}(7)-C(7)$	68	62	69	59	70	20	57	45	7	62	55	47
side-chains													
20	$C_{\alpha}(1)-C_{\beta}(1)-C_{\gamma}(1)-C_{\delta1}(1)$	28	96	31	101	46	105	38	98	60	97	49	97
21	$C_{\alpha}(3)-C_{\beta}(3)-C_{\gamma}(3)-C_{\delta}(3)$	79	70	79	64	82	89	85	75	88	100	104	70
22	$C_{\beta}(3)-C_{\gamma}(3)-C_{\delta}(3)-C_{\varepsilon1}(3)$	80	68	78	64	80	87	77	80	82	98	84	90
23	$C_{\alpha}(5)-C_{\beta}(5)-C_{\gamma}(5)-C_{\delta1}(5)$	50	92	55	93	72	104	48	105	52	102	72	97
24	$C_{\alpha}(7)-C_{\beta}(7)-C_{\gamma}(7)-C_{\delta}(7)$	90	84	86	86	94	99	96	90	91	78	83	80
25	$C_{\beta}(7)-C_{\gamma}(7)-C_{\delta}(7)-C_{\varepsilon1}(7)$	84	85	85	84	78	104	82	95	79	85	79	79

Figures S1, S2

Figure S1. Distribution of the angle $\theta_{k_1k_2,H}$ between 10 RDC-vectors for $D_{k_1k_2}$ and the magnetic-field direction $\vec{H}$ from MD simulations of the ß-heptapeptide solvated in methanol without any restraining of the molecule ($K^{RDC,msy}$ = 0 using the *HRS*-method). Parameter values of the t^{msy} = 100 ns *HRS* simulations are γ^{mfv} = 2.4 ps^{-1}, ΔD^{fb} = 2.0 Hz, $\tau_{\theta}^{RDC,mfv}$ = 10 ns, while the values of the parameters N_{mfv} and $K^{RDC,mfv}$ (kJmol^{-1}Hz^{-2}) were varied. The residue sequence numbers of the atoms are indicated at the atom names. Top panel: N_{mfv} = 10. Middle panel: N_{mfv} = 100. Bottom panel: N_{mfv} = 1000. $K^{RDC,mfv}$ = 10 (solid blue lines), 100 (red dotted lines), 1000 (green dashed lines) kJmol^{-1}Hz^{-2}.

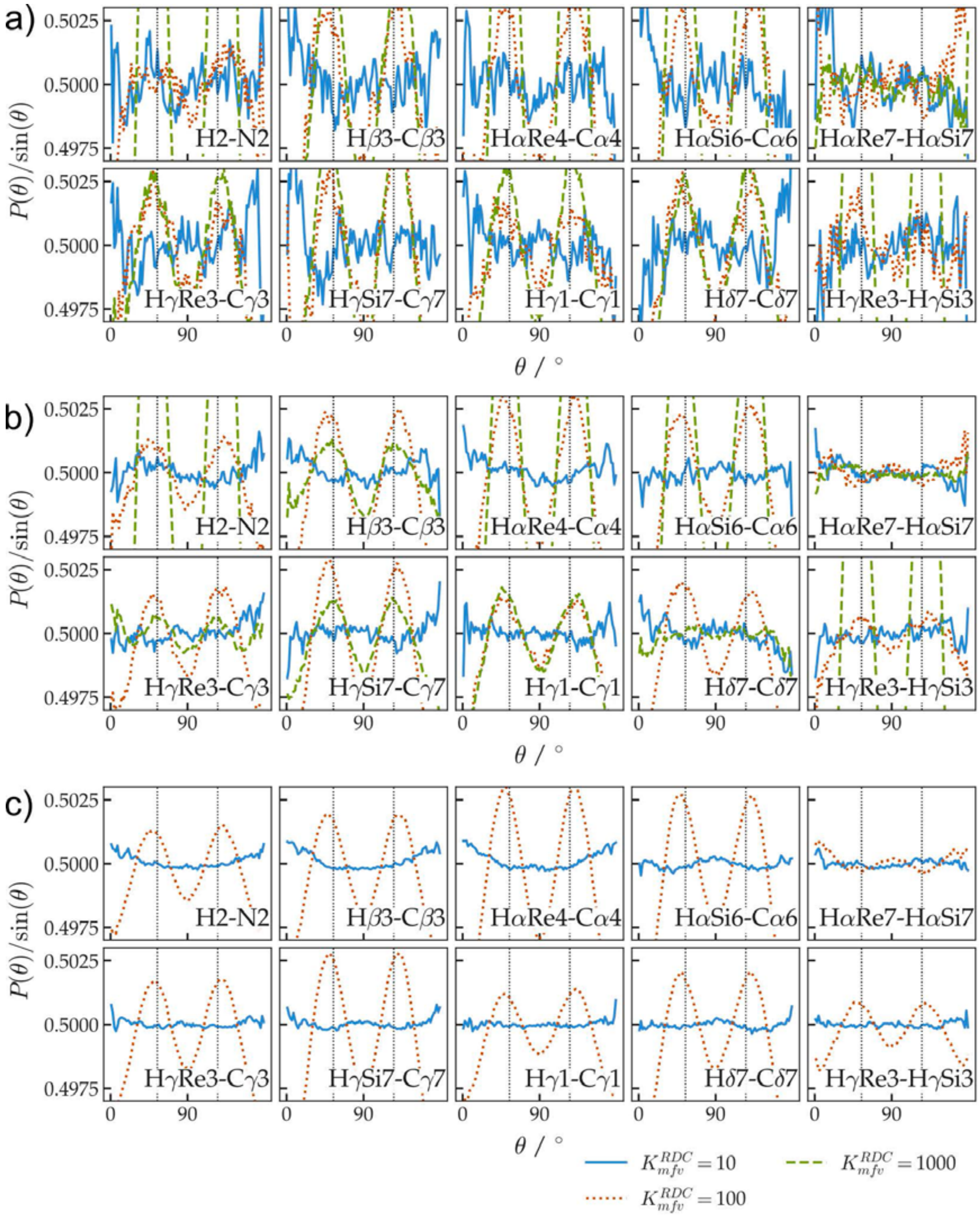
a)
b)
c)
$P(\theta)/\sin(\theta)$
0.5025
0.5000
0.4975
H2-N2
Hβ3-Cβ3
HαRe4-Cα4
HαSi6-Cα6
HαRe7-HαSi7
HγRe3-Cγ3
HγSi7-Cγ7
Hγ1-Cγ1
Hδ7-Cδ7
HγRe3-HγSi3
0
90
θ / °
$K_{mfv}^{RDC} = 10$
$K_{mfv}^{RDC} = 100$
$K_{mfv}^{RDC} = 1000$

Figure S2. Distribution of the angle $\theta_{k_1k_2,H}$ between 10 RDC-vectors for $D_{k_1k_2}$ and the magnetic-field direction $\vec{H}$ from MD simulations of the ß-heptapeptide solvated in methanol without any restraining of the molecule ($K^{RDC,msy}$ = 0 using the *HRS*-method). Parameter values of the t^{msy} = 100 ns *HRS* simulations are γ^{mfv} = 2.4 ps^{-1}, ΔD^{fb} = 2.0 Hz, $\tau_{\theta}^{RDC,mfv}$ = 100 ns, while the values of the parameters N_{mfv} and $K^{RDC,mfv}$ (kJmol^{-1}Hz^{-2}) were varied. The residue sequence numbers of the atoms are indicated at the atom names. Top panel: N_{mfv} = 10. Middle panel: N_{mfv} = 100. Bottom panel: N_{mfv} = 1000. $K^{RDC,mfv}$ = 10 (solid blue lines), 100 (red dotted lines), 1000 (green dashed lines) kJmol^{-1}Hz^{-2}.

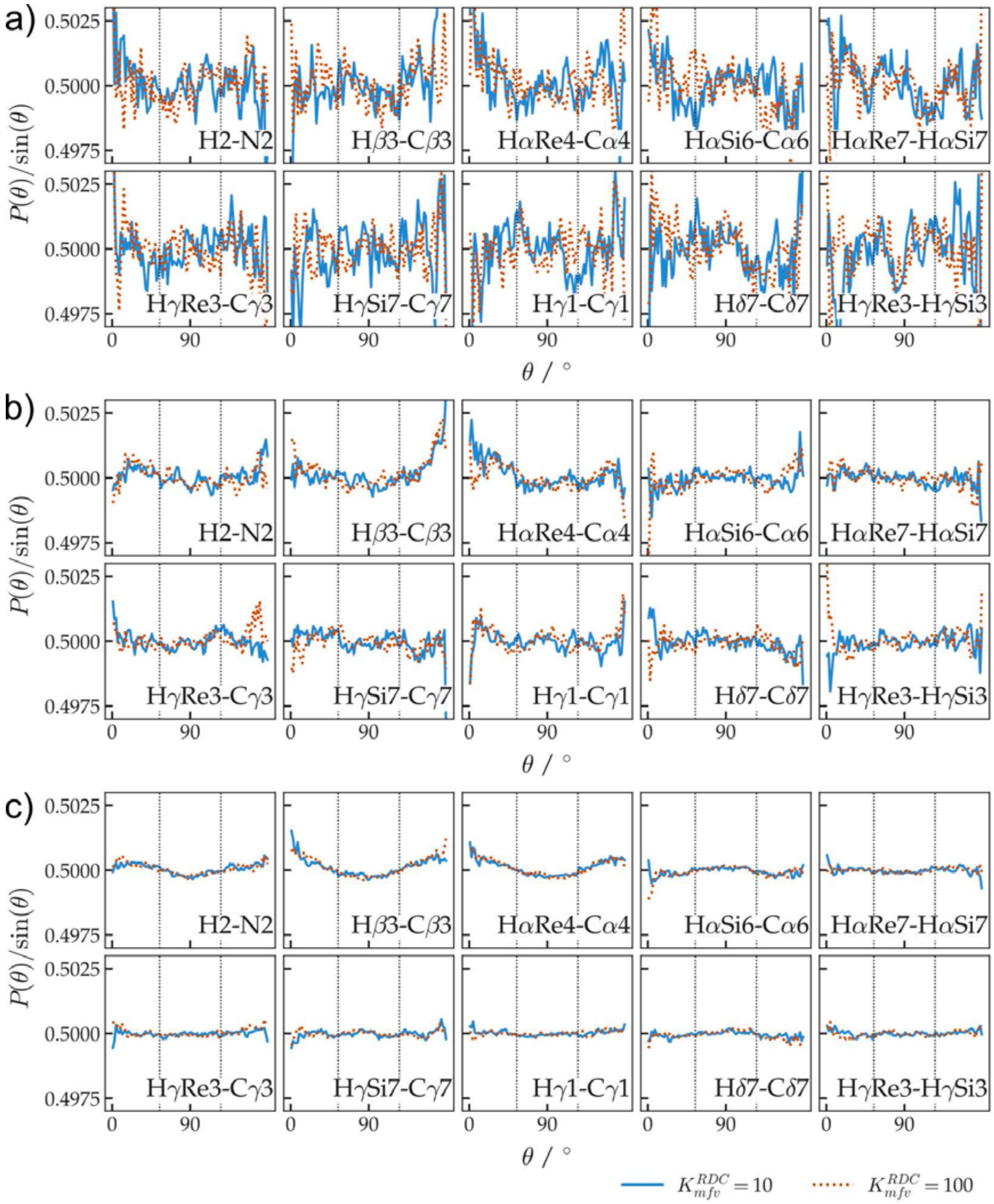
a)
b)
c)
0.5025
0.5000
0.4975
$P(\theta)/\sin(\theta)$
H2-N2
Hβ3-Cβ3
HαRe4-Cα4
HαSi6-Cα6
HαRe7-HαSi7
HγRe3-Cγ3
HγSi7-Cγ7
Hγ1-Cγ1
Hδ7-Cδ7
HγRe3-HγSi3
0
90
θ / °
$K_{mfv}^{RDC} = 10$
$K_{mfv}^{RDC} = 100$
